# A complete diploid human genome benchmark for personalized genomics

**DOI:** 10.1016/j.cell.2026.06.016

**Published:** 2026-08-06

**Authors:** Nancy F. Hansen, Nathan Dwarshuis, Hyun Joo Ji, Arang Rhie, Hailey Loucks, Glennis A. Logsdon, Mitchell R. Vollger, Jessica M. Storer, Juhyun Kim, Eleni Adam, Nicolas Altemose, Dmitry Antipov, Mobin Asri, Sofia Barreira, Stephanie C. Bohaczuk, Andrey V. Bzikadze, Sara A. Carioscia, Andrew Carroll, Kuan-Hao Chao, Yanan Chu, Arun Das, Peter Ebert, Adam English, Mark Fleharty, Laura E. Fleming, Giulio Formenti, Andrea Guarracino, Gabrielle A. Hartley, Katharine Jenike, Jenna Kalleberg, Yu Kang, Robert King, Josipa Lipovac, Mira Mastoras, Matthew W. Mitchell, Shloka Negi, Nathan D. Olson, Keisuke K. Oshima, Luis F. Paulin, Brandon D. Pickett, David Porubsk, Jane Ranchalis, Desh Ranjan, Mikko Rautiainen, Harold Riethman, Robert D. Schnabel, Fritz J. Sedlazeck, Kishwar Shafin, Mile Sikic, Steven J. Solar, Alexander P. Sweeten, Winston Timp, Justin Wagner, DongAhn Yoo, Ying Zhou, Erik Garrison, Evan E. Eichler, Michael C. Schatz, Andrew B. Stergachis, Rachel J. O’Neill, Karen H. Miga, Steven L. Salzberg, Sergey Koren, Justin M. Zook, Adam M. Phillippy

**Affiliations:** 1 Genome Informatics Section, Center for Genomics and Data Science Research, National Human Genome Research Institute, National Institutes of Health, Bethesda, MD, USA; 2 Material Measurement Laboratory, National Institute of Standards and Technology, Gaithersburg, MD, USA; 3 Department of Computer Science, Johns Hopkins University, Baltimore, MD, USA; 4 Center for Computational Biology, Johns Hopkins University, Baltimore, MD, USA; 5 University of California, Santa Cruz Genomics Institute, University of California, Santa Cruz, Santa Cruz, CA, USA; 6 Department of Genetics, Epigenetics Institute, Perelman School of Medicine, University of Pennsylvania, Philadelphia, PA, USA; 7 Division of Medical Genetics, Department of Medicine, University of Washington School of Medicine, Seattle, WA, USA; 8 Institute for Systems Genomics, University of Connecticut, Storrs, CT, USA; 9 Department of Computer Science, Old Dominion University, Norfolk, VA, USA; 10 Department of Genetics, School of Medicine, Stanford University, Palo Alto, CA, USA; 11 Chan Zuckerberg Biohub, San Francisco, CA, USA; 12 Computational Genomics Unit, Center for Genomics and Data Science Research, National Human Genome Research Institute, National Institutes of Health, Bethesda, MD, USA; 13 Graduate Program in Bioinformatics and Systems Biology, University of California, San Diego, La Jolla, CA, USA; 14 Department of Biology, Johns Hopkins University, Baltimore, MD, USA; 15 Google LLC, Mountain View, CA, USA; 16 Beijing Institute of Genomics, Chinese Academy of Sciences, China National Center for Bioinformation, Beijing 100101, China; 17 Core Unit Bioinformatics, Medical Faculty and University Hospital Düsseldorf, Heinrich-Heine-Universität Düsseldorf, Düsseldorf, Germany; 18 Center for Digital Medicine, Heinrich-Heine-Universität Düsseldorf, Düsseldorf, Germany; 19 Human Genome Sequencing Center, Baylor College of Medicine, Houston, TX, USA; 20 Broad Institute, Broad Clinical Labs, Burlington, MA, USA; 21 Vertebrate Genome Laboratory, the Rockefeller University, New York, NY, USA; 22 Bioinnovation and Genome Sciences, the Translational Genomics Research Institute (TGen), Phoenix, AZ 85004, USA; 23 Department of Plant Sciences, University of Cambridge, Cambridge CB2 3EA, UK; 24 Division of Animal Sciences, University of Missouri – Columbia, Columbia, MO, USA; 25 Oxford Nanopore Technologies, Oxford, UK; 26 Laboratory for Bioinformatics and Computational Biology, Faculty of Electrical Engineering and Computing, University of Zagreb, Zagreb, Croatia; 27 Coriell Institute for Medical Research, Camden, NJ, USA; 28 Department of Genome Sciences, University of Washington School of Medicine, Seattle, WA, USA; 29 Genome Biology Unit, European Molecular Biology Laboratory (EMBL), 69117 Heidelberg, Germany; 30 Institute for Molecular Medicine Finland, Helsinki Institute of Life Science, University of Helsinki, Helsinki, Finland; 31 School of Medical Diagnostic & Translational Sciences, Old Dominion University, Norfolk, VA, USA; 32 Department of Molecular and Human Genetics, Baylor College of Medicine, Houston, TX, USA; 33 Department of Computer Science, Rice University, Houston, TX, USA; 34 Genome Institute of Singapore, A*STAR, Singapore, Republic of Singapore; 35 Department of Biomedical Engineering, Johns Hopkins University, Baltimore, MD, USA; 36 Department of Data Science, Dana-Farber Cancer Institute, Boston, MA, USA; 37 Department of Genetics, Genomics, and Informatics, University of Tennessee Health Science Center, Memphis, TN, USA; 38 Howard Hughes Medical Institute, University of Washington, Seattle, WA, USA; 39 Brotman Baty Institute for Precision Medicine, Seattle, WA, USA; 40 Department of Molecular and Cell Biology, University of Connecticut, Storrs, CT, USA; 41 Department of Genome Sciences, UConn Health, Farmington, CT, USA; 42 Department of Biostatistics, Johns Hopkins University, Baltimore, MD, USA; 43 Department of Genetic Medicine, Johns Hopkins University School of Medicine, Baltimore, MD, USA; 44 Lead contact

## Abstract

Human genome sequencing typically relies on mapping reads to a reference genome to call variants, but this approach introduces technical biases, excluding duplicated and structurally polymorphic regions of the genome. To overcome this, we present a telomere-to-telomere genome benchmark with near-perfect accuracy across 99.4% of the diploid HG002 genome. This benchmark adds 701.4 Mb of autosomal sequence and both sex chromosomes (216.8 Mb), which were absent from prior benchmarks. We annotated genes and repeats on both haplotypes, including 19,956 protein-coding genes on the maternal haplotype and 19,190 on the paternal haplotype, and developed new methods to measure the accuracy of reads, phased variant call sets, and assemblies against a diploid reference. Genome-wide analyses show that *de novo* assembly resolves 2%–7% more sequence and outperforms variant calling accuracy by an order of magnitude, expanding the reach of genomic medicine to the entire genome and enabling a new era of personalized genomics.

## In brief

A complete, near-perfect diploid assembly of the HG002 benchmark provides a ground truth for the entire genome rather than a subset of variants. This fully annotated genome benchmark enables quality assessments in previously excluded regions and supports a transition toward the routine sequencing of complete, personalized genomes.

## Graphical Abstract

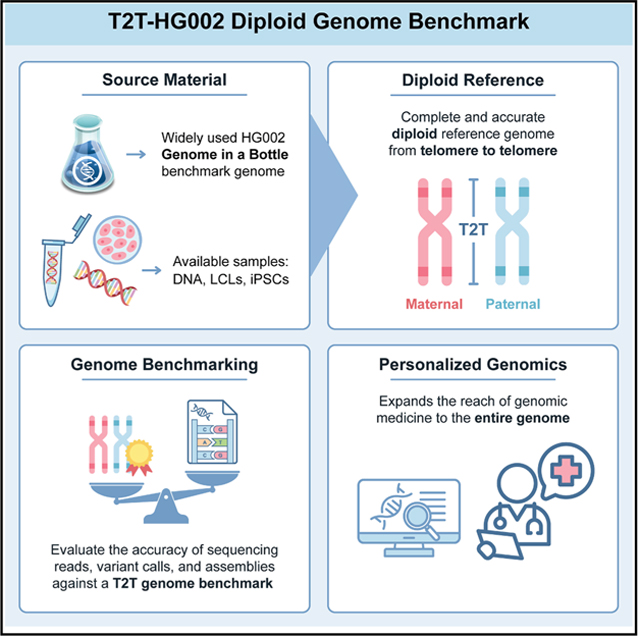

## INTRODUCTION

Variant benchmarks developed by the Genome in a Bottle (GIAB) Consortium have played a pivotal role in advancing the accuracy of human genome sequencing by providing a truth against which experimental protocols and analysis pipelines can be evaluated. ^1,2^ Motivated by competitive assessments, the top variant calling pipelines can now achieve F1 scores approaching 0.999 on the latest benchmarks, ^3^ appearing to leave little room for improvement. However, these variant benchmarks are limited in scope, as they currently exclude from consideration structural variation and the most polymorphic regions of the genome, which are often implicated in human genetic disease. For example, GIAB’s v4.2.1 small variant benchmark for the HG002 genome excluded both sex chromosomes and 12% of the autosomes. ^4^ The variant-centric framework has limitations, primarily due to the mapping-based approaches used to construct the benchmarks.

Although suitable for many applications, mapping-based resequencing fails to genotype many variants within duplicated or structurally polymorphic regions of the genome. For example, short-read sequencing alone is not able to phase variants by haplotype or resolve highly similar duplications, including multi-copy gene families. Linked or long reads partially address this technical bias, ^5,6^ and pedigrees can be used to further improve variant filtering in complex regions,^7^ but some bias persists whenever variants are called against a reference genome. This arises from errors, gaps, and natural variation in the reference that interfere with read mapping and variant representation, resulting in a lack of variant calls within sequences that are missing or otherwise unalignable to the reference. A complete reference genome, such as T2T-CHM13, addresses the problem of errors and gaps ^8,9^ but does not address the issue of structural variation between the sample and reference genomes. A pangenome reference provides a more diverse panel of haplotypes for mapping, thereby reducing reference bias. ^10^ However, both the development and validation of pangenome resources have relied on benchmarking against incomplete references such as GRCh38. ^11^ This creates a circular dependency, which makes it difficult to establish their accuracy in repetitive and polymorphic regions of the genome—precisely where pangenomes should provide the greatest benefit.

To address these challenges, sophisticated benchmarking tools have been developed to compare different representations of unphased small variants, ^12^ phased, complex variants, ^13^ and variants within tandem repeats. ^14^ However, regions with complex structural and copy-number variation remain difficult to assess. Therefore, even when these regions can be accurately resolved with *de novo* assembly, ^15^ they remain excluded from evaluation due to the lack of an appropriate benchmark. ^16^ For example, GIAB’s latest assembly-based benchmarks for HG002 exclude many major histocompatibility complex class II (MHC class II) genes^17^ as well as 122 medically relevant genes that are considered difficult to genotype due to the presence of complex or copy-number variants, repetitive sequences, and segmental duplications. ^18^

**Figure 1.**
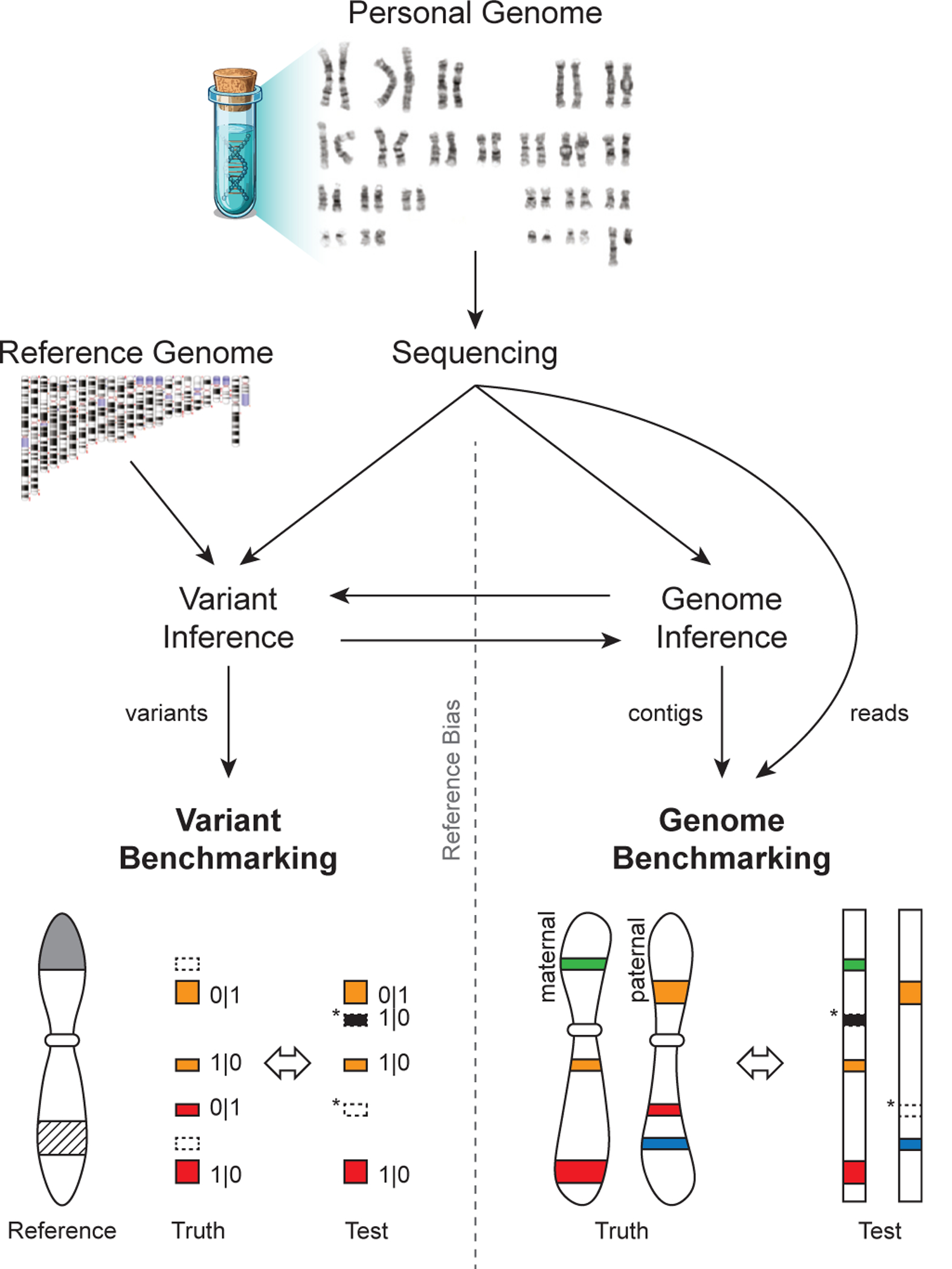
Variant benchmarking versus genome benchmarking *Variant inference* (i.e., variant calling) refers to the process of identifying a set of variants (differences) between the sequencing data and a reference genome. *Genome inference* refers to the process of constructing a genome sequence either *de novo* or with the assistance of a reference genome or pangenome. It is possible to move between the two frameworks, either by aligning contigs to a reference to call variants (right to left) or by applying a set of variants to a reference to construct contigs (left to right). *Variant benchmarking* compares a set of test variants against a set of known variants to identify FP and FN calls (asterisks). However, this approach is typically affected by *reference bias*, especially when the reference is a missing sequence (gray) or contains complex regions that are difficult to represent in the benchmark (hashed). *Genome benchmarking* compares a set of sequences (either assembled contigs or reads) directly to the complete, diploid genome they originated from, thus removing the need for a reference genome and enabling a more comprehensive evaluation free of reference bias.

To overcome these limitations, we introduce the concept of a telomere-to-telomere (T2T) “genome benchmark,” which uses the complete diploid genome sequence of the sample as a ground truth, rather than a set of variants against a reference genome (Figure 1). Genome benchmarking is more flexible and avoids the inherent biases of variant benchmarking, while simultaneously refocusing the objective away from variant calling and toward “genome inference,” where the goal is to directly infer the haplotypes of the genome being sequenced. ^19,20^ A complete diploid sequence is the most fundamental representation of a genome, against which single-nucleotide, phasing, and structural accuracy can be reliably assessed either locally or across the entire genome. In fact, a common way to unify complex variant representations has been to compare the underlying sequences themselves. ^21–23^ Genome benchmarking simply extends this idea to the whole genome and breaks the circular dependency of prior variant benchmarks.

The field’s reliance on variant benchmarking has been partly driven by the inability to sequence and assemble complete human genomes. However, the Telomere-to-Telomere Consortium recently overcame this barrier with the T2T-CHM13 assembly,^8^ completing the final 8% of the genome and revealing amplified gene arrays, ^8,24^ segmental duplications, ^25^ and genomic repeats ^26,27^ associated with critical genome functions. Recent developments streamlining the assembly of complete, diploid, T2T chromosomes ^28,29^ have resulted in additional T2T reference genomes for humans,^30–33^ apes, ^34,35^ and other vertebrates. ^36–38^ These technological advances enable the creation of comprehensive genome benchmarks for the thorough evaluation of new sequencing and analysis methods.

Here, we present results from the “Q100 Project.” The project’s name is meant to reflect our aspirational, yet practically unattainable, goal to reconstruct the complete, diploid genome of the widely used GIAB sample HG002 without errors (i.e., a Phred score of Q100, meaning an error rate of less than one per 10 Gb). Version 1.1 of the T2T-HG002 genome benchmark achieves T2T continuity for all 46 chromosomes, with only the interior of the rDNA arrays left unfinished; has been thoroughly validated using at least four different sequencing technologies; is free of detectable errors over 99.4% of the genome; and far exceeds both the completeness and accuracy of all prior benchmarks. In conjunction, we provide the Genome Quality Checker (GQC) software for benchmarking any type of sequence data against this new resource, including *de novo* genome assemblies, raw sequencing reads, phased variant calls, and pangenome-inferred haplotypes. These new resources remove the performance ceiling of past variant benchmarks, allowing all current and future technologies to be compared on equal footing and encouraging the development of improved methods for the sequencing of complete, personalized genomes.

## RESULTS

### A complete, nearly perfect assembly of the HG002 genome

The genome “HG002” is from the son of an Ashkenazi family trio (father-mother-son) originally sampled by the Personal Genome Project (PGP) ^39^ and now widely used as a sequencing technology benchmark (National Institute of Standards and Technology [NIST] Reference Material 8391, Coriell lymphoblastoid cell line (LCL) GM24385, PGP participant huAA53E0). The NIST has created a reference material derived from an expansion of the GM24385 cell line, showing a mostly diploid 46,XY karyotype (78% of sampled cells) with lower levels of tetraploidy (18%) and inversions (3%) (46,XY[57]/92,XXYY[13]/46,XY,?inv(3)(q 26.3q29)[3]) (STAR Methods; Figure S1). The T2T Consortium initially finished HG002’s X chromosome for comparison to CHM13^8^ and later added HG002’s Y chromosome to the T2T-CHM13v2.0 reference, which lacked its own ChrY ^24^ . HG002 was also used as a benchmarking genome for the Human Pangenome Reference Consortium (HPRC) ^40^ and for the development of automated T2T assembly methods.^28^

To complete the entire HG002 genome, we combined roughly 170× coverage of PacBio circular consensus (HiFi) and 209× of Oxford Nanopore Technologies (ONT) ultra-long reads from the HPRC ^41^ and GIAB Consortium ^42^ (Table S1). An initial Verkko ^28^ assembly was generated from these reads along with additional Illumina reads from HG002’s parents (HG003 and HG004) for phasing and Strand-seq ^43^ and Hi-C reads ^44,45^ for scaffolding across the rDNA arrays (STAR Methods). Validation of the rDNA scaffolding and estimation of the rDNA array sizes were guided by fluorescence *in situ* hybridization, ^46^ and rDNA consensus sequences were assembled by Ribotin. ^47^ All other gaps were closed by patching with alternate assemblies or by manual resolution of the assembly graph in a similar manner to CHM13. ^8^ T2T-HG002v0.7 was released to GitHub in November of 2022.

**Figure 2.**
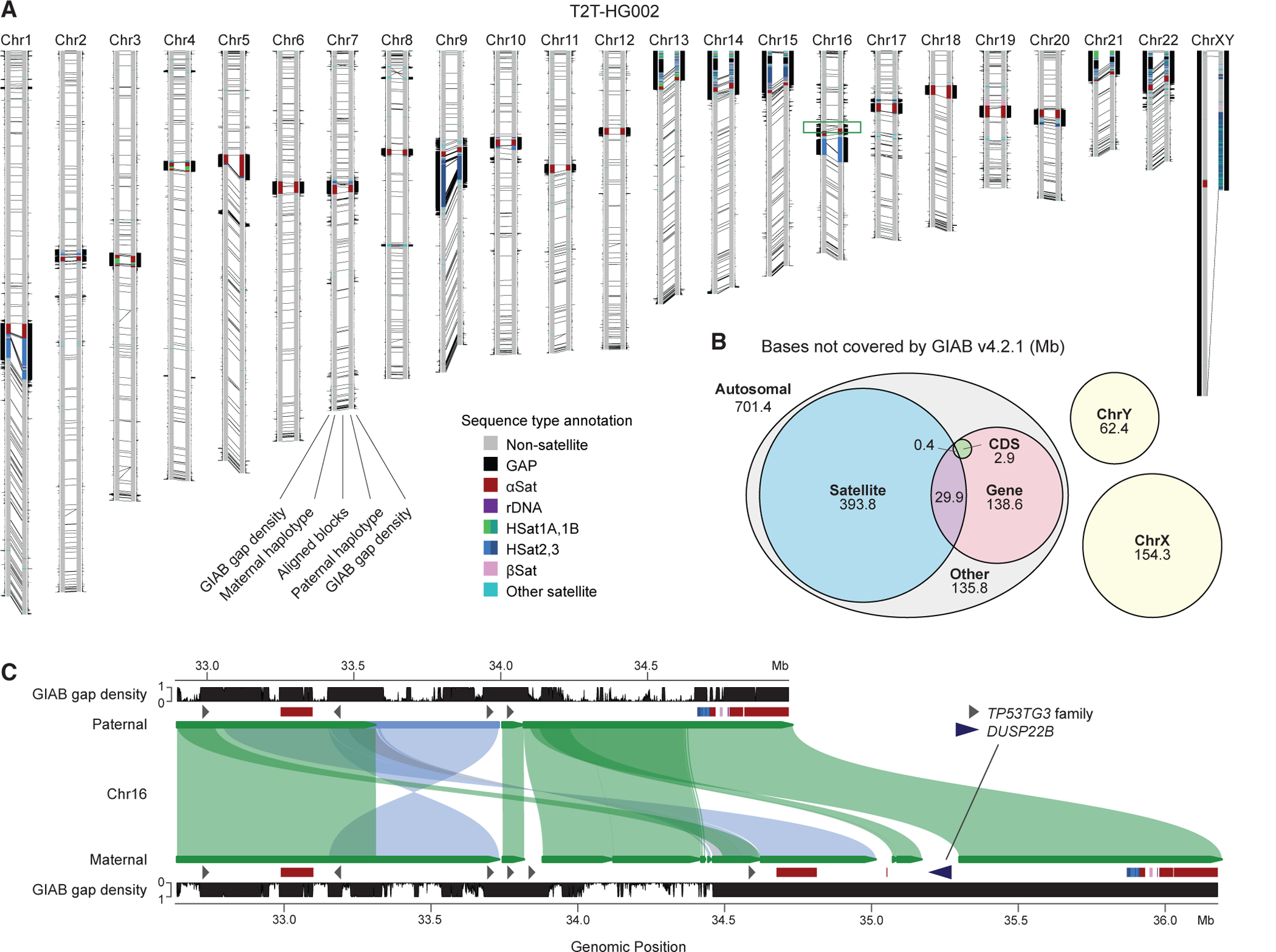
The complete diploid genome of HG002 (A) Genome ideogram of the T2T-HG002 assembly. Each homologous chromosome pair is displayed with its short arms at the top and the maternal copy to the left of the corresponding paternal copy. Satellite repeat annotations highlight the centromeres and other heterochromatic regions. Gray lines connecting the maternal and paternal chromosomes delineate the boundaries of aligned (homologous) blocks. On the outer sides of the haplotypes, density plots show the fraction of bases within 50 kb windows that are not included in the GIAB v4.2.1 variant benchmark “high-confidence regions” with larger values farther from the haplotype. A maximum value of one indicates a window that is entirely missing from the variant benchmark, such as within the centromeres or on the sex chromosomes, and a value of 0 indicates all bases in a window are covered by the benchmark. 99.7% of all windows genome-wide (119,674/120,013) include at least some bases not covered by the variant benchmark. (B) Breakdown by sequence class of the bases missing from the GIAB v4.2.1 variant benchmark that are covered by the genome benchmark. For the autosomal bases: satellite = all DNA satellite annotations. Gene = all annotated genic elements, including introns. CDS, coding sequence. The entire sequence of both sex chromosomes is missing. (C) Enlargement of the p-arm pericentromere of HG002 Chr16 showing the complex alignment of PAT and MAT haplotypes (forward: green, inverted: blue), satellite and protein-coding gene annotations, and gaps in the variant benchmark (same “GIAB gap density” plotted in A). The *DUSP22B* gene copy only appears on the MAT and is absent from GRCh38 but present in T2T-CHM13v2.0 (not shown), explaining why the variant benchmark does not cover this region. See also Figures S1–S5.

We iteratively polished, patched, and validated the T2T-HG002v0.7 assembly in three rounds using multiple independent short- and long-read datasets (STAR Methods). Due to the large quantity of available sequencing data for HG002, we adopted a custom polishing approach to better understand the nature of assembly errors, rather than using only automated methods. ^48,49^ During the first two rounds of polishing, crowdsourced curation of randomly selected examples of several large correction sets was tracked using issues in a public GitHub repository (Data and code availability), along with curation assignments and analysis of the reliability of different correction categories. Each correction set targeted particular technologies and/or modes of error (e.g., missing heterozygous sites callable with short reads), and those correction sets for which more than half of the curated corrections were judged to be correct were applied as a whole to the assembly (STAR Methods; Tables S2 and S3). After the first polishing round, version v0.9 of the assembly was released to GitHub in July of 2023; after a second round of polishing, v1.0.1 was submitted to NCBI in December of 2023 (GenBank: GCA_018852605.2, GCA_018852615.2); and, after a third round of polishing, v1.1 was submitted in July of 2024 (GenBank: GCA_018852605.3, GCA_018852615.3). In total, the three rounds of polishing resulted in 38,037 small corrections made to the assembly, including 5,420 single-base substitutions, 26,779 small deletions (50 or fewer base pairs), and 5,628 small insertions (Figure S2), along with 210 larger consensus patches of regions totaling 14,182,804 base pairs. Analysis of Strand-seq ^50,51^ reads confirmed the overall structural correctness of the v1.1 assembly, showing no evidence of inversion errors (STAR Methods; Figure S3). Unless otherwise stated, the rest of the manuscript concerns version T2T-HG002v1.1, or T2T-HG002 for brevity (Figure 2A).

We estimated the Phred-scaled consensus quality (QV) and phasing accuracy of the assembly with Merqury ^52^ using a database of PacBio HiFi and Element 31-mers from HG002 and Illumina parental 31-mers from HG003 and HG004. After polishing, QV increased from Q63.1 for the v0.7 assembly to Q68.9 for the v1.1 assembly (Q76.2 using 21-mers for comparison to other studies; note that smaller *k*-mers are less likely to be unique and therefore undercount assembly errors within repeats, so we prefer to report 31-mer statistics) (Tables S4 and S5). This equates to reducing the estimated error rate from roughly one per two million bases to one per eight million bases, which is substantially lower than all prior HG002 assemblies tested ^10,53,54^ (Table S4). Interestingly, we noted that certain correction sets resulted in little-to-no QV improvement, due to the limitations of *k-*mer-based methods at such low error rates. This included corrections that restored heterozygous variants or were made within long homopolymers, tandem repeats, and repetitive sequences, where the *k*-mer-based methods are unable to detect errors. Additionally, in evaluating the assembly for phasing error, Merqury flagged large blocks of the immunoglobulin gene loci as haplotype switches. We confirmed that rather than reflecting errors in the benchmark, these inconsistencies between parental *k-*mer composition and the assembly’s haplotypes were caused by V(D)J recombination in the LCLs, ^55^ making the parental-specific markers unreliable for evaluating these regions. V(D)J recombination was confirmed in the NIST reference material by comparison to short reads from a peripheral blood mononuclear cell-derived induced pluripotent stem cell (iPSC) line of the same individual (Coriell GM27730). Consequently, the immunoglobulin loci were manually validated without the use of parental *k*-mers.

Altogether, 99.35% of the diploid genome length is free of any detectable errors in T2T-HG002v1.1. The remaining 3,172 low-confidence regions cover 38.8 Mb (0.65%) of the 6.0 Gb assembly (Tables S6 and S7). The majority (86.3%) of these low-confidence bases are composed of rDNA sequence arrays, which were flagged in their entirety as low-confidence. These arrays include nine gaps (Ns) spanning 31.1 Mb, a resolved rDNA array on the paternal copy of Chr13 spanning 275.9 kb, and 2.4 Mb of rDNA sequence flanking the gaps (Figure S4). The remaining 5.3 Mb of low-confidence sequence (0.09% of the assembly) is enriched for satellites and segmental duplications and was flagged by either base-level or structural evaluation, erring on the side of caution to ensure the integrity of the benchmark. Included in these flagged regions are 293 regions (183.1 kb) that contain 31-mers not found in either PacBio HiFi Revio SPRQ or Element UltraQ reads, as well as 794 possible haplotype switch errors (31.6 kb) indicated by 31-mers from parental Illumina reads. Mapped PacBio and ONT reads flagged 237 regions (4.9 Mb) with excessive secondary alleles present in the alignments of both technologies, indicative of a possible structural or consensus issue.^56^ Lastly, 1,893 regions (486.6 kb) were flagged by DeepTrio ^57^ using Element reads, 410 (82.9 kb) by DeepPolisher ^48^ and DeepVariant, ^57^ 19 (3.8 kb) by Sniffles, ^58^ 20 (4.5 kb) via contributed GitHub issue submissions, and 3 (44.7 kb) by T2T-Polish. ^59^ These low-confidence regions are excluded from the v1.1 benchmark, and their future validation and/or correction will be tracked in the HG002 GitHub issues repository.

Lastly, the assembly was compared with GIAB’s most recent (v4.2.1) HG002 variant benchmark. In addition to the sex chromosomes, T2T-HG002 includes an additional 701.4 Mb (11.7%) of high-confidence autosomal sequence that is absent from the v4.2.1 variant benchmark, mostly covering satellite repeats but also hundreds of Mb of non-satellite, typically segmentally duplicated, sequence (Figure 2B). In regions reliably covered by both benchmarks (covering approximately 2.66 Gb of the variant benchmark regions), polishing from v0.7 to v1.1 reduced the number of discrepancies between the genome and variant benchmarks from 5,972 to 219 variants (Table S8). Nearly all of the remaining discrepancies were found to be GIAB v4.2.1 errors in complex variants or difficult-to-map regions. Manual curation of 20 randomly selected variants outside of segmental duplications indicated only two errors and one possible mosaic variant in the T2T-HG002v1.1 assembly, all within homopolymers and dinucleotide tandem repeats. Within segmental duplications (covering 81 Mb), 1,015 differences remain, but inspection of 20 randomly selected variants suggests these should have been excluded from the GIAB v4.2.1 benchmark due to reference bias. All were within regions that could not be reliably mapped between HG002 and GRCh38 due to repeats, structural variation, or gene conversion. An example of such a region is shown in Figure 2C, where the maternal Chr16 pericentromere contains an insertion of *DUSP22* that does not exist on the paternal haplotype (PAT) or GRCh38, both of which only have *DUSP22* copies near the p-arm telomere of Chr6. As such, this region of the maternal haplotype (MAT) is entirely absent from prior HG002 variant benchmarks, despite *DUSP22* being a known human-specific duplicated gene family associated with several cancers.^60,61^

### Personalized genome annotation

We generated gene annotations for each haplotype of the T2T-HG002 assembly using Liftoff ^62^ to map genes from T2T-CHM13v2.0 (see STAR Methods). In addition, we used LiftOn ^63^ and miniprot ^64^ to annotate additional gene copies based on protein-to-genome alignment of translated MANE (v1.4) transcripts (a high-quality annotation in which a single representative transcript is selected for each protein-coding gene). Mitochondrial and immunoglobulin genes were excluded from the annotation totals described below. Mitochondrial genes are relatively conserved across individuals, while immunoglobulin genes are difficult to annotate based solely on sequence similarity due to their high variability.

The final annotation includes 59,323 genes on MAT, including ChrX, and 57,741 genes on PAT, including ChrY (Table 1). We subdivided the total gene count into three main categories: coding, non-coding, and pseudogenes. Coding genes produce mRNAs that encode proteins, whereas non-coding genes include functional RNA molecules such as long non-coding RNAs (lncRNAs), microRNAs (miRNAs), and others. Pseudogenes are degenerate copies of coding genes that are assumed to be non-functional, evidenced by substantial divergence and loss of function variants compared with their known coding paralogs. Liftoff and LiftOn transfer gene type labels directly from the source annotation (T2T-CHM13v2.0), and we employed those same labels for our downstream analysis of genes in HG002.

Because the CHM13 assembly is haploid and contains both sex chromosomes, we combined the HG002 MAT with ChrY for comparison. The HG002 annotation of MAT plus ChrY contains 60,017 annotated genes compared with 59,763 for CHM13, an increase of 254 (not including rRNAs). Only 39 distinct genes (also excluding rRNAs) were absent from both T2T-HG002 haplotypes relative to T2T-CHM13 (STAR Methods; Table S9), with all three missing protein-coding genes (*HLA-DRB5*, *CT45A8*, and *OPN1MW*) known to naturally vary in copy number. For example, HG002 contains only one copy of the X-linked *OPN1MW/OPN1MW2/OPN1MW3* opsin gene family, ^65^ which Liftoff annotated as *OPN1MW3*.

The gene counts presented in Table 1 exclude all rRNA genes due to the remaining rDNA gaps in the HG002 assembly. The v1.1 assembly contains 49 individual rDNA copies in scaffolds, and 100 Ribotin morphs (i.e., consensus sequences for distinct rDNA units) are made available separately as unplaced contigs. Prior work estimates over 600 total rDNA copies in the HG002 genome, ^46^ with approximately 430 on MAT and 197 on PAT, which is substantially higher than the ∼400 copies in CHM13. In contrast to the 45S rDNA arrays on the acrocentric chromosomes, both 5S arrays on Chr1 are complete in HG002, comprising 91 MAT copies and 63 PAT copies, compared with 99 in CHM13.

The complete, annotated diploid assembly of HG002 allowed us to compare the total gene content between MATs and PATs of the same individual. The primary questions we addressed were (1) how many protein-coding genes are “broken” in each haplotype and (2) how many protein-coding genes have different copy numbers between the two haplotypes? We focused our analysis of broken genes on those contained in MANE. This restricted our analysis to 18,976 genes on MAT and 18,231 on PAT. A protein-coding gene was defined as broken if the mapped MANE transcript met at least one of the following criteria: (1) an invalid start and/or stop codon, (2) a premature in-frame stop codon, or (3) transcripts whose protein translation was less than 80% identical to the corresponding MANE protein. Using these criteria, we identified 129 broken protein-coding genes on MAT and 121 on PAT (STAR Methods; Table S10). Broken genes were enriched within segmental duplications, with 28% (69/250) intersecting an annotated segmental duplication, compared with just 8% (2,996/36,957) of the unbroken genes. One such example, located within a segmentally duplicated region of Chr15, is the human-specific fusion gene *CHRFAM7A*, ^66^ which is a dominant negative regulator of α7 neuronal nicotinic acetylcholine receptor (*CHRNA7*) function. ^67^ HG002 PAT contains a common 2 bp frame-shifting deletion in exon 6 that is associated with increased risk of various neuropsychiatric disorders, but the recently duplicated, polymorphic nature of this locus complicates analysis. ^68^ Contrary to previous literature that suggests the 2 bp deletion allele is typically inverted relative to the wild-type, ^69^ both HG002 MAT (wild-type) and PAT (Δ2 bp) copies are in the same orientation as the GRCh38 and CHM13 references.

Next, we computed gene copy numbers across both T2T-HG002 haplotypes (STAR Methods; Table S11) and identified haplotype-specific genes (i.e., those unique to only one haplotype). After filtering to reduce false positives (FPs) from closely related genes and excluding sex chromosomes, we identified 13 MAT-only and 12 PAT-only autosomal genes (STAR Methods; Table S12). Our filtered set of haplotype-specific genes includes several well-characterized examples of copy-number variable genes, such as *DUSP22* (Figure 2C), as well as *CFHR1*, *CFHR3*, *GSTT1*, and *GSTM1*. HG002 PAT includes a co-deletion of *CFHR1* and *CFHR3*, which is a fairly common haplotype (20% of chromosomes in a UK-based cohort) shown to protect against age-related macular degeneration. ^70^ The glutathione S-transferase genes *GSTM1* and *GSTT1* are another pair of copy-number variable genes in HG002 that play a role in the neutralization of toxic compounds, with homozygous deletions (null alleles) being linked to an increased risk of cancer in certain populations. ^71,72^ HG002 is heterozygous for both, containing a single copy of *GSTM1* only on PAT and a single copy of *GSTT1* only on MAT. Excluding genes that were entirely absent from one haplotype, we identified 57 genes with a higher copy number on the MAT haplotype and 39 with a higher copy number on PAT. These differences reflect true variation and highlight the value of a haplotype-resolved assembly in structurally variable regions of the genome.

To enable easy access and exploration, both haplotypes of T2T-HG002v1.1 have been organized along with additional data tracks in a UCSC Genome Browser hub. ^73^ In addition to the diploid gene annotation, we generated sequence-based annotation tracks for GC percent, CpG islands, repetitive elements, segmental duplications, subtelomere repeats, and centromeric satellites. Validation tracks provide the locations of all known assembly issues, depth of coverage for mapped HiFi and ONT reads, and secondary variants reported by NucFreq. ^56^ Whole-genome alignments are also included, showing the position of all heterozygous sites and enabling quick locus switching and lift over between HG002 haplotypes, T2T-CHM13, and GRCh38. We also provide multiple tracks derived from HG002 long-read functional genomics experiments, including 5-methylcytosine (5mC) predictions from both HiFi and ONT sequencing, chromatin accessibility and regulatory element predictions from Fiber-seq, ^74^ and bulk RNA and single-cell transcriptome data from PacBio Iso-Seq Kinnex and MAS-Seq. Uniquely, because this data is based on long-read sequencing and drawn from matched HG002 cells, it can be confidently mapped genome-wide, providing unprecedented coverage of the epigenome and transcriptome across both haplotypes.

Because HG002 cells are openly available for sequencing and research (including iPSC lines), this comprehensive browser resource presents a unique resource for personalized genomics (Figure S5). Two companion studies demonstrate the new capabilities enabled by a personalized reference genome: Tullius et al.^75^ apply Fiber-seq to HG002 sperm and lymphoblastoid cells to evaluate the nucleosome-to-protamine transition genome-wide, uncovering epigenetic features governing the intergenerational inheritance of the centromere kinetochore binding region as well as traces of regulatory information retained in the sperm epigenome, while Xu et al. (appear to) profile the chromatin architecture of HG002 centromeric satellites, revealing coordinated relationships between mCpG, H3K9me3, and CENP-A occupancy across centromere regions, as well as their epigenetic transmission through cell passaging. ^76^ Such analyses would be impossible using GRCh38, which is an incomplete mosaic reference not linked to any one biological sample.

### Benchmarking genome assemblies

*De novo* assemblies of diploid human genomes using PacBio HiFi, ONT ultra-long, and Hi-C sequencing reads have begun to yield phased, chromosome-scale scaffolds of complete haplotypes. ^28,29,77^ Additionally, the development of pangenome databases ^10^ has enabled genome inference methods that can predict personalized, haplotypic sequences even from short reads. ^78^ However, both *de novo* and inferred assemblies typically contain numerous structural errors like repeat expansions or collapses, misjoins, inversions, haplotype switches, and smaller substitution or insertion/deletion (indel) errors. ^79^ Since the T2T-HG002 assembly is complete and achieves nearly perfect accuracy, it can be used as a genome benchmark to identify likely errors in test assemblies generated by different sequencing and assembly approaches. ^80,81^

Errors in alternative assemblies of HG002 can be directly identified by aligning each haplotype to its corresponding location in the genome benchmark and cataloging the differences. However, previously developed tools for assembly quality assessment (e.g., QUAST ^82^ and GenomeQC ^83^ ) do not consider the case of a diploid reference genome, and others (e.g., CRAQ ^84^ ) only evaluate read alignments and not how well an assembly reproduces a genome sequence. We developed the GQC software to enable comprehensive evaluation of DNA sequences against a highly accurate, diploid genome benchmark. GQC implements a haplotype-aware alignment strategy to collect statistics on continuity, accuracy, and phase consistency. Briefly, haplotype-specific *k*-mers are extracted from the diploid benchmark genome, and a two-state hidden Markov model (HMM) over these markers is used to determine haplotype blocks in the test assembly (STAR Methods). This information is used to refine the whole-genome alignments and differentiate phasing errors from base-calling errors, which is especially helpful when evaluating pseudohaplotypes (i.e., assembled contigs that have not been fully phased). Because every error is assigned a position on the reference genome, all analyses can be stratified by a list of genomic regions to include or exclude.

Using GQC, we evaluated five previous assemblies of HG002 spanning 5 years of technology improvement against the T2T-HG002 genome benchmark (Tables S13 and S14): the “Ash1v2” haploid reference assembly from 2020,^53^ a diploid assembly from the initial “Hifiasm” publication in 2021, ^54^ a diploid assembly from the “HPRCv1” pangenome release in 2022, ^10^ a diploid assembly from the initial “Verkko” publication in 2023, ^28^ and a diploid “ONT LC24” assembly released by ONT at the London Calling conference in 2024 (https://epi2me.nanoporetech.com/lc2024_t2t/). The Jarvis et al. assembly ^40^ represents an early iteration of the HPRCv1 assembly with slightly worse accuracy and so is included only in the supplementary tables.

**Figure 3.**
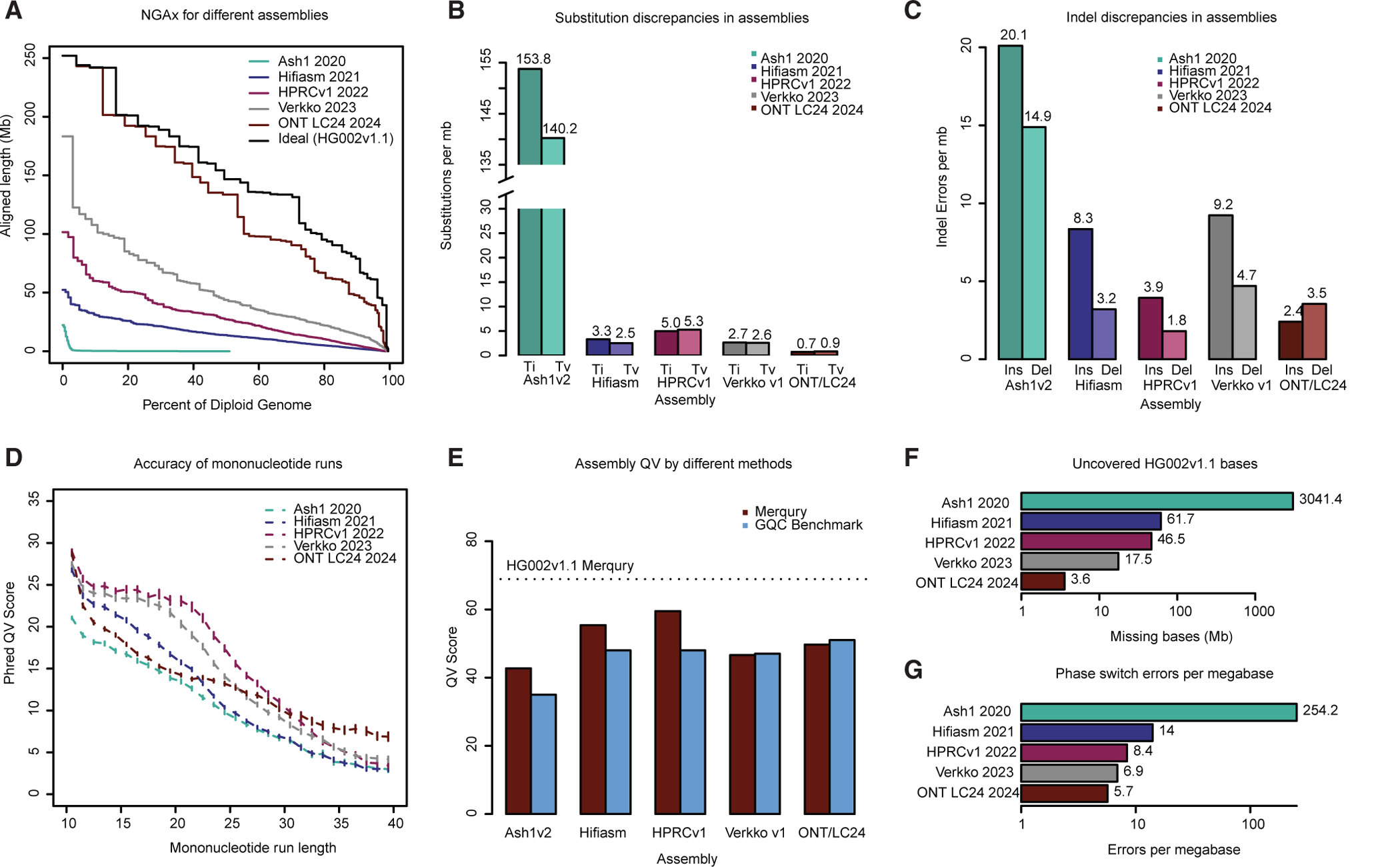
Five assemblies evaluated against the genome benchmark (A) NGAx plot calculated from the lengths of uninterrupted alignments to the T2T-HG002 benchmark. Alignments were broken at locations of indels that were at least 10 kb in length. (B) Within-alignment substitution rates plotted with transition rates (Ti) to the left and transversion rates (Tv) to the right. Substitution alleles that match the alternate haplotype at heterozygous sites are not included in these counts but are instead classified as phase switch errors. (C) Within-alignment indel rates, plotted with insertion rates (Ins) to the left and deletion rates (Del) to the right. Alleles that match the alternate haplotype at heterozygous sites are not included. (D) Mononucleotide run accuracy is measured by alignments to mononucleotide runs of different lengths in the benchmark and reported as a Phred-scaled quality score: − 10*log_10_ (#erroneous runs/#aligned runs). For all homopolymer run length bins, error bars for the plotted Phred QV scores were calculated using the R binconf package to calculate Bayesian confidence intervals for homopolymer accuracy rates using the Wilson method, and the resulting limits were converted to minimum and maximum Phred QV scores for the intervals. (E) Phred-scaled quality scores calculated using *k*-mer-based (Merqury) and alignment-based (GQC) methods: − 10*log_10_ (#discrepancies/#aligned bases). (F) Number of non-N bases in the genome benchmark uncovered by primary alignments of the test assemblies to the benchmark. (G) GQC phase switch rates within alignments of the test assemblies to the benchmark. See also Figure S6.

Across several measures of completeness and accuracy, the assemblies showed progressive improvement over time (STAR Methods). Specifically, NGAx plots show steady increases in the lengths of long scaffolds that are continuously alignable to the genome benchmark (Figure 3A). NGAx is especially powerful in conjunction with an accurate reference since it plots the size-ordered lengths of assembly-to-benchmark aligned blocks versus the percent of the benchmark covered and, therefore, reflects both the continuity and structural accuracy of the test assembly.^81^ Additionally, rates of substitution errors within alignable sequences have fallen over time by more than two orders of magnitude, and indel rates have fallen roughly tenfold (Figures 3B and 3C). There have been more modest improvements in the accuracy of mononucleotide run lengths (Figure 3D), with all assemblies continuing to struggle with long homopolymers, especially those greater than 20 base pairs in length.

In addition to structural validation, our genome benchmark also measures the precise base accuracy of an assembly without the limitations of *k*-mer-based approaches such as Merqury. These methods are typically based on the detection of “error *k*-mers,” i.e., short sequences that are present only in the tested assembly and not in the raw sequencing data. Comparing base quality measured by GQC and Merqury across the same five assemblies revealed several interesting trends (Figure 3E). Using a combined database of 31-mers derived from the HiFi and Element reads, Merqury tended to overestimate the quality of the PacBio assemblies compared with GQC (Δ Ash1+7.9QV, Hifiasm+7.9QV, and HPRC+11.7QV), likely due to a confirmation bias toward the PacBio data and a failure to penalize missing or collapsed sequences. Conversely, assemblies that included ONT data showed a reduced Merqury quality score but an improved GQC score (Δ Verkko-0.4QV, LC-1.4QV), likely due to regions of the genome recovered only by the ONT data and possibly underrepresented in the HiFi and Element reads. This conclusion is supported by the increased completeness of the ONT-based assemblies versus the PacBio assemblies, which suffer from intermittent coverage dropout in GA-rich regions^85^ (Figure 3F). Lastly, phase switch errors, as identified by GQC’s HMM method, have also steadily decreased, with the ONT-including assemblies showing the best performance due to their increased read lengths (Figure 3G). These results highlight the advantages of a complete genome benchmark and suggest caution when interpreting *k*-mer-based quality scores alone.

### Benchmarking sequencing reads

Recent improvements in genome assembly quality are largely a consequence of ongoing improvements to the length and accuracy of sequencing reads. However, it is not sufficient to rely on the quality values reported by sequencing instruments themselves. Even if reported quality values could be assumed to be accurate, they do not capture other important quality information such as coverage biases (e.g., GC-rich or GA-rich sequences), error biases (e.g., difficulty with homopolymer runs), rates of chimeric reads, or the frequency of different types of errors (e.g., insertions, deletions, or substitutions). A genome benchmark enables us to comprehensively evaluate sequence reads by comparing them directly to the genome from which they originated and ensures that quality is measured across a broad range of metrics and sequence contexts.

Given BAM-formatted reads aligned to their genome of origin, GQC reports total aligned and clipped bases in the reads, as well as rates per megabase of read substitution and indel error within the primary alignments. It further stratifies error rates in homopolymer, dinucleotide, trinucleotide, and tetranucleotide runs of different lengths. Substitution error rates are reported for each of the twelve possible single-base errors, and if base quality scores are included in the BAM file, tallies of substitution and indel errors are reported for each quality score value.

**Figure 4.**
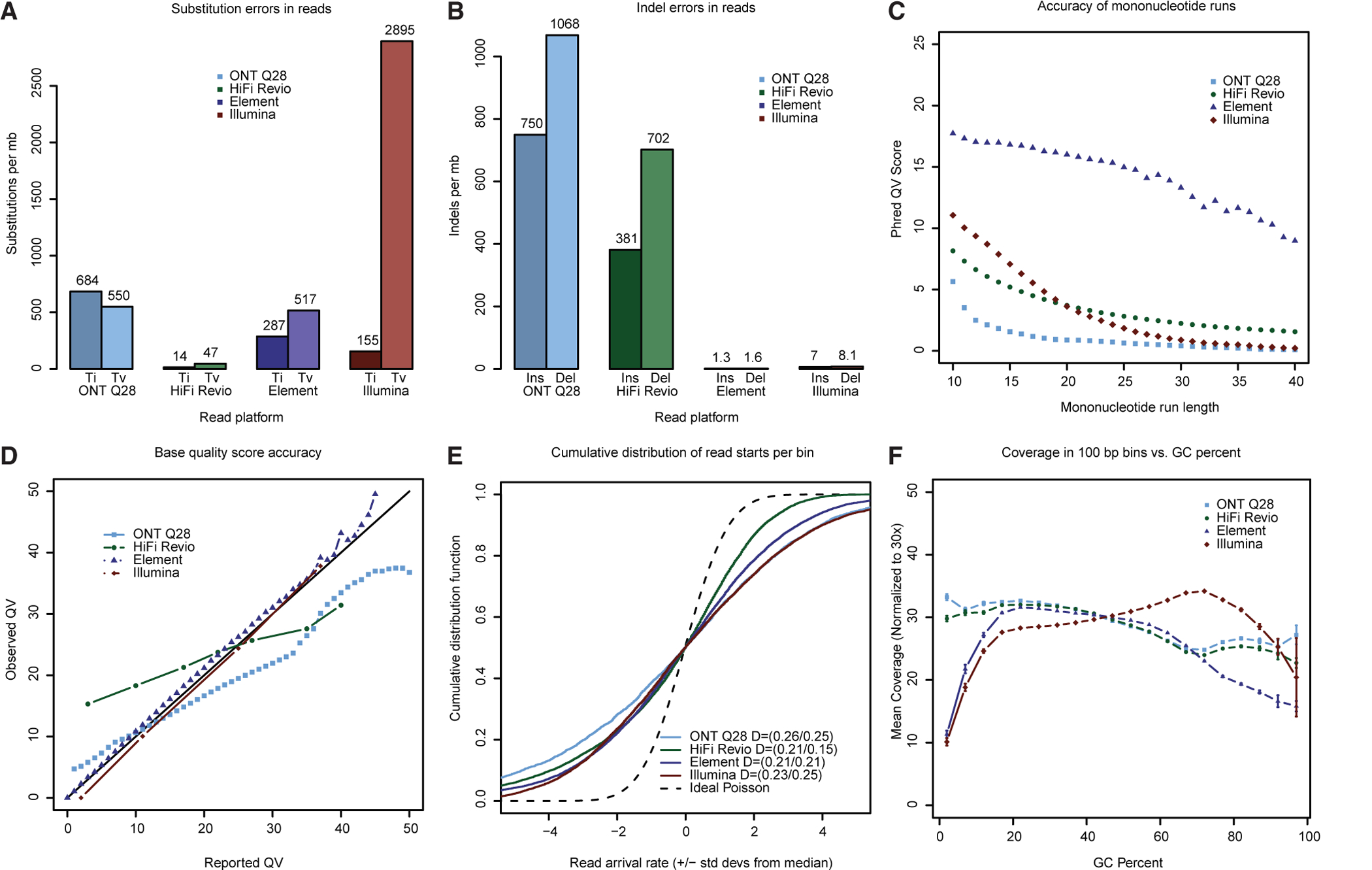
Four sequencing technologies evaluated against the genome benchmark (A) Read substitution rates within alignments of reads to the genome benchmark. “Ti” is the count of transitions, and “TV” is the count of transversions. (B) Insertion and deletion rates within alignments of reads to the genome benchmark. (C) Phred-scaled quality scores for homopolymer runs of different lengths. To be considered correct, a homopolymer must be the correct length and free of single-nucleotide errors both within the repeat and in the 5 flanking bases before and after the repeat. For all homopolymer run length bins, error bars for plotted Phred QV scores were calculated by using the R binconf package to calculate Bayesian confidence intervals for accuracy rates using the Wilson method, and then the limits were converted to minimum and maximum Phred QV scores for those intervals. In this case, all calculated intervals were smaller than the size of the plotted points and therefore are not visible in the plot. (D) Accuracy of base quality scores calculated from actual alignment match rates (observed) versus base quality scores reported with the reads (reported), including both substitutions and indels. (E) Cumulative distribution of read starts per bin (arrival rate) for different technologies scaled by the expected standard deviation for a Poisson distribution and shifted to put the median at zero for visualization. Kolmogorov-Smirnov distances (D values) correspond to the maximum difference between a curve and the ideal below and above the median, respectively. (F) Mean coverage in 100 bp windows as a function of %GC. Vertical lines show the standard error of the mean for windows with each percentage, extending from minimum values of the mean minus the standard error to maximum values of the mean plus the standard error. See also Figure S7.

Comparing four recent datasets with GQC shows marked differences in base-calling performance and coverage uniformity (ONT “Q28” Ultra-Long Sequencing Kit V14, PacBio Revio HiFi ICSv13 with DeepConsensus v1.2, Element 2×150 Aviti “Q50” UltraQ from 400 bp inserts, and Illumina 2×151 NovaSeq PCR-Free from 300 bp inserts) (Tables S13 and S15). Substitution error rates show that Illumina short reads have five times the rate of transversion errors as the other platforms ^86^ (Figure 4A), while HiFi and ONT long reads have indel error rates two orders of magnitude higher than the short reads (Figure 4B). For homopolymers, Element reads show an order of magnitude lower error rate than all other platforms (Figure 4C). It is also possible to compare observed versus reported quality values for each technology by binning their bases by quality score and calculating error rates from the alignments. While short read (Illumina, Element) base quality scores are well calibrated, base calls in the long reads (HiFi, ONT) show lower-than-reported accuracy, on average, for bases with reported quality scores greater than Q20 (Figure 4D). However, standard basecalling formats do not include a separate probability for indels, leading to ambiguity in their representation, which particularly affects the long-read platforms. The QV correlation is better for long reads when considering substitution errors alone, especially for ONT, whereas HiFi basecalls tend to overestimate the rate of substitution error (Figure S7). Analysis of the distribution of binned coverage shows that all technologies show a wider distribution than the expected Poisson (Figure 4E) and that while HiFi Revio is more overdispersed for coverage values lower than the median, Illumina PCR-Free is more overdispersed for higher coverage values. GQC can also report windowed read coverage as a function of GC content, which shows the long reads outperforming short reads for high %AT and all technologies struggling with high %GC (Figure 4F).

### Benchmarking variant call sets

Variant call sets are by far the most commonly benchmarked genomic data type but do not typically assess the entire genome. Conveniently, it is possible to convert variant sets to genomes and vice versa to better understand their limitations. Tools such as dipcall ^15^ and PAV ^87^ report variants from the alignment of a diploid assembly to a reference, which can then be compared with a variant benchmark to measure assembly consensus accuracy. ^88^ Conversely, it is possible to apply a set of variants to a reference genome using BCFtools ^89^ and, in the process, build a “variant-constructed genome” by updating a reference genome sequence with the alleles reported in a VCF file. Ideally, a perfect variant set would produce a variant-constructed genome that perfectly matches the sample being sequenced, but in practice, the output remains limited due to the incompleteness of the reference and/or variant calling.

To measure how much of the genome is being overlooked by state-of-the-art variant calling approaches, we generated multiple HG002 variant-constructed genomes against both GRCh38 and T2T-CHM13 references. Since a genome benchmark consists of complete haplotypes, the variant callsets must be at least partially phased for evaluation, and it is necessary to know which regions of the reference genome have been sufficiently covered by the sequencing data, as provided by the gVCF format.^90^ Using the best-performing single-technology approach,^3^ we used variants called on HiFi long reads with DeepVariant^57^ and phased with HapCut2^91^ using the same data. We then generated diploid variant-constructed genomes by applying the called variants from each haplotype to the reference and masking bases that were below genotype quality (GQ) score thresholds of 10, 20, 30, and 40 (STAR Methods). This resulted in eight constructed genomes (two references × four GQ thresholds), which we evaluated by GQC to assess quality and completeness (Table S14).

The variant-constructed genomes built using GRCh38 cover 93.8%–92.9% of the HG002 genome (min GQ 10–40). By contrast, those built using CHM13 cover 97.8%–95.7% (Figure S8), demonstrating the improved coverage of variant call sets produced by mapping against a complete reference sequence. However, many of these added bases are in difficult-to-call regions of the genome, such as satellite repeats, and the average quality of the consensus sequence suffers slightly as a result. Average consensus QV (− 10 *log_10_ ((substitutions + indels)/total_aligned_bases), where “total_aligned_bases” refers to the number of aligned benchmark bases that are not excluded from GQC’s evaluation) ranged from 37 to 40 for the GRCh38-variant-constructed genomes (min GQ 10–40) and from 35 to 39 for the CHM13-variant-constructed genomes. In particular, there was a higher rate of single-nucleotide substitution errors (mainly confined to the satellite repeats) when using the CHM13 reference compared with GRCh38 (72.3/Mb versus 41.1/Mb for GQ ≥ 40), while the indel rate was comparable (52.6/Mb versus 50.6/Mb, respectively) (Figure S8). When restricted to only parts of the HG002 genome covered by variant-constructed genomes from both references (thus, eliminating most satellites), average QV ranged from 37 to 41 for the GRCh38-variant-constructed genome and from 38 to 41 for the CHM13-variant-constructed genome (Table S14). In comparison, the best-performing historical HG002 assembly evaluated here, ONT LC24, achieves a QV of 51 with coverage 99.94%. Thus, when averaged across the whole genome, *de novo* assembly can now outperform variant calling accuracy by an order of magnitude and achieve higher genome coverage than mapping to T2T-CHM13.

### Relationship between genome and variant benchmarking

Due to their increased coverage and more natural representation of complex genomic regions, genome benchmarks can also be used to improve variant benchmarks. The first T2T assembly of HG002’s X and Y chromosomes ^24^ was used to create phased variant benchmarks by aligning it against GRCh37, GRCh38, and T2T-CHM13v2.0 references to call variants in complex regions and exclude regions of ambiguous orthology. ^16^ The resulting updated GIAB variant benchmarks are more accurate and comprehensive, enabling the evaluation of sex chromosomes, complex structural variants, large indels, indels within long homopolymers and tandem repeats, and variants within closely related segmental duplications for the first time.

To assess what types of errors may be missed by variant benchmarks and facilitate a transition from variant-centric to genomecentric analysis, we analyzed the relationship between genome benchmarking metrics (e.g., consensus and phasing accuracy) and standard variant benchmarking metrics (e.g., precision, recall, F1, FPs, false negatives [FNs], and genotype errors). As a case study, we evaluated the previous Jarvis et al. diploid assembly of HG002^40^ using both genome and variant benchmarking and compared the errors identified (Table S16). To focus our comparison on differences between the two approaches, rather than the underlying benchmarks, we used an updated version of the GIAB variant benchmark that was created by aligning the T2T-HG002 assembly to GRCh38 (https://ftp-trace.ncbi.nlm.nih.gov/giab/ftp/data/AshkenazimTrio/analysis/NIST_HG002_DraftBenchmark_defrabbV0.019-20241113/). Single-nucleotide variant (SNV) and indel designations for genome benchmarking were based on comparison of the HPRCv1 assembly directly to T2T-HG002, while variant benchmarking was based on alignment of HPRCv1 to GRCh38 and comparison to the variant benchmark.

**Figure 5.**
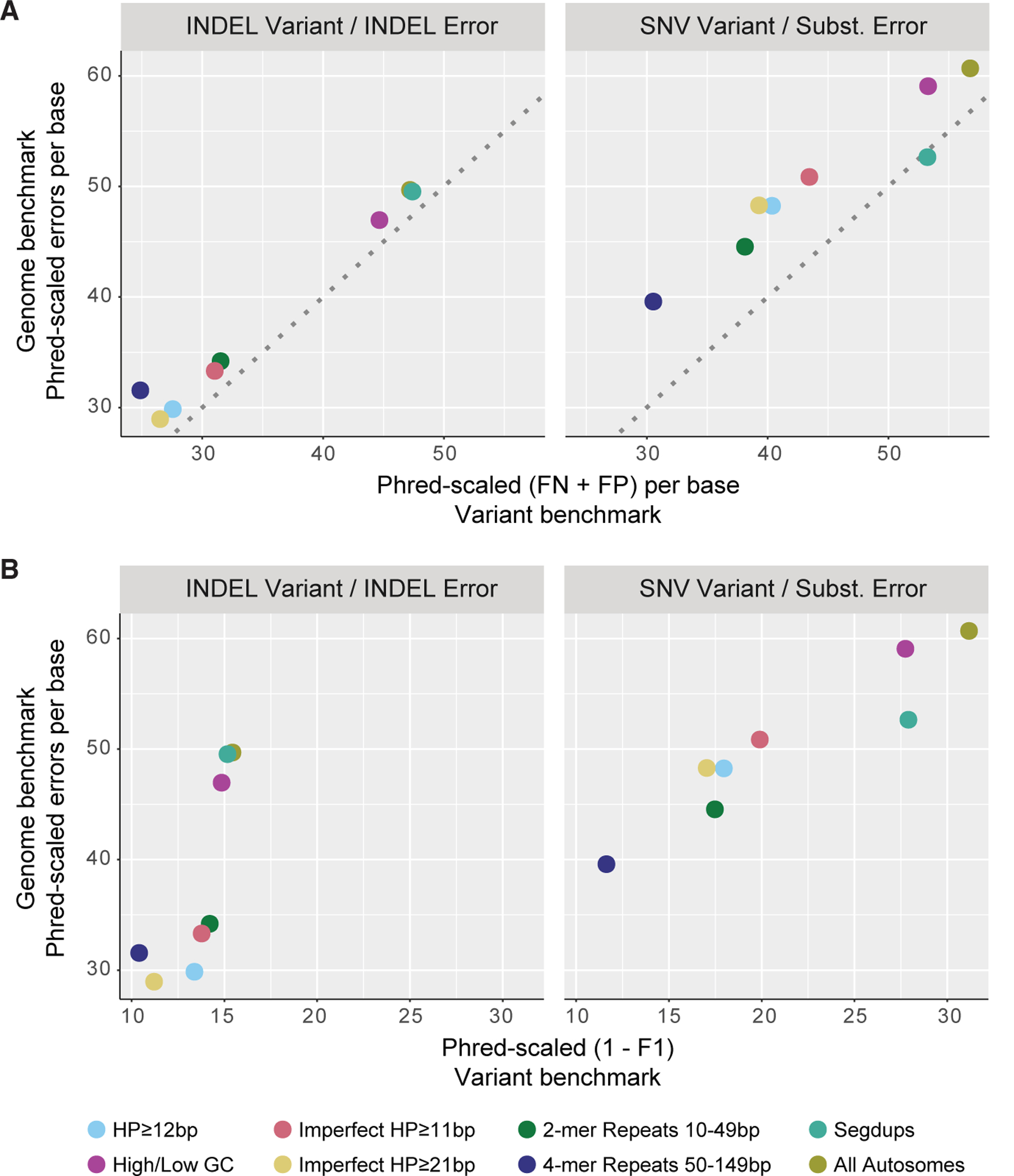
Comparison between variant and genome benchmarking results Indel and SNV errors were identified in the Jarvis et al. assembly of HG002 using both the GIAB HG002 variant benchmark and T2T-HG002 genome benchmark, with results stratified by various sequence contexts (e.g., light blue = homopolymers ≥12 bp). For comparison, genome benchmarking errors were restricted to those that could be successfully projected onto GRCh38 GIAB small variant confident regions (30,505 of 66,544 genome benchmarking errors). (A) SNV and indel variant benchmarking FNs and FPs per base of GIAB confident regions versus substitution and indel genome benchmarking errors per base in the Jarvis et al. assembly (normalized by the number of bases in each stratification). Error rates are plotted as Phred-scaled quality: − 10*log_10_(Perror). Points above the dotted line indicate contexts for which variant benchmarking reported more errors than genome benchmarking. (B) Variant benchmarking F1 score versus genome benchmarking errors per base. F1 scores are not directly relatable to per-base error rates because they are a fraction of true variants rather than a fraction of bases, but the Phred-scaled (1-F1) is shown on the same log scale to depict the relationship of these commonly used performance metrics. Individual plots of FP, FN, precision, and recall are shown in Figure S9. See also Figures S8–S13.

Genome benchmarking identified 66,544 errors in the HPRCv1 assembly, of which 50,843 could be lifted to GRCh38 and 30,505 fell within the small variant benchmark’s high-confidence regions. Thus, more than half of the total genome benchmarking errors in the HPRCv1 assembly were not assessed by variant benchmarking due to reference bias and a lack of benchmark coverage. Restricting the comparison to only the high-confidence regions, FP and FN rates from variant benchmarking largely correlated with error rates from genome benchmarking across various repeat stratifications (Figures 5A and S9). For these regions, variant benchmarking reported more errors (sum of FP and FN) than genome benchmarking, except for SNVs within segmental duplications, where the two methods reported a similar frequency of error. We found that each genome benchmarking error often translated into multiple variant benchmarking errors due to complex variants in homopolymers and tandem repeats or differences in how phasing errors were reported, which explained the higher rate of errors from variant benchmarking (STAR Methods; Figures S9–S11). In this way, genome benchmarking provides a more straightforward interpretation of error without the added complexity of natural variation between the sample and reference. We found that phase switch errors (swapped haplotypes) and haplotype collapse errors (loss of heterozygosity) were often more interpretable in the variant benchmarking results, but this limitation could be addressed by future genome benchmark tooling.

Genome benchmarking error (or consensus accuracy) is not directly comparable to more commonly used benchmarking metrics like precision and recall, because these metrics use different denominators. Whereas genome benchmarking measures accuracy at every base in the genome, precision/recall only measures known variant sites. For example, variant-based indel F1 scores do not necessarily correlate with genome benchmarking error rates because most true indels in the variant benchmark fall within homopolymers and tandem repeats (due to their naturally higher mutation rate). However, such repeats make up only a small fraction of the total genome, so their stratified error rate is elevated in genome benchmarking compared with the genome-wide average (Figures 5B and S9A). These comparisons point to the need for standardized genome benchmarking metrics and stratifications to enable downstream users to interpret performance in a familiar context. One compromise could be to identify a set of clinically important or known variant sites in the diploid HG002 genome and report precision/recall measurements across these positions, which would be akin to variant benchmarking but without the confounding reference bias.

## DISCUSSION

Building on the widely used GIAB HG002 reference material, T2T-HG002v1.1 establishes the most comprehensive genomics benchmark ever constructed. Compared with CHM13, HG002 is more suitable as a benchmark due to its availability as a NIST reference material ^42^ and previously established variant benchmarks. ^1,18^ In addition, comprehensive functional genomics is enabled by openly consented ^39^ and commercially available LCL and iPSC cell lines from the NIGMS Human Genetic Cell Repository at Coriell. ^92^ Recent studies have collected additional primary samples, including blood and sperm, ^75^ making HG002 an ideal testbed for developing personalized genomics, where an individual’s complete, diploid genome serves as the basis for analyses. To date, human reference sequences have generally consisted of a single haplotype, i.e., one allele for each genomic position. ^8,11^ By contrast, the T2T-HG002 genome benchmark, like the cells it was derived from, consists of a diplotype, i.e., a pair of haplotypes, one inherited from each parent. The GQC software package is uniquely designed to evaluate diploid genomes in a haplotype-aware manner, which will drive the development of new methods for the reconstruction and analysis of diploid genomes. Toward this goal, the T2T-HG002 genome benchmark will be essential for measuring progress and is already being used to guide the development of new genome technologies such as sequencing, ^93^ genome assembly, ^94^ polishing, ^48^ and variant calling methods. ^13,95,96^ We also anticipate the development of haplotype-specific functional genomics benchmarks for RNA-seq, methylation, chromatin accessibility, and more to be anchored on the diploid reference genome presented here.

No genome assembly is infallible, and this includes T2T-HG002v1.1. The “Q100” project name invokes our aspirational goal of creating a virtually error-free 6 Gbp diploid human genome benchmark. However, this level of accuracy is practically impossible to achieve and validate with current technologies. First, the majority of sequencing data generated here was derived from cultured HG002 LCL cells, and some degree of somatic variation is expected relative to the NIST reference material DNA that was extracted from the same cell line but from a different batch of cells. ^42^ Even within the reference material itself, 85 high-confidence mosaic SNVs have been previously identified with variant allele fractions between 5% and 30%. ^97^ In this sense, the T2T-HG002 genome benchmark represents only a snapshot in time of the HG002 LCL line. The locations of mosaic variation in this cell line could be tracked and excluded from benchmarking evaluations, similar to how the low-confidence regions are currently handled. Second, benchmarking always suffers from a bootstrapping problem, as it is difficult to validate newly resolved regions of the genome that are not accessible to all sequencing technologies. Here, we show that *k-*mer-based methods for QV estimation miss certain classes of errors (e.g., loss of heterozygosity, long homopolymer errors, and repeat expansions/collapses) and can report FP errors if the *k*-mer database is incomplete (e.g., due to coverage bias in the raw sequencing data). Although it is possible to artificially increase *k*-mer quality by removing the known erroneous *k*-mers, this risks removing true heterozygous or paralog-specific variants. Thus, to construct a reliable benchmark, we chose a more conservative polishing approach that based decisions on an understanding of the errors being corrected and only used the *k*-mer-based methods to measure progress. In fact, most of the corrections we made had little impact on *k*-mer-based QV estimates due to their limitations in dealing with repetitive regions and heterozygous variation. The remaining regions of lower confidence include long homopolymers (i.e., >20 bp in length) and repetitive regions of the genome that could not be reliably mapped and validated with short reads (e.g., rDNAs, satellites, and segmental duplications). Given the size and potentially high mitotic recombination rate of rDNAs, ^98^ further improvements in sequencing technology are likely needed for their confident reconstruction. Excluding rDNA arrays, 99.9% of the diploid genome is covered by high-confidence genome benchmark regions in v1.1, compared with 85.2% with the v4.2.1 variant benchmark (Table S17).

Since the start of this project, improvements in sequencing and assembly methods have made it possible to create accurate genome benchmarks today with much less effort than described here. Through the course of this project, we came to a better understanding of the various error modes (and their root causes) and were able to suggest improvements to developers of the core sequencing and assembly technologies. This continuous improvement cycle has greatly reduced the number of corrections now needed after assembly and should eventually remove the need for assembly polishing altogether. Additionally, our GQC software is applicable to any diploid genome, including non-human species, bringing the concept of genome benchmarking to the broader fields of agricultural and biodiversity genomics.

We have demonstrated that prior variant-based benchmarks are limited in scope, prone to reference bias, and leave large parts of the genome unrepresented. Additionally, variant benchmarking stratifies errors into categories (SNVs, indels, and complex variants) based on their representation *relative to the reference*, which can obscure the true nature of the errors, particularly in repetitive regions where a single error can manifest as multiple variant calls of different types. Genome benchmarking fundamentally simplifies this problem and isolates the errors from any natural differences between the sample and reference. Ideally, all GIAB variant benchmarks can be updated and improved with a corresponding genome benchmark. However, there is a near-term need for improved variant benchmarks since variant calling remains the primary output of most genomics workflows, especially in clinical settings where the endpoint is a set of variants annotated for their clinical significance and the performance metrics are similar to those used for clinical testing. As methods to interpret the clinical significance of personal genome assemblies are developed, new approaches will be needed to understand how genome benchmarking performance metrics impact clinical interpretation. This human genome benchmark will be used to improve existing variant benchmarks, particularly in highly repetitive and copy-number variable regions of the genome, and help guide the formulation and interpretation of genome benchmarking metrics, which are not yet standardized.

T2T genome sequencing and assembly methods have enabled discoveries within previously hard-to-analyze regions of the genome, such as segmentally duplicated genes,^25 ,61^ amplified gene families,^99^ satellite DNAs,^100 ,101^ and tandem repeats.^102 ,103^ Continued progress toward the routine sequencing and analysis of complete genomes is necessary to fully illuminate the dark genome across population scales.^78^ In the near term, such analyses would require a high-quality pangenome^41^ comprising a large number of samples from which the entire genome of an individual could be inferred using less expensive sequencing methods.^104–106^ However, the rigorous validation of such pangenome-based methods has been impossible without a ground truth that encompasses the entire genome. The complete, diploid T2T-HG002 genome benchmark provides this missing validation framework, removes the performance ceiling of previous variant benchmarks, and will drive the development of new methods for low-cost, complete genome inference.

We expect the routine inference of T2T genomes, enabled by new sequencing technologies and bioinformatic methods that leverage the pangenome, will mark a new era of personalized genomics, whereby all analyses are performed in the context of an individual’s complete, diploid genome. A personalized genome provides the perfect reference against which an individual’s somatic and functional genomics data can be unambiguously mapped, eliminating the problem of reference bias. An open collection of T2T genomes with matched, long-read functional data would allow for the training of sequence-based computational models of the entire genome that, combined with existing knowledge bases and literature, could be used to annotate rich metadata such as gene annotations, regulatory elements, population allele frequencies, and disease risk predictions directly onto the personalized genome. This would ultimately allow clinical genome assessment to move beyond the interpretation of individual variants and toward the interpretation of whole genomes.

### Limitations of the study

Both the HG002 NIST reference material DNA and live HG002 cell lines are known to exhibit low levels of somatic variation. The T2T-HG002 genome benchmark is meant to reflect the majority allele *for the reference material*, which was derived from a single, homogenized pool of HG002 DNA. However, by nature, this reference material is a finite resource. If a new batch of reference material DNA were to be generated in the future, the genome benchmark should be updated to match.

Comparison of a sample’s sequence to a genome benchmark and reports of coverage and accuracy are inherently dependent on the sequence alignment algorithm used for comparison. A detailed analysis of the effects of the chosen alignment algorithm on benchmarking results was not performed for this study, but a cursory comparison of GQC statistics using minimap2 with those using winnowmap2 did not reveal significant differences in accuracy measurements.

## RESOURCE AVAILABILITY

### Lead contact

Requests for further information and resources should be directed to and will be fulfilled by the lead contact, Adam M. Phillippy (aphillippy@jhu.edu).

### Materials availability

No new materials were generated from this study. The sample is publicly available as NIST Reference Material 8391, Coriell LCL GM24385, PGP participant huAA53E0.

### Data and code availability

The T2T-HG002v1.1 assembly is available from NCBI GenBank under the HPRC BioProject PRJNA730823 with accession numbers GenBank: GCA_018 852605.3 (paternal) and GenBank: GCA_018852615.3 (maternal). No new sequencing data were generated for this project, but all data used here are organized in Table S1 and available from the project GitHub along with links to the browser hub, issue tracker, and associated analysis software (https://github.com/marbl/hg002). Any additional information required to reanalyze the data reported in this paper is available from the lead contact upon request.

## STAR★METHODS

### EXPERIMENTAL MODEL AND STUDY PARTICIPANT DETAILS

#### Cell line and reference material used for this study

The cell lines and reference material used for this study were derived from a male participant and his parents from the Personal Genome Project. DNA from these individuals is distributed by NIST as the reference materials RM8391 and RM8392 and cell lines and DNA are distributed by Coriell as NA24385 and GM24385, respectively.

#### Sample G-banded karyotyping of the GM24385 cell line

The lymphoblastoid cell line GM24385 was obtained from the NIGMS Human Genetic Cell Repository at the Coriell Institute for Medical Research and expanded to a total culture size of 2×1010 cells to create the HG002 lot of cells housed at NIST42. G-banded karyotype analysis was performed on this expansion of cells, harvested at passage 2 (post-cell line establishment). During the g-banding analysis, 73 metaphase cells were counted, and 12 metaphase cells were analyzed and karyotyped. Chromosome analysis was performed at a resolution of 400 bands or greater. DNA used for microarray was isolated from a frozen cell pellet (2×107 cells) using the Autopure LS instrument (Qiagen). DNA was genotyped using the Affymetrix Human SNP Array 6.0 (Affymetrix, Inc.).

Cytogenomic analysis of the g-banding and microarray results showed a karyotype of 92,XXYY[13]/46,XY,?inv(3)(q26.3q29)[3]/46,XY[57].arr[hg19](1–22)x2,(XY)x1 in this expansion lot of GM24385/HG002 cells (Figure S1). While the microarray analysis resulted in a normal, human male karyotype, the extensive g-banding data show a mostly diploid (78%) (Figure S1A), with some tetraploid cells (18%) (Figure S1B), and a very small number (4%) of cells showing an inversion on chromosome 3 (Figure S1C).

### METHOD DETAILS

#### Sequencing and initial assembly of HG002 (v0.7)

The assembly, polishing, patching, and validation of the HG002 genome made use of sequencing data from short, long, and ultralong datasets from the Human Pangenome Reference Consortium (HPRC) ^41^ and Genome in a Bottle Consortium (GIAB) ^42^ (Table S1). In addition to 170x coverage in HiFi Sequel II reads and 166x of ONT reads longer than 100kb used for the first assembly, roughly 200x coverage of HiFi Revio reads called with DeepConsensus v1.2 became available and were utilized during the polishing stage of the project. 82x coverage of R10 ONT reads greater than 100kb in length were also used for polishing.

The initial assembly of HG002 used for this manuscript, labeled “v0.7”, consists of a manually-curated set of scaffolds derived from verkko version 1.0 (commit f69fa7c0f62d4f1eaf2288a22c7e9e58fd1cacd7) assemblies (v0.1 through v0.6) run during the summer of 2022. ^28^ Roughly 170x coverage in HiFi Sequel II reads and 166x of ONT reads >= 100 kb were assembled, using short read k-mer “hapmers” determined using Illumina sequence (Table S1) from HG002, HG003, and HG004 (HG002 and its parents) for phasing. The v0.7 release was the last unpolished release in a series of curated verkko assemblies.

The initial assembly (assembly v0.1) had 25 T2T contigs, 28 T2T scaffolds, and 18 gaps. The initial QV was 63.1 measured with hybrid 31-mers (see Evaluation section). It was aligned to the CHM13 T2T reference to assign chromosome names to the graph and assembled sequences. This assembly resolved Chromosomes 1 maternal, 2 maternal, 3 both, 4 both, 7 both, 8 both, 9 both, 10 both, 11 both, 12 paternal, 16 both, 18 both, 19 both, 20 paternal, and Y. The three T2T scaffolds were Chr 1 paternal, Chr 12 maternal, and Chr 17 paternal with 1 gap each.

The assembly was resolved T2T based on manual evaluation and gap-filling following the protocols in Nurk et al. ^8^ and using the sg_sandbox repository archived in this manuscript’s software repository. ^114^ Briefly, chromosome X was re-used from the original HG002 ChrY publication. ^24^ ChrY from the publication was not used because it has a known inversion which the v0.1 assembly corrected. Chromosome 12 maternal was also previously resolved and re-used. The gap in chromosome 1 paternal was due to an un-popped bubble and was resolved by inspection of the graph. Chromosome 13 paternal was resolved by scoring multiple traversals of a gap and selecting a winner as in Nurk et al. This assembly was named v0.1. Assembly v0.2 was identical to v0.1 except resolved chromosome orientations were updated to match the CHM13 T2T reference.

Chr 5 maternal, Chr 13 maternal q-arm, and Chr 20 maternal had overlaps between the contigs at the assembly breaks. To confirm these overlaps we generated an independent assembly using Flye. ^108^ All ONT reads > 100 kb were trio-binned using Merqury ^52^ and assembled independently. In all cases, the Flye assembly confirmed the overlap size. This assembly after joining overlapping sequences was called v0.3.

Chromosome 14 paternal q-arm was resolved by comparing traversals through the graph and selecting the highest scoring one. Chromosomes 2 paternal also had a gap which was patched using a consensus from three ONT reads. The remaining chromosomes could not be resolved using this initial assembly graph. To resolve them, an additional assembly was generated using experimental MBG options (copycount_resolve commit 8e03e068ca64d986a9f983aab35ce4b1d4c4176, available as option –copycount-filter-heuristic since MBG v1.0.13) which performs extra simplification by removing low-coverage nodes during multiplex resolution. Chromosome 6 maternal and paternal were resolved based on the best-scoring traversal in the new assembly graph. Chromosome 17 maternal and paternal were patched to close gaps in the original assembly using the best-scoring traversals from the new assembly. This assembly was called v0.4.

Chromosome 5 paternal was resolved by taking the best traversal from the new assembly and lifting it back to the original graph with GraphAligner ^109^ alignment. The assembly was screened for Epstein-Barr virus (EBV) (AJ507799) sequences and mitochondrial human DNA (NC_012920). Any matching nodes were removed from the assembly to avoid redundancy. Representative mitochondrion and EBV sequences were selected by coverage and trimmed (if needed) to remove any redundant sequence on the ends. This assembly was called v0.5.

Nodes matching rDNA were identified using mash screen with the commands:

mash sketch -I unitigs.hpc.fasta -o sketch.msh

mash screen sketch.msh KY962518.fasta |awk ‘{if ($1 > 0.9 && $4 < 0.05) print $NF)’ > target.screennodes.out

where KY962518.fasta is the KY962518 reference rDNA sequence. The matching nodes were collapsed to a single node and telomere added to the graph to annotate ends of chromosomes. The script to perform this analysis (remove_nodes_add_telomere.py) is archived in this manuscript’s software repository. ^114^ This simplified the graph for the acrocentric chromosomes, allowing the distal regions to be resolved. In all except two cases, the majority of the distal arm was resolved in a single node. Two paternal distal regions (later determined to be Chr 13 paternal and Chr 22 paternal, see below) were tangled together in the graph with approximately 1.6 Mbp of 1.8 Mbp (in homopolymer-compressed space) shared between the two chromosomes. The two best paths through this region based on ONT alignments were selected to represent the distal regions. This assembly was called v0.6.

Two independent methods were used to assign the acrocentric chromosome ends to their correct chromosome. Sequence for all ten chromosome ends was generated from the assembly graph by aligning the rDNA repeat unit and DJ sequence to identify paths. These were assigned to a parental haplotype using trio markers. The paths could be resolved unambiguously except in the case of two paternal haplotypes which were nearly identical with few bubbles. For these two (Chr 13 and Chr 22), two paths were generated, each using the shared paths and alternating in the bubble, but the phasing between bubbles is likely inaccurate. This resulted in 10 candidate distal regions, 5 for each haplotype. Then, pstools ^110^ was used to identify k-mer based mappings of Hi-C sequences to the paternal and maternal assembly and the respective distal regions, independently. For each possible pair of distal region and q-arm in a haplotype, the pair with the maximum read support as well as the second-best pair were reported. Independently, Strand-seq data (Table S1) was used to identify best-buddy matches between the ends and q-arms of the chromosomes, again within a haplotype (see “Strand-seq-based clustering of acrocentric chromosomes”, below). The two methods agreed with the exception of Chr13/21 paternal where Hi-C swapped the assignments versus Strand-seq. The second-best matches in the Hi-C pstools results agreed with Strand-seq. FISH was used to identify characteristic WaluSat repeat arrays on the chromosome ends to disambiguate this assignment.^46^ The Hi-C assignment would indicate there is a weak WaluSat signal present on both Chr21 and Chr22 paternal while Strand-seq indicates that it would be present on Chr13 and Chr22. The images better support the signal on Chr13 and Chr22 and thus the Strand-seq assignment for these chromosomes was selected. The presence of WaluSat on the other chromosomes was also confirmed to be consistent with the assignments using Hi-C and Strand-seq data (Table S1). The rDNA on Chr13 paternal was short enough to be manually resolved. For the other acrocentric chromosomes, gaps corresponding to the FISH-estimated size of their rDNA arrays ^46^ were inserted into the sequence, resulting in the v0.7 assembly that was released in November 2022 and used as the starting assembly for the polishing described in “Polishing and patching of HG002v0.7 to create HG002v1.1”.

#### Strand-seq clustering of acrocentric chromosomes

As reported before, Strand-seq has a unique ability to preserve the strand inheritance of the whole maternal and paternal homologs. ^50^ This information can be used to to assign assembly contigs to their respective homologous chromosomes. We used Strand-seq (Table S1) to assign short and long arms of all acrocentric chromosomes (13, 14, 15, 21, and 22) to their respective paternal and maternal homologs. To do this, we extracted the last 5% of Strand-seq reads at the end of the short acrocentric arm (tail) and first 5% of Strand-seq reads from the beginning (head) of the long acrocentric arm. Only uniquely mapped reads (mapping quality >= 10) were considered. We do this across multiple single-cell strand-seq libraries (*n*=65) so we observe multiple independent assortments of acrocentric chromosomes in daughter cells. We then compare the Strand-seq strand-state (‘ww’ Watson-Watson, ‘wc’ Watson-Crick, and ‘cc’ Crick-Crick) between tail and head of each pair short and long acrocentric arm across all five acrocentric chromosomes. We assign short and long acrocentric arms based on the best agreement between tail and head strand-states. This means, the strand-state of short and long acrocentric arms that likely belong to each other share the same strand-strand state more often across multiple Strand-seq cells than the other pairs.

#### Crowd-sourced curation

In the initial two rounds of polishing and patching, randomly selected examples of different sets of potential corrections (see “Categories of issues/corrections to v0.7” and “Categories of issues/corrections to v0.9”) were assigned to volunteer curators using GitHub’s issue interface. Using a GitHub repository called “HG002-issues” ^114,141^ , quality issues with the v0.7 or v0.9 assembly were recorded as issues, assigned to curators, discussed in the comments, and eventually closed. Curators were supplied with XML-formatted session files to load into IGV,^122^ allowing identical aligned read data and genome annotations to be viewed against the v0.7 or v0.9 assembly in each problem region. In general, if a majority of the issues in a particular category were confirmed by curators to be genuine and correctable, that category’s corrections were applied in creating the next T2T-HG002 release.

The scripts used to populate, change the status of, and eventually close issues are publicly available in the “github_issues” software repository. ^115^

#### Read alignments

For all rounds of polishing, we aligned newly available sequencing reads using the alignment methods described in Mc Cartney et al. (2022). ^59^ Briefly, short Illumina, Element Biosciences, and PacBio Onso reads were aligned with BWA-MEM ^111^ (version 0.7.17-r1188) using default parameters, after which BAM files were sorted with SAMtools, mate pair coordinates were filled in with SAMtools fixmates, and PCR duplicates were marked with SAMtools markdup. Longer ONT and PacBio reads were aligned with Winnowmap2 (version 2.03) using a repetitive k-mer database of 15-mers for down-weighted seeding. ^112^ The scripts used to run Winnowmap2 and BWA-MEM are part of the T2T-Polish software package ^59^ and are included in the manuscript’s software archive. ^114^

#### Categories of issues/corrections to v0.7 (round 1)

##### Small corrections

###### Phase-switched and falsely homozygous sites.

In an effort to identify locations in the T2T-HG002v0.7 assembly where sequence from one of HG002’s parental haplotypes was present on the opposite HG002 haplotype, we ran Merqury on short read (Illumina) data from HG002 and its parents HG003 and HG004, and we performed Strand-seq analysis to phase the assembly and identify heterozygous sites with alleles on the wrong-haplotype. These regions were discovered by first running Merqury ^52^ on the HG002v0.7 assembly using 21-mer Meryl databases of Illumina HiSeq2500 PCR-free 2×250 reads (∼350bp insert size) from HG002, HG003, and HG004 (64x for child, 50x for parents) (Table S1), with which Merqury creates a database of hapmers and predicts phase blocks (num_switch=100, short_range=20000). Phase-switched Merqury regions were identified as locations where a maternal block is found on a paternal HG002v0.7 chromosome or a paternal block is found on a maternal HG002v0.7 chromosome.

The Strand-seq-based phasing was performed with the v14 parameterization of the PGAS pipeline. ^87,142^ Briefly, the strand-specific signal of Strand-seq was leveraged to identify phase-informative regions in the assembly. These regions were then phased with StrandPhaseR version #8b93668 ^116^ into sparse whole-chromosome haplotypes. These sparse haplotype blocks then served as the backbone in the final step with WhatsHap v1.1. WhatsHapv1.0 ^117^ then added additional phase information from the long reads to create more dense and reliable haplotype blocks.

We obtained 58 “high confidence” regions by intersecting the Merqury- and Strand-seq-flagged regions detected in the analysis above, and assigned them to volunteers for curation. Curators examined PacBio HiFi, ONT ultra-long, and Illumina reads in IGV, ^122^ annotated with the locations of haplotype-informative assembly k-mers and heterozygous sites in the assembly. Notably, when long read data were examined by curators, roughly half (25) of the issues were found by curators to be false positives (i.e., the v0.7 assembly consensus was found to be correct), so this set of corrections was not applied, and we pursued an alternative correction discovery method.

Curators had observed that the 23 true positive regions in the abandoned correction set described above nearly always had ONT reads that were long enough to stretch from correctly-phased heterozygous sites of the assembly into regions where the assembly had alleles on the wrong haplotype. Therefore, we used DeepVariant v1.5 ^57^ to call variants using alignments of R10 ONT reads (Tables S1 and S2) to the entire diploid assembly (subsequently referred to as “all-to-all” alignments), filtering the results for homozygous PASS calls with >=95% alternate allele frequency, >=10 reads coverage, and no variant call present on the alternate HG002 haplotype, keeping only the variants that were within regions of homozygosity of at least 5000 bases in the assembly:

# call variants withDeepVariant version 1.5.0:

# output hg002v0.7_matpat_r10_simplex_DV_1.5.vcf.gz

# create bed files of all variants in homozygous regions >=5 or 10kb

# with genotypes quality at least 10 and 95% allele fraction:

awk –F“\t” ’$3-$2>=5000 {print}’ hg002v0.7.haplotypemapping.pri.wm.withsimscores.hom.sort.uniq.bed > hg002v0.7.h aplotypemapping.pri.wm.withsimscores.hom.ge5kb.sort.uniq.bed

bedtools intersect -a hg002v0.7.haplotypemapping.pri.wm.withsimscores.hom.ge5kb.sort.uniq.bed -b hg002v0.7_matpat_r10_simplex_DV_1.5.allvars.bed -wo > hg002v0.7_matpat_r10_simplex_DV_1.5.vars.hom.ge5kb.sort.bed

awk –F“\t” ’$12>=10 && $13>=0.95 {print}’ hg002v0.7_matpat_r10_simplex_DV_1.5.vars.hom.ge5kb.sort.bed > hg002v0.7_matpat_r10_simplex_DV_1.5.vars.hom.ge5kb.gq10.af95.bed

# bed files with coordinates of the variants, as well as four column bed files for lifting:

awk –F“\t” ’{OFS=“\t”; print $7, $8, $9, “ID”NR, $10, $11, $12, $13, $14}’ hg002v0.7_matpat_r10_simplex_DV_1.5.vars.hom.-ge5kb.gq10.bed > hg002v0.7_matpat_r10_simplex_DV_1.5.vars.hom.ge5kb.gq10.varlocs.bed

awk -F“\t“ ’{OFS=“\t”; print $1, $2, $3, $4}’ hg002v0.7_matpat_r10_simplex_DV_1.5.vars.hom.ge5kb.gq10.varlocs.bed >\ hg002v0.7_matpat_r10_simplex_DV_1.5.vars.hom.ge5kb.gq10.varlocs.forlifting.bed

# lifted locations on opposite haplotypes of hq variants in hom regions:

liftOver hg002v0.7_matpat_r10_simplex_DV_1.5.vars.hom.ge5kb.gq10.varlocs.forlifting.bed ../../chainfiles/v0.7_mat_vs_pat/hg002v0.7.bothdirs.chain hg002v0.7_matpat_r10_simplex_DV_1.5.vars.hom.ge5kb.gq10.varlocs.althap.bed\ hg002v0.7_m atpat_r10_simplex_DV_1.5.vars.hom.ge5kb.gq10.varlocs.althap.unmapped

sort -k1,1 -k2,2n -k3,3n \ hg002v0.7_matpat_r10_simplex_DV_1.5.vars.hom.ge5kb.gq10.varlocs.althap.bed > hg002v0.7_matpat_r10_simplex_DV_1.5.vars.hom.ge5kb.gq10.varlocs.althap.sort.bed

# divide alt hap (lifted) variant positions between those that have a variant

# on the alt hap and those that don’t – note that these bed files are in the

# coordinates of the correct alternate location, not the homogenized variant!

bedtools intersect -a \ hg002v0.7_matpat_r10_simplex_DV_1.5.vars.hom.ge5kb.gq10.varlocs.althap.sort.bed -b\ hg002v0.7_matpat_r10_simplex_DV_1.5.allvars.sort.bed -v > hg002v0.7_matpat_r10_simplex_DV_1.5.vars.hom.ge5kb.gq10.no_vars_on_alt_hap.bed bedtools intersect -a hg002v0.7_matpat_r10_simplex_DV_1.5.vars.hom.ge5kb.gq10.varlocs.althap.sort.bed -b hg002v0.7_matpat_r10_simplex_DV_1.5.allvars.sort.bed > hg002v0.7_matpat_r10_simplex_DV_1.5.vars.hom.ge5kb.gq10.vars_on_alt_hap.bed

# join vars with alt coordinates for variants without a variant on the

# alt haplotype:

awk ’{OFS=“\t”; print $4, $0}’\ hg002v0.7_matpat_r10_simplex_DV_1.5.vars.hom.ge5kb.gq10.varlocs.bed | sort -k1,1 > hg002v0.7_matpat_r10_simplex_DV_1.5.vars.hom.ge5kb.gq10.varlocs.tmp.bed

awk ’{OFS=“\t”; print $4, $0}’ hg002v0.7_matpat_r10_simplex_DV_1.5.vars.hom.ge5kb.gq10.no_vars_on_alt_hap.bed | sort -k1,1 > tmp.bed

join tmp.bed hg002v0.7_matpat_r10_simplex_DV_1.5.vars.hom.ge5kb.gq10.varlocs.tmp.bed |

awk ’{OFS=“\t”; print $2, $3, $4, $5, $6, $7, $8, $10, $11, $12, $13, $14}’ | sort\ -k1,1 -k2,2n -k3,3n > hg002v0.7_matpat_r10_simplex_DV_1.5.vars.hom.ge5kb.gq10.varlocs.no_vars_on_alt_hap.withaltcoords.bed

# use pull_filtered_vcf_lines.pl script to pull appropriate VCF records from

# initial DeepVariant output:

pull_filtered_vcf_lines.pl > hg002v0.7_matpat_r10_simplex_DV_1.5.vars.hom.ge5kb.ge10reads.af95.no_vars_on_alt_hap.vcf

This resulted in a list of 1,633 sites with lost heterozygosity in the v0.7 assembly which were correctible using the corresponding DeepVariant call. Reassuringly, this set of corrections included all of the incorrect alleles (true positive corrections) in the Merqury- and Strand-seq-flagged regions which had been considered correct by curators in the previous curation step. This points to long-read discovery of sites with misphased alleles using DeepVariant being a more effective method for correction discovery than Merqury and Strand-seq phasing.

##### Small errors discovered from short-read homozygous calls

To discover small indel errors where the assembly was heterozygous while the sample appeared to be homozygous (i.e., places where a new, erroneous allele had probably been introduced into the assembly on one haplotype only), we examined two sets of DeepVariant v1.5 ^57^ homozygous non-reference calls. All short reads were aligned with BWA MEM to both paternal and maternal haplotype assemblies independently, causing autosomal reads to “pile up” on either the maternal or the paternal sequence of v0.7. These haplotype-specific assemblies consisted of single haplotypes of the v0.7 assembly combined as well as decoy sequences from the opposite haplotype’s sex chromosome (i.e., chrX added to the v0.7 paternal haplotype, or chrY added to the maternal haplotype), as well as the mitochondrial sequence and the sequence of EBV used to immortalize the HG002 cell line added to both hapotype-specific references. The resulting alignments are subsequently referred to as “all-to-one” alignments.

The first set of short reads aligned consisted of 100x coverage of paired 2×150 small insert reads from Element Biosciences, and the second set of reads consisted of 55x coverage of single-ended 1×100 Onso reads from PacBio (Table S1). We first merged the Element and Onso DeepVariant call sets produced by DeepVariant, and then extracted only the homozygous non-reference variant calls (which are equivalent to suggested corrections to the reference haplotype) that appeared in both sets of calls:

# do the intersect with bcftools merge (so we can see which were in which) bcftools merge SBB_PB/hg002_ssr_DV.vcf.gz \ element_SI/hg002v0.7_mat_element_PCR_free_2×150_100X_DV_1.5.vcf.gz | awk -F“\t” \ ’$1∼/#/ || ($10∼/1\/1/ && $11∼/1\/1/)

{print}’ | bgzip -c > element_SI_and_SBB_PB.mat.merge.hnr.vcf.gz

bcftools merge SBB_PB/hg002v0.7_pat_SSR_DV_1.5.vcf.gz element_SI/hg002v0.7_pat_element_PCR_free_2×150_100X_DV_1.5.vcf.gz | awk -F“\t” \ ’$1∼/#/ || ($10∼/1\/1/ && $11∼/\/1/) {print}’ | bgzip -c > element_SI_and_SBB_PB.pat.merge.hnr.vcf.gz

# find calls that are in both element and SBB sets:

bedtools intersect -a element_SI/hg002v0.7_pat_element_PCR_free_2×150_100X_DV_1.5.hnr.vcf -b SBB_PB/hg002v0.7_pat_SSR_DV_1.5.hnr.withheader.vcf > element_SI_and_SBB_PB.pat.noheader.vcf bedtools intersect -a \ element_SI/hg002v0.7_mat_element_PCR_free_2×150_100X_DV_1.5.hnr.vcf -b SBB_PB/hg002_ssr_DV.hnr.vcf > element_S_and_SBB_PB.mat.noheader.vcf

This approach yielded 4,203 corrections on the maternal haplotype and 4,104 on the paternal.

###### Small errors discovered from Element heterozygous calls.

We next used parental assemblies to phase heterozygous Element calls based on the allele observed in the maternal (HG004) or paternal (HG003) haplotype. To generate a list of proposed corrections from heterozygous DeepVariant calls on Element read alignments to one haplotype at a time (“all-to-one” alignments), we utilized diploid hifiasm (v0.18.9-r527) maternal (HG004) and paternal (HG003) assemblies generated from PacBio HiFi and Oxford nanopore reads and phased with HiC. These assemblies of HG002’s parents were aligned to their corresponding haplotype of HG002v0.7, and variants were then called with dipcall. Heterozygous sites in the parent that were seen to be homozygous in HG002 were used to determine which of the parent’s two haplotypes (“hap1” or “hap2”) had been inherited by the child. This yielded bed-formatted files against HG002v0.7 showing which of each parent’s assembly haplotypes were inherited at any spot in the v0.7 assembly.

In regions where DeepVariant made a heterozygous call against the HG002 assembly, we then selected the allele which was consistent with the inherited haplotype from the respective parental assembly (e.g. HG003 for paternal haplotype), but not observed in the other parent’s assembly.

# Sample commands for correcting maternal haplotype:

# use HiFi+ONT+HiC HG004 hifiasm assembly to phase element hets on mat that

# are consistent with the inherited HG004 haplotype.

# the regions where hap1 and hap2 from hg4 were inherited are in separate files,

# but they could be combined. They are also separated by whether the element GT

# was 0/1 or 1/2, but the final GT is always 1/1 after selecting the variant

# from hg4 and normalizing. In exploratory work, these

# seemed generally reliable, but likely contain a small number of errors where

# the hg4 assembly was wrong, particularly in homopolymers

# Find regions where each hg4 haplotype was inherited by clustering variants on

# each haplotype. Since the initial simpler merging had a few errors, we used a more

# stepwise approach: merge variants within 10kb and keep regions >20kb.

# merge remaining regions within 100kb and keep >200kb. merge remaining

# regions within 1Mb and keep >10Mb. Finally merge remaining regions with

# 70Mb

# uses bedtools v2.30.0, bcftools v1.17

gunzip -c hg4vhg2mat.pair.vcf.gz | awk ’{FS=OFS=“\t”} {if($11 ∼ / ^1\/1/)

print $1, $2–1,$2+length($4)}’ | mergeBed\ -i stdin -d 10000 | awk ’$3-$2>20000’ | mergeBed -i stdin -d 100000 | awk ’$3-$2>200000’ | mergeBed -i stdin -d 1000000 |\ awk ’$3-$2>1000000’ | mergeBed -i stdin -d 7000000 > hg4vhg2mat.pair.hap1inherited.v2.bed

gunzip -c hg4vhg2mat.pair.vcf.gz | awk ’{FS=OFS=“\t”} {if($10 ∼ / ^1\/1/)

print $1, $2–1,$2+length($4)}’ | mergeBed -i stdin -d 10000 | awk ’$3-$2>20000’ | mergeBed -i stdin -d 100000 | awk ’$3-$2>200000’ | mergeBed -i stdin -d 1000000 |\ awk ’$3-$2>1000000’ | mergeBed -i stdin -d 7000000 > hg4vhg2mat.pair.hap2inherited.v2.bed

subtractBed -a hg4vhg2mat.pair.hap1inherited.v2.bed -b hg4vhg2mat.pair.hap2inherited.v2.bed > hg4vhg2mat.pair.hap1inherited.nohap2.v2.bed

subtractBed -a hg4vhg2mat.pair.hap2inherited.v2.bed -b hg4vhg2mat.pair.hap1inherited.v2.bed > hg4vhg2mat.pair.hap2inherited.nohap1.v2.bed

#Intersect with Element-DV het calls where all data is aligned to maternal

#element vcf from #https://s3-us-west-2.amazonaws.com/human-pangenomics/T2T/HG002/assemblies/polishing/HG002/v0.7/variants/DeepVariant/all_to_one_element_PCRfree/hg002v0.7_mat_element_PCR_free_2×150_100X_DV_1.5.vcf.gz

# also add calls from maternal vs maternal to ignore hets that are already on

# the other haplotype, so likely not errors

dvc get –rev c40d5cf https://github.com/ndwarshuis/hg2-t2t-benchmark-dev.git/pipelines/hg2-trio-dipcall/results/dipcall/hg2_mat_verkko

gunzip -c hifiasm/hg2_mat_verkko/hg2.fixed.vcf.gz |

awk ’{FS=OFS=“\t”} {if(!($1 ∼ / ^#/)) $1=$1“_matERNAL”; print}’ | bgzip -c > hifiasm/hg2_mat_verkko/hg2.fixedmat.vcf.gz tabix hifiasm/hg2_mat_verkko/hg2.fixedmat.vcf.gz

# merge vcfs (will have errors in a few places where complex vars

# have differing representations

bcftools merge hg002v0.7_mat_element_PCR_free_2×150_100X_DV_1.5.vcf.gz hg4vhg2mat.pair.vcf.gz hg2_mat_verkko/hg2.fixedmat.vcf.gz –force-samples > hg4vhg2matvelementDVmatmerge.vcf

# Find element-DV 0/1 calls that match hg4 hap1 where it was inherited,

# and the variant is not in the maternal haplotype

intersectBed -a hifiasm/hg4vhg2matvelementDVmatmerge.vcf -b hg4vhg2mat.pair.hap1inherited.nohap2.v2.bed -header | awk ’$1 ∼ / ^#/

|| ($10 ∼ / ^0\/1/ && $11 ∼ / ^1\/1/ &&!($13 ∼ / ^1/))’ |

bcftools view -s results/dipcall/hg4/hg2.hap1.bam –trim-alt-alleles |

bcftools norm -f assembly.v0.7.fasta > hg4vhg2mat_hg2elementhet01_hap1inherited.vcf

# Find element-DV 1/2 calls that match hg4 hap1 where it was inherited,

# and the variant in hg4 is not in the maternal haplotype

intersectBed -a hifiasm/hg4vhg2matvelementDVmatmerge.vcf -b hg4vhg2mat.pair.hap1inherited.nohap2.v2.bed -header | awk ’$1 ∼ / ^#/

|| ($10 ∼ / ^1\/2/ && (($11 ∼ / ^1\/1/ &&!($13 ∼ / ^1/)) ||

($11 ∼ / ^2\/2/ &&!($13 ∼ / ^2/))))’ | bcftools view -s results/dipcall/hg4/hg2.hap1.bam –trim-alt-alleles |

bcftools norm -f assembly.v0.7.fasta > hg4vhg2mat_hg2elementhet12_hap1inherited.vcf

# Find element-DV 0/1 calls that match hg4 hap2 where it was inherited,

# and the variant is not in the maternal haplotype

intersectBed -a hifiasm/hg4vhg2matvelementDVmatmerge.vcf -b hg4vhg2mat.pair.hap2inherited.nohap1.v2.bed -header | awk ’$1 ∼ / ^#/

|| ($10 ∼ / ^0\/1/ && $12 ∼ / ^1\/1/ &&!($13 ∼ / ^1/))’ |

bcftools view -s results/dipcall/hg4/hg2.hap2.bam –trim-alt-alleles |

bcftools norm -f assembly.v0.7.fasta > hg4vhg2mat_hg2elementhet01_hap2inherited.vcf

# Find element-DV 1/2 calls that match hg4 hap2 where it was inherited,

# and the variant in hg4 is not in the maternal haplotype

intersectBed -a hifiasm/hg4vhg2matvelementDVmatmerge.vcf -b hg4vhg2mat.pair.hap2inherited.nohap1.v2.bed -header | awk ’$1 ∼ / ^#/

|| ($10 ∼ / ^1\/2/ && (($12 ∼ / ^1\/1/ &&!($13 ∼ / ^1/)) ||

($12 ∼ / ^2\/2/ &&!($13 ∼ / ^2/))))’ |

bcftools view -s results/dipcall/hg4/hg2.hap2.bam –trim-alt-alleles |

bcftools norm -f assembly.v0.7.fasta > hg4vhg2mat_hg2elementhet12_hap2inherited.vcf

This approach yielded a total of 9,061 corrections on the maternal haplotype and 9,819 corrections on the paternal.

##### Corrections to larger regions

###### T2T-Polish-discovered issues.

T2T-Polish ^59^ was used to discover regions of HG002v0.7 with unusually high or low coverage or high amounts of read clipping. First, both PacBio HiFi Sequel reads called with DeepConsensus v1.1 and “R9” Oxford nanopore reads called with guppy remora v6.1.2 (Table S1) were aligned separately to the entire diploid v0.7 assembly with winnowmap2^112^ (“all-to-all” alignments), after which “issue” bed files were created with T2T-Polish:

# create file of repetitive k-mers for winnowmap:

meryl count k=15 hg002v0.7.fasta.gz output merylDB

# add “compress” for homopolymer compression meryl print greater-than distinct=0.9998 merylDB > repetitive_k15.txt

# align HiFi and ONT reads with winnowmap2:

winnowmap –MD -W repetitive_k15.txt -ax map-pb -I12g -t$cpus

hg002v0.7.fasta.gz hifi_dcv1.1.fastq.gz

winnowmap –MD -W repetitive_k15.txt -ax map-ont -I12g -t$cpus

hg002v0.7.fasta.gz ont_guppy_6_1_2_remora.fastq.gz

# run T2T-Polish scripts to find coverage, quality, and clipping issues:

# generate microsatellite bed files in directory “pattern” cd pattern

$T2TPolish/pattern/microsatellites.sh hg002v0.7.fasta.gz

# create assembly bed files for “low_support.sh”, called by issues.sh:

awk -F “ \t” ’{OFS=“\t”; print $1, 0, $2}’ v0.7.fasta.fai > v0.7.bed

cat merqury/v0.7_hybrid/*_only.bed | bedtools merge -i - > v0.7.err.bed

touch v0.7.exclude.bed # no excluded regions

awk -F “ \t” ’{OFS=“\t”; print $1, 0, 10000; print $1, $2–10000, $2}’

v0.7.fasta.fai > v0.7.telo.bed

# run issues.sh:

$T2TPolish/coverage/issues.sh hg002v0.7_hifi_dcv1.1.pri.paf hg002v0.7_hifi_dcv1.1.pri v0.7 HiFi

$T2TPolish/coverage/issues.sh

hg002v0.7_guppy_6_1_2_remora.pri.paf hg002v0.7_guppy_6_1_2_remora v0.7 ont

The union of these two issue bed files (one for HiFi, one for ONT) contained 344 regions covering roughly 2 megabases of consensus: 35 issues were based on ONT evidence, 337 on HiFi evidence, and 28 were flagged using both platforms.

##### Issues discovered with Flagger/SecPhase

Flagger ^10,118^ is a read-mapping-based pipeline developed for evaluating phased genome assemblies. It works by taking long reads mapped to the assembly, computing read depth of coverage along the assembly and using Gaussian Mixture Model (GMM) to infer which parts of the assembly are potentially problematic. It can infer three types of misassemblies; erroneous blocks (regions with misjoins or high base-level error rate), false duplications and collapsed blocks. It also incorporates mapping quality of read alignments as an additional signal to detect false duplications.

Flagger v0.2 was run on the same read alignments used for the T2T-Polish analyses (see “T2T-Polish-discovered issues”), after which regions which were flagged for both the HiFi alignments and the ONT alignments were gathered using bedtools intersect, ^124^ resulting in 159 assembly regions (called “Flagger Intersect Regions” in the issues GitHub repository), 34 of which overlapped with at least one of the 344 (union of HiFi and ONT) T2T-Polish-called regions (these Flagger calls are available on AWS at https://s3-us-west-2.amazonaws.com/human-pangenomics/index.html?prefix=T2T/HG002/assemblies/qc/flagger/).

###### Patches to problematic regions using a newer assembly (HG002v0.8).

All patch corrections to HG002v0.7’s larger problematic regions used sequence from a later assembly that had been run with newer read datasets (Table S1) in May 2023 using verkko version 1.3.1 (commit 0268963fb2eda147e9dfb8d4b0291fa06d47b4bb). This assembly was labeled HG002v0.8, and its scaffolds, labeled with human chromosome assignments, are available on AWS (source data and assembly URL in Table S1).

In preparation for applying HG002v0.8 patches to HG002v0.7, a set of 137 “problem regions” were compiled (see below). Of these, regions which could be successfully lifted from HG002v0.7 to HG002v0.8 in one piece after adding 2000 bases of buffer sequence to both the 5^′^ and 3^′^ end were curated by viewing their read alignments in IGV to assess whether the HG002v0.8 consensus represented a significant improvement over the HG002v0.7 consensus.

These 137 regions were composed of the following sets: First, 18 non-acrocentric problem regions that were both called by “Flagger” as collapses, duplications, or errors and marked as issues by the T2T-Polish pipeline. So-called “Intersect” regions were then determined by taking the intersections of regions tagged by the program Flagger as “Col”, “Dup”, or “Err” and regions also marked as an issue by the T2T-Polish pipeline when run on both HiFi and ONT primary alignments of reads to the entire diploid v0.7 assembly. Second, an additional twelve regions that contained a high density of DeepVariant homozygous non-reference calls on (a) three different long read alignment sets (HiFi/DeepConsensusv1.1, Duplex ONT, and R10 simplex ONT) (“all-to-all” alignments) or (b) alignments of high-accuracy short reads, both Element and Onso, to single haplotypes of the v0.7 assembly (“all-to-one” alignments) were included to potential patching. The final set of potential patch regions were those that had an unusual amount of sequence difference between v0.7 and v0.8. These were 10kb windows of v0.7 which had uniquely liftable regions to v0.8, but which also had at least 200 bases of difference between the two assemblies within the liftover chain. There were 122 of these intervals, 15 of which overlapped with one of the 30 regions in the first set described above. After curation, 74 of the large regions found with these methods were correctable using patches from the HG002v0.8 assembly.

##### Final assembly corrections applied to v0.7

The corrections described above fell into the following categories:

- “False Hets”: this category included short read homozygous calls and long read homozygous calls (7,849 corrections)
- “False Homs”: this category included parent assembly-phased short read heterozygous calls and phase-switched and falsely homozygous long read heterozygous calls (11,726 corrections)
- “Het sites with errors”: this category included short read heterozygous calls where one allele disagreed with HG002v0.7 (7,965 corrections)
- “Large structural errors”: this category includes the large regions identified by T2T-Polish and flagger and were improved in HG002 v0.8 (74 corrections, each replacing an average of 5,081 bp and adding or subtracting, on average, 2,080 bp)

Of the 27,540 small corrections, 97 fell within the boundaries of the 74 larger patch corrections described above and were therefore not included. This resulted in a set of 27,517 small and large corrections which were applied to the HG002v0.7 assembly using bcftools:

cat./ ../assemblies/v0.7.fasta | bcftools consensus -c \

hg002v0.7_to_hg002v0.9.chain corrections_large_and_small.withheader.vcf.gz \

> v0.9.fasta

The distribution of sizes for this correction set shows that the vast majority of corrections were one-base deletions from HG002v0.7 (Figure S2). The resulting corrected assembly, named T2T-HG002v0.9, was released in July 2023.

#### Categories of issues/corrections to v0.9 (round 2)

##### Corrections to problem areas in “difficult” regions

###### Correction of errors identified in centromeres.

Errors in the centromeric regions within T2T-HG002v0.9 assembly were identified by running NucFreq (v0.1) ^56^ with the following command:

NucPlot.py {input.bam} {output.png} –regions {centromeric_region_coordinates} -r {repeatmasker.out}

NucFreq plots were visually inspected to identify potential collapses in sequence, misjoins, or other types of assembly errors by looking for deviations in the first and second most common base relative to the mean. Seven centromeres were identified as having potential misassemblies: chr4_MATERNAL, chr4_PATERNAL, chr5_PATERNAL, chr6_MATERNAL, chr6_PATERNAL, chr13_PATERNAL, and chr17_MATERNAL. To patch the assembly errors in these centromeres, we generated a hifiasm v0.19.5 assembly ^54^ using the following command:

hifiasm -o {output_dir_prefix} -t{num_of_threads} –ul $(cat {ont_fastq.fofn} |

sed -z ’s/\n/,/g;s/,$/\n/’) −1 {mat.yak} −2 {pat.yak}

$(cat {pacbio_hifi_fastq.fofn})

We aligned HG002v0.9 centromeric contigs to the HG002 hifiasm assembly using minimap2 v2.28 ^113^ using the following parameters: -I 15G -a –eqx -x asm20 -s 5000 and extracted the corresponding centromeric regions that spanned the assembly errors in both the HG002v0.9 assembly and hifiasm assembly using seqtk (v1.4). Then, we ran RepeatMasker (v4.1.0) on each of the fasta files using the following parameters: {-species human -dir {output_dir} -pa {num_of_threads}. We also ran StringDecomposer (v1.0.0) on each of the fasta files using α-satellite monomer inputs specific to each chromosome and derived from the T2T-CHM13 v2.0 genome. Using the StringDecomposer outputs, we manually patched the HG002v0.9 genome assembly with the hifiasm assembly. We validated the successful repair of these regions by aligning HG002 PacBio HiFi data to the HG002v0.9 assembly containing the repaired centromeric contigs with the following command:

pbmm2 align –log-level DEBUG –preset SUBREAD –min-length 5000 -j 8.

We then ran NucFreq on each centromeric region using the same command as above and assessed whether the centromeric regions had first and second most common base frequencies similar to the mean of the region. This confirmed that the following centromeres were successfully repaired: chr4_MATERNAL, chr4_PATERNAL, chr5_PATERNAL, chr13_PATERNAL, and chr17_MATERNAL. Two centromeres still had assembly errors (chr6_MATERNAL, chr6_PATERNAL) that were unable to be repaired.

The 19 regions corrected on seven HG002v0.9 centromeres had the following v0.9 coordinates:

chr4_MATERNAL:49232779–49280263, chr4_MATERNAL:51637922–51647612, chr4_PATERNAL:52166546–52240961, chr4 _PATERNAL:52712713–52997607, chr5_PATERNAL:46790217–46791543, chr5_PATERNAL:47576796–47581721, chr5_PATERNAL:49782320–49787412, chr5_PATERNAL:51147396–51149093, chr5_PATERNAL:52219891–52225667, chr6_MATERNAL:62162402–62167836, chr6_PATERNAL:59153776–59162947, chr6_PATERNAL:59355877–59361991, chr6_PATERNAL:61597046–61614878, chr6_PATERNAL:62187141–62191384, chr6_PATERNAL:62281724–62324340, chr6_PATERNAL:62686192–62728299, chr6_PATERNAL:62762429–62774151, chr13_PATERNAL:11176318–11202477, chr17_MATERNAL:25766051–25799993

###### Correction of errors identified in telomeres.

Reads from Oxford Nanopore Technologies (ONT) telomere enrichment sequencing (Telo-seq) were generated using R10 chemistry with v4.3 SUP basecalling. Since the telomere analysis was begun before the release of v0.9, this analysis was completed using the v0.7 assembly and then carried over to determine corrections to v0.9. Reads were initially mapped to the HG002v0.7 phased reference genome using minimap2 (v2.26), ^113^ and single nucleotide polymorphisms (SNPs) and insertion-deletion variants (indels) were called using FreeBayes (v1.3.7). ^123^ Variants were filtered to retain only homozygous calls using bcftools (v1.17), and a consensus sequence was generated incorporating these variants into the reference sequence. The ONT telo-seq reads were subsequently re-mapped to this consensus sequence, and final corrections were made manually by curation using the Integrative Genomics Viewer (IGV v2.16.2) ^122^ and Geneious Prime (v2023.1).

Once this analysis was completed, the corrected telomere consensus sequences were compared to the newly-released HG002v0.9 by first extracting 100,000 base pairs from each end of each chromosome into a FASTA formatted file, then aligning the curated telomere sequences to them with minimap2 ^113^ using the parameters:

minimap2 -x asm5 -g 50000 -n 0 -f 0 teloseqs.fasta v0.9.telomere.fasta >> p_teloarms_ONTv14_to_v0.9.minimap2.out

After filtering the resulting alignments to require the tag “dv:f:0.00”, a shell script was used to generate a VCF patching the correct coordinates into the v0.9 assembly see the software repository for this manuscript for the exact commands performed to do the patching. ^114^

###### Extension of v0.9 rDNA flanking sequences into the rDNA gap.

With the exception of the rDNA array on the paternal copy of Chr13 (whose length was corrected using ultra-long ONT reads, see the section “Lengthening of paternal Chr13’s rDNA array using spanning ONT reads”), the rDNA arrays of v0.7 contained Ns approximating the length of each array flanked by low-accuracy consensus. Generation of new, more accurate flanking sequence for the rDNA arrays was done with the script “improve_gaps_ont.py” ^114^ , which iteratively extends unique region into the tangles when there is a clear majority (best > 1.5* second_best) of ONT reads which are anchored on the unique region and support the extension.

These new contigs were then aligned to the T2T-HG002v0.9 assembly with minimap2 and the alignments were used to choose the best matches between extended contigs and flanking sequences.

From the minimap2 results, high identity mappings were extracted:

# find contig to chromosome mapping from minimap2 output:

grep ’dv:f:0.000’ patchv2_to_0.9.out |

awk -F“\t” ’{OFS=“\t”; print $6, $1}’ | sort | uniq |

sed ’s/_0/_P/’ | sed ’s/_1/_Q/’ | awk ’$1!=“chr13_PATERNAL” {print}’

> patch_v2_contig_map.txt

A fasta-formatted file of flanking sequences surrounding the rDNA arrays was created: export BED=$1

export ASSEMBLYFASTA=/data/Phillippy/projects/HG002_diploid/assemblies/v0.9.fasta export NACROS=‘cat $BED | wc -l‘

export FLANKFILE=‘echo $BED.flank.fasta | sed ’s/.bed//’‘

echo $NACROS“ lines in ”$BED

rm -f $FLANKFILE

for i in ‘seq 1 $NACROS‘; do

export CHROM=‘head -$i $BED | tail −1 | awk -F “ \t” ’{print $1}’‘

export START=‘head -$i $BED | tail −1 | awk -F “ \t” ’{print $2}’‘

export END=‘head -$i $BED | tail −1 | awk -F “ \t” ’{print $3+1}’‘

export ENDP=‘echo $END + 2000000 | bc‘

echo “Found ” $CHROM 1 $START $END $ENDP

(echo “>“$CHROM”_P”; SAMtools faidx $ASSEMBLYFASTA $CHROM:1-$START | tail -n +2) >> $FLANKFILE

(echo “>“$CHROM”_Q”; SAMtools faidx $ASSEMBLYFASTA $CHROM:$END-$ENDP | tail -n +2) >> $FLANKFILE

done

samtools faidx $FLANKFILE

Then each high-identity rDNA-matching contig sequence was aligned to its corresponding v0.9 flanking sequence one contig at a time:

export FULLASSEMBLY=v0.9.fasta

export ASSEMBLY=rDNA_N_coords.v0.9.flank.fasta export PATCHSEQS=patch_v2/rdna_patch_v2.fasta

# map has v0.9 chrom with _[PQ], then contig from Dima’s file

export PATCHMAP=patch_v2_contig_map.txt export POUTPUT=pside_patch_to_v0.9.minimap2.out export QOUTPUT= qside_patch_to_v0.9.minimap2.out rm -f $POUTPUT $QOUTPUT

for contig in ‘awk ’{print $1}’ $PATCHSEQS.fai‘; do

export CONTIG=$contig

export CHROMEND=‘awk ’$2==ENVIRON[“CONTIG”] {print $1}’ $PATCHMAP‘

export CHROM=‘echo $CHROMEND | sed ’s/ATERNAL_P/ATERNAL/’ | sed ’s/ATERNAL_Q/ATERNAL/’‘

export END=‘echo $CHROMEND | sed ’s/.*_//’‘

export CHROMLEN=‘awk -F“\t” ’$1==ENVIRON[“CHROM”] {print $2}’ FULLASSEMBLY.fai‘

export LEN=‘awk -F“\t” ’$1==ENVIRON[“CHROMEND”] {print $2}’ $ASSEMBLY.fai‘

#export START=‘echo $LEN-100000 | bc‘;

echo “CONTIG: “$CONTIG ” CHROMEND: “$CHROMEND “END: “$END ” CHROM: “$CHROM ” LENGTH: “$CHROMLEN

“CHROMENDLEN: “$LEN

samtools faidx $ASSEMBLY $CHROMEND > assembly.fasta samtools faidx $PATCHSEQS $CONTIG > patchseq.fasta if [[

$END == “P” ]]; then

export OUTPUT=$POUTPUT

elif [[ $END == “Q” ]]; then

export OUTPUT=$QOUTPUT

fi

minimap2 -x asm5 -g 50000 -n 0 -f 0 patchseq.fasta assembly.fasta |

grep ’dv:f:0.000’ >> $OUTPUT

done

The resulting minimap2 PAF records were used to extract patch coordinates to input to the script “create_patch_vcf.py” ^114^ , which creates a correction VCF file which was included in the final creation of the v1.0 assembly:

export V9FASTA=v0.9.fasta

export RDNAFASTA=rdna_patch_v2.fasta

export PPATCHCOORDS=pside_patch_to_v0.9.minimap2.out

export QPATCHCOORDS=qside_patch_to_v0.9.minimap2.out

export V9NCOORDS=rDNA_N_coords.v0.9.bed

export COORDFILE=v0.9.rdna_patch_v2.coords.txt

rm -f $COORDFILE

export VCFFILE=v0.9.rdna_patch_v2.vcf

rm -f $VCFFILE

for contig in ‘awk -F “ \t” ’{print $6}’ $PPATCHCOORDS‘; do

export contig=$contig

export CHROMSIDE=‘awk -F “ \t” ’$6==ENVIRON[“CONTIG”] {print $1}’ $PPATCHCOORDS‘;

export CHROM=‘echo $CHROMSIDE | sed ’s/L_P/L/’ | sed ’s/L_Q/L/’‘

export V9START=‘awk -F “ \t” ’$6==ENVIRON[“CONTIG”] {print $3+1}’ $PPATCHCOORDS‘;

export PATCHCOORDS=‘awk ’$6==ENVIRON[“contig”] {print $8+1“ ”$7}’ $PPATCHCOORDS‘;

export PATCHSTART=‘echo $PATCHCOORDS | sed ’s/.*//’‘

export PATCHEND=‘echo $PATCHCOORDS | sed ’s/.* //’‘

export V9END=‘echo “$V9START + $PATCHEND – $PATCHSTART” | bc‘

echo -e “$CHROM\t$V9START\t$V9END\t$CONTIG\t$PATCHSTART\t$PATCHEND\t+” >> $COORDFILE done

for contig in ‘awk -F “ \t” ’{print $6}’ $QPATCHCOORDS‘; do

export contig=$contig

export CHROMSIDE=‘awk -F “ \t” ’$6==ENVIRON[“CONTIG”] {print $1}’ $QPATCHCOORDS‘;

export CHROM=‘echo $CHROMSIDE | sed ’s/L_P/L/’ | sed ’s/L_Q/L/’‘

export V9QSTART=‘awk -F “ \t” ’$1==ENVIRON[“CHROM”] {print $3+1}’ $V9NCOORDS‘

export V9FLANKEND=‘awk -F ^′′^ \t” ’$6==ENVIRON[“CONTIG”] {print $4}’ $QPATCHCOORDS‘;

export PATCHCOORDS=‘awk ’$6==ENVIRON[“contig”] {print $8+1“ ”$7}’ $QPATCHCOORDS‘;

export PATCHSTART=‘echo $PATCHCOORDS | sed ’s/.*//’‘

export PATCHEND=‘echo $PATCHCOORDS | sed ’s/.* //’‘

export V9PATCHEND=‘echo “$V9FLANKEND + $V9QSTART – 1” | bc‘

export V9PATCHSTART=‘echo “$V9PATCHEND – $PATCHEND + $PATCHSTART” | bc‘

echo -e “$CHROM\t$V9PATCHSTART\t$V9PATCHEND\t$CONTIG\t$PATCHSTART\t$PATCHEND\t-” >> $COORDFILE

done

# extract patch VCF:

python3 create_patch_vcf.py -c $COORDFILE -r $V9FASTA -p $RDNAFASTA –vcf $VCFFILE

###### Lengthening of paternal Chr13’s rDNA array using spanning ONT reads.

We discovered that two reads from an ultra-long Dorado-called ONT dataset (read names 03fb2d40–804f-418c-9195–872db2ea628e and 0bba627b-004a-42b0–8ff9-a76f9ca9ec6f) spanned the chr13_PATERNAL rDNA array when aligned to the v0.9 assembly. The paternal Chr13 rDNA array had been assembled with two copies of the rDNA unit in the v0.7 assembly, and had remained that way in the v0.9 assembly. The new spanning reads were aligned with clipping by minimap2 ^113^ because they contained more copies of the rDNA repeat unit than the v0.9 reference. Comparison of the two spanning reads to each other as well as to the assembly with ModDotPlot ^119^ showed that the ONT reads actually had six copies of the repeat unit between the non-rDNA ends, i.e., four more units than the v0.9 assembly has (Figure S4).

We found that a verkko assembly done with ONT duplex reads ^143^ had assembled the entire chr13_PATERNAL rDNA array with the same number of copies as the spanning ultralong ONT reads, so we extracted that section of the duplex assembly with 500 kb flanks and used it as a patch to the v0.9 assembly, determining the coordinates by matching the flanking regions of v0.9 with the flanks of the duplex assembly using minimap2.

##### Correction of small errors

We again utilized DeepVariant ^57^ calls (Table S2) to apply trio-based approaches to phase small corrections before applying them to v0.9. Three variant sets were filtered and included in the corrections made to v0.9 to obtain v1.0.1:

1. Taking advantage of the availability of new high-accuracy, higher depth-of-coverage short reads from the entire family trio, DeepTrio was run on Onso and Element reads from HG002, HG003, and HG004 aligned separately to either the maternal+Y of the paternal+X v0.9 references (Table S1, Table S2). High-confidence HG002 variants which appeared in both the Onso and the Element call sets were filtered for Mendelian consistency and to remove calls in the VDJ regions or in regions with high depth of coverage, as well as indel calls of 10 or more bases, which were observed to be potential somatic variants. This yielded 1,306 corrections.
2. To rescue short read-based calls that were filtered out of the first group, we aligned Onso, standard and long-insert Element, and Illumina reads from HG002, HG003, and HG004 to the separate maternal+Y and paternal+X v0.9 fasta files (“all-to-one” alignments) (Table S1). Then we called variants using DeepTrio (Table S2), merged and genotyped the gvcfs using GLnexus, and phased the HG002 variants from each technology using rtg-tools-3.12.1/rtg mendelian with the –Xphase –all-records options. Variant alleles phased to maternal or paternal haplotype with GQ and DP greater than 20 in all 3 individuals and DP less than double the median coverage in HG002 were identified as potential errors. We removed any variants in IGH, IGL, or IGK regions because they rearrange somatically. We also called variants with DeepVariant from HiFi reads aligned to the combined maternal and paternal haplotypes (“all-to-all” alignments). Based on manual curation, we kept phased short read variants if they had a homozygous or heterozygous HiFi call with the same allele, or if they were 1 bp indels in homopolymers where HiFi is less reliable. Example commands used to do this are available in the software repository for this manuscript. ^114^ This yielded 2,998 corrections.
3. Finally, 1,505 homozygous variant calls in the HiFi “all to all” DeepVariant set above (Table S2) were filtered to require GQ>=20 and DP<75 and included as additional corrections.

##### Final assembly corrections applied to v0.9

When the three categories of small corrections were combined and filtered to remove variants that lie within the larger patched regions, 4,482 small variants remained (displayed in blue in the correction size histogram in Figure S2).

Once v1.0 was created, it was discovered that we had inadvertently introduced ambiguity codes for three base pairs into the assembly due to heterozygous sites in the VCF file that was used as input to “bcftools consensus”. These three sites were at the following (v1.0) positions:

chr1_PATERNAL:73256576 K

chr3_PATERNAL:42065937 Y

chr8_MATERNAL:126720079 M

The decision was made to correct each of these three sites to what looked to be the most likely ATGC nucleotide. This resulted in the chr1_PATERNAL K and the chr3_PATERNAL bases being replaced with T’s, and the chr8_MATERNAL base being replaced with an A. The resulting assembly after these three corrections were applied was designated T2T-HG002v1.0.1 and was released and submitted to GenBank in October 2023.

#### Categories of issues/corrections to v1.0 (round 3)

The polishing and patching of T2T-HG002v1.0.1 to create v1.1 (“round 3”) attempted to address issues that were raised by several QC tools. Specifically, we applied filtered variant call set corrections created by DeepVariant ^57^ and DeepPolisher ^48^ and also created and applied assembly patches, where possible, to the following problem regions: 1) issues submitted to the GitHub “HG002-issues” repository, ^141^ 2) so-called “NIST error exclusion regions” (see the section “DeepVariant calls and NIST exclusion regions”, below), 3) regions with large numbers of DeepPolisher corrections which hadn’t survived filtering, 4) regions surrounding unresolved Sniffles ^58^ calls, and 5) suspect regions discovered by a new version of Flagger, v0.4.0 (see “Results of re-running Flagger on the T2T-HG002v1.0.1 assembly”).

##### Patching v1.0.1 with alternative assembly consensus

Because there were suspected erroneous regions of v1.0.1 for which we were unsure of the correct HG002 consensus sequence, we attempted to use two newer alternative assemblies’ consensus to repair suspect v1.0.1 regions. Specifically, in cases where the two new assemblies agreed with each other but disagreed with the v1.0.1 assembly, we constructed a patch to correct v1.0.1 with the newer sequence. To do this, we used two python scripts called “retrieve_patchseqs_from_bam.py” and “create_patch_vcf.py” ^114^ , which parse minimap2 alignments of two alternative assemblies to the unpolished reference to develop a patch for a particular region of the unpolished assembly. If the two newer assemblies have identical consensus sequences to each other, and both have a different consensus from the unpolished assembly, retrieve_patchseqs_from_bam.py creates a VCF-formatted correction using the alternative assemblies’ shared sequence as the new allele. We refer to this process as “assembly patching”.

The first assembly we used for assembly patching was a recent assembly run with the Verkko2 assembler and phased with trio data (Table S12). The second assembly was the “lc24 medaka-polished” assembly of HG002 released by Oxford nanopore at the London Calling 2024 conference (Table S12). Both of these assemblies successfully spanned about two thirds of the suspect or problem regions we evaluated, and both confirmed the v1.0.1 assembly consensus in about one third of those cases. In another third, the two assemblies suggested a common correction for v1.0.1 and a patch was applied. The final third of regions spanned by both assemblies revealed differing sequences between the two assemblies, so no correction was made in those cases.

##### Polishing and patching v1.0.1 to create v1.1

In polishing v1.0.1, DeepPolisher ^48^ was run to produce VCF files with suggested corrections that were then filtered and applied directly to the v1.0.1 reference. The DeepPolisher calls were obtained by running DeepPolisher as described in the section “DeepPolisher calls on HG002v1.0.1”, below, and randomly selected DeepPolisher calls were manually curated along with the proposed corrections in other categories. Because curation of these calls revealed a large number of false positives among “DeepPolisher only” calls, only 840 (out of 2,066 total) DeepPolisher calls which agreed with corrections in the DeepVariant call set were applied. The rest of the DeepPolisher calls were clustered into regions which were later considered for potential patching (see below).

As we had done in the previous two rounds, we also ran DeepVariant on multiple sets of reads aligned to the new v1.0.1 assembly, including read sets that had been used in previous rounds, as well as newly available “Q28” ONT and ONT R10 duplex reads (Table S2). The resulting calls were assessed for reliability and accuracy, then used to cross-reference with the DeepPolisher calls as described above, and also used to delineate “exclusion regions” where errors in the v1.0.1 assembly were suspected (see “DeepVariant calls and NIST exclusion regions”).

To ensure that the corrections suggested by DeepPolisher, DeepVariant and our patching process were of high quality, we performed manual curation of randomly selected examples from various sets of corrections that overlapped with each other or were distinct, determining whether subsets of our algorithmic corrections could be confirmed as reliable or not by manual curation. Based on the results of this curation, all corrections were applied from the DeepVariant Element-based calls and all successful assembly patches derived from NIST excluded regions (see the section “DeepVariant calls and NIST exclusion regions”). In addition, any correction seen in two or more of the curated correction sets was applied. Corrections suggested only by DeepPolisher had been found to be unreliable in a majority of the ten examples we curated, but in an attempt to “rescue” possibly valid corrections in the DeepPolisher-only set, we attempted to use the patching method described above to correct the DeepPolisher-only regions, adding a 100 base pair buffer to each side of call locations and running the retrieve_patchseqs_from_bam.py script. ^114^ Of 1,226 DeepPolisher-only corrections, 346 (179 paternal) had a shared alternative consensus in the Verkko2/trio and medaka assemblies to the HG002v1.0.1 assembly, and so a patch was applied, but in another 386 (174 paternal) the two assemblies both had consensus that agreed with the v1.0.1 sequence so no patch was applied. Another 426 (195 paternal) DeepPolisher problem regions had two differing consensus sequences in the two assemblies, so no patch was applied, and finally 68 (25 paternal) regions could not be spanned by both assemblies, making patching impossible.

##### Methods used to discover patching regions

###### DeepPolisher calls on HG002v1.0.1.

40× PacBio HiFi DCv1.2 reads for HG002 were obtained from the HPRC, and aligned to the HG002v1.0.1 diploid assembly using minimap2 ^113^ with parameters -a -x map-hifi –cs –eqx -L -Y -I8g. In order to correct read phasing in long stretches of homozygosity in these alignments, the PHARAOH pipeline ^48^ was run with default parameters, using as input 40× ONT UL >100kb HG002 reads from the HPRC aligned to each haplotype of the HG002v1.0.1 assembly with minimap2 and parameters ‘-a -x map-ont –cs –eqx -L -Y‘. DeepPolisher was run with docker version google/deepconsensus:polisher_v0.0.8_12122023 and model checkpoint 665. Polishing edits were filtered according to the following filters: GQ > 20 for 1bp insertions, GQ > 12 for 1bp deletions, and GQ > 5 for all other edit sizes, as recommended in Mastoras et al. ^48^

##### Github “Issue” submissions

Issues submitted to the HG002-issues GitHub repository, ^141^ with the exception of those in the rDNA arrays and gaps, were also evaluated for potential patching. Of 29 (13 paternal) regions flagged as issues on the GitHub site, the patching process was able to correct 15 (7 paternal) while 10 (4 paternal) had disagreeing consensus in the two assemblies, 3 (1 paternal) remained unspanned by one or both assemblies, and one paternal region had complete agreement between the patch assemblies and v1.0.1.

##### Re-run of Flagger on the T2T-HG002v1.0.1 assembly

We used the Flagger-v0.4.0 pipeline that includes a new module for detecting annotations with coverage biases. ^118^ We were motivated by the fact that some human satellites have exhibited read coverage biases which depend on both the satellite family and the sequencing platform. For example, ONT R10.4.1 Ultra-Long reads have been shown to have upward coverage bias in HSat-1A. The pipeline takes a list of satellite annotations in BED format, compares the median coverage for each annotation to the genome-wide coverage and determines whether it exhibits a coverage bias. This allows Flagger to fit the GMM with an independent set of parameters for each biased annotation, improving the accuracy of misassembly detection in those regions.

To assess assembly quality, we mapped long reads from three sequencing platforms, HiFi Revio (3 flow cells, ∼100× coverage), ONT R10 Duplex (∼80×), and ONT R10 Ultra-Long (∼120×), to the v1.0.1 assembly using Winnowmap (Table S1). We also provided the censat and segdup annotation to the Flagger pipeline for detecting arrays with biased coverage and also stratifying final results. All the bed files used to run Flagger are available in an archived version of the Flagger GitHub repository. ^118^ The WDL for running Flagger-0.4.0 and instructions for running it are available in the software archive for this manuscript. ^114^

##### DeepVariant calls and NIST exclusion regions

The proposed DeepTrio corrections to T2T-HG002v1.0.1 were similar to those used in polishing v0.9, using the same standard insert Element and HiFi reads from the trio aligned separately to each haplotype of HG002v1.0.1 (“all-to-one” alignments), as well as HiFi, ultralong ONT, and duplex ONT aligned to combined haplotypes (“all-to-all” alignments) (Table S2). Several categories of variants were used to generate the “NIST exclusion regions” by adding 50bp to each side of each of the following categories of variants and merging regions within 1000 bp:

1. element DeepTrio calls phased to a haplotype and in a homopolymer or dinucleotide tandem repeat
2. element DeepTrio calls phased to a haplotype supported by any of the long read callsets and not in a homopolymer or dinucleotide tandem repeat
3. element DeepTrio calls not phased to a haplotype supported by any of the long read callsets but not in the opposite haplotype
4. HiFi DeepTrio calls phased to a haplotype and not in a homopolymer or dinucleotide tandem repeat
5. heterozygous or homozygous DeepVariant calls in HiFi and either ONT dataset and <8 bp in size (because larger heterozygous variants tended to be winnowmap2 alignment issues)
6. homozygous DeepVariant calls in HiFi and either ONT dataset
7. heterozygous or homozygous DeepVariant calls in both ONT datasets, not in homopolymers, and <8 bp in size (because larger heterozygous variants tended to be winnowmap alignment issues)

Separate from the exclusions bed file containing these regions, we also attempted to identify *de novo* and mosaic variants and created a separate bed file for these because both are mostly mutations which arose in the cell line and are more likely to vary between cell line batches and not in the iPSC HG002 line. We used TNscope with 300x Illumina WGS from the NIST reference material batches of HG002, HG003, and HG004 aligned separately to each HG002 haplotype. We treated the combined parents as “normal” and HG002 as “tumor”. After curating both small and larger variants, they generally seemed to be true de novos/mosaics if HG002 DP>150 and HG003 and HG004 VAF=0, though many are low VAF (∼3%–5%) in HG002. We separated those that match the other haplotype and lifted them over to the other haplotype since they are either *de novo* or mosaic on the other haplotype. The remaining are likely mosaic but not clear as to which haplotype so we excluded them on both haplotypes. We also used HiFi Revio reads from the trio aligned separately to each haplotype with DeepSomatic in a similar way. Upon curation, these tended to be most likely to be true mosaic or *de novo* variants if they were also in DeepVariant calls from long reads aligned to both haplotypes and not in parental DeepVariant calls or in homopolymers. 50bp were added to each side of these potential mosaic and *de novo* variants, and regions within 1000bp were merged to get 2,686 regions across both haplotypes.

An example of a simplified process for this which might be more generally applicable to polishing other assemblies with Element or Onso reads from a trio, where the only filtering was to select 1 and 2 bp indels in homopolymers or dinucleotide tandem repeats with HG002’s GQ>20 and DP>20 and DP<double the mean coverage (100 in this case), is presented in the software archive for this manuscript. ^114^

#### Total corrections made in three rounds of polishing

VCF files containing all corrections made to HG002v0.7, HG002v0.9, and HG002v1.0.1 (i.e., all of the corrections applied to HG002v0.7 in the three rounds of polishing resulting in HG002v1.1) are available as VCF-formatted files on the HG002-issues GitHub site. ^141^ These files were used to calculate the statistics reported in Table S3 regarding the types of corrections applied in the three rounds of polishing, and correction lengths for the small corrections are displayed in Figure S2.

#### Evaluation of T2T-HG002v1.1

##### Strand-seq assembly evaluation

We used the Strand-seq data generated from HG002^24^ (Table S1) to evaluate the directional and structural contiguity of the HG002v1.1 assembly. Strand-seq data for HG002 are available via the Genome in a Bottle ^2^ and the human pangenomic AWS portal. First, selected high quality Strand-seq libraries (*n*=131) were aligned to the HG002v1.1 assembly using BWA-MEM ^111^ (v0.7.17-r1188), after which reads were sorted by SAMtools ^144^ (v1.15.1) and duplicate reads were marked by sambamba ^125^ (v1.19). To detect putative misassembly breakpoints in T2T-HG002v1.1 we concatenated read alignments across all 131 Strand-seq libraries to create a high coverage Strand-seq read profile across the whole genome. ^145^ We ran breakpointR ^126^ on such high coverage Strand-seq data to detect any recurrent strand-state changes that are indicative of genome misassemblies using runBreakpointr function (set parameters: pairedEndReads = FALSE, windowsize = 50000, binMethod = “size”, genoT = ’binom’, background = 0.1, peakTh = 0.25, minReads = 50). Specifically we were looking for regions where all Strand-seq reads are genotyped as homozygous inverted (‘ww’; ‘HOM’ – all reads mapped in minus orientation) or heterozygous inverted (‘wc’, ‘HET’ – approximately equal number of reads mapped in minus and plus orientation). Homozygous inverted regions are indicative of assembly misorientation while heterozygous inverted regions are mostly marking normal heterozygous inversions in a given individual. In rare cases heterozygous inverted regions might represent assembly chimerism if present at the ends of the assembled contigs (chromosomes).

Figure S3 shows an ideogram in which regions are genotyped as either in complete agreement with the assembly (reference, ‘cc’) or as heterozygous (‘wc’). Heterozygous regions are usually caused by low mappability regions such as short acrocentric arms, centromeres, and repetitive regions. A subset of these mark positions of heterozygous inversions highlighted by arrowheads. We did not observe any region genotyped as homozygous inverted and thus we concluded there is no Strand-seq evidence of misorientation present in these assemblies.

##### k-mer based evaluation

We employed k-mer-based evaluation of assembly consensus quality with Merqury, both to estimate overall error rates and to delineate low-confidence regions of the assembly.^52^ Briefly, Merqury compares *k*-mers present in the assembly’s consensus to a database of k-mers from sequencing data and flags “error k-mers”, i.e., *k*-mers that are present in the assembly but either absent or present in very low numbers in the read k-mers. When using Merqury, it is important to choose an appropriate *k*-mer size, sequencing platform or platforms used to generate the read *k*-mer database, and read coverage, since these decisions will have an impact on the sensitivity and specificity of error detection. For example, use of *k*=21 mers rather than *k*=31 mers tends to result in higher quality scores and fewer flagged error *k*-mers, since 21-mers that are actual errors in the assembly might also be more likely to be present in read datasets due to sequencing error or presence elsewhere in the genome (often on the opposite parental haplotype or within repeats). In addition, some sequencing platforms have biases in coverage, causing them to miss particular sequences, and that can lead to false detection of error *k*-mers where those sequences are (accurately) included in the assembly. We addressed these weaknesses of *k*-mer-based methods by using *k*=31 mers and creating “hybrid” databases from more than one sequencing platform. ^59^

Our final round of evaluation was made using reads from the latest sequencing platforms available. Prior rounds of curation and evaluation had shown PacBio Revio with SPRQ and Element AVITI UltraQ to have the most consistent bases for homopolymers and microsatellite repeats compared to PacBio Revio HiFi (without SPRQ) or Illumina. We used GenomeScope2 and overall genome coverage to evaluate sequencing platforms in an effort to avoid sequencing biases, in particular to find a platform that complements the GA-microsatellite dropouts in PacBio long-reads with minimum sequencing errors.

Using Meryl v1.4.1, k=31 mers were collected from the following read sets for evaluation:

- PacBio SPRQ on Revio, 2 cells sequenced with 30 hours (100x)
- PacBio HiFi on Revio, DeepConsensus v1.2, 2 cells sequenced with 24 hours (70x)
- element AVITI UltraQ Standard 400bp insert, 2×150 (80x)
- Illumina HiSeq2500 2×250, GIAB (60x)
- PacBio Onso 2×150 sequenced on Revio (45x)

Next, we used GenomeScope2 (commit fdeb89178d506c9af2c5d0d103e0135a164889a3) to evaluate the error rate within each set of reads.

$tools/genomescope2.0/genomescope.R -i $pf.k31.hist -k 31 -o $pf.gs2 –fitted_hist

For PacBio SPRQ reads, -l 50 was set to help find the correct kcov in order to obtain a more accurate error rate.

$tools/genomescope2.0/genomescope.R -i $pf.k31.hist -k 31 -o $pf.gs2 –fitted_hist -l 50

To compensate for the known GA-microsatellite dropouts in PacBio long-reads, coverage dropouts were evaluated on HG002v1.1 for short-reads with bedtools genomecov to find overall regions with less than 2 reads aligned. For these short reads, we used the bam files described in the next section “Mapping based evaluation”, and ran bedtools genomecov with the usage:

# regions with coverage < 2x

bedtools genomecov -bga -ibam $pf.dedup.bam | awk ’$4<2’ | bedtools merge -i - > $pf.dedup.cov_lt_2.bed

# exclude rDNA gaps

bedtools subtract -A -a $pf.cov_lt2.bed -b v1.1.gap.bed > $pf.cov_lt2.no_gap.bed

The overall results are summarized in the table below (and in Table S18):

From the GenomeScope2 results, we noted that the SPRQ chemistry had a slightly lower error rate than the HiFi Revio reads without SPRQ. Among short-reads, PacBio Onso data had the smallest error rate but we also found it had the largest sequencing dropouts in GA-enriched regions compared to other short-read platforms, consistent with other PacBio platforms. Therefore, 31-mers from the next best short-read platform, Element Aviti UltraQ, were chosen to act as a complement to SPRQ read-derived 31-mers for the most accurate and complete representation of the genome. A hybrid database was built, of the same type as was used for evaluating T2T-CHM13. ^59^

meryl union-sum [ greater-than 1 AVITI_UltraQ_Std.k31.meryl ] [ greater-than 1 Revio_SPRQ_30hr_PacBio.k31.meryl ] output SPRQ_union_UltraQ.meryl

Subsequently, Merqury (commit ed8c3ba3ea8897d9151a7da8772cd5d83fbce474) was used to re-evaluate HG002v1.1 and all prior assembly versions and benchmark assemblies to obtain a QV score.

$MERQURY/_submit_merqury.sh SPRQ_union_UltraQ.meryl $asm1.fa.gz $asm2.fa.gz $out

The *_only files contain the locations of 31-mers in the v1.1 assembly that are not found in the SPRQ_union_UltraQ.meryl set and represent likely errors in the assembly. A final bed file consisting of these “error kmer” loci was created by merging regions within 5kb of each other, and this file (containing 314 regions with a total of 205,108 base pairs, and 293 regions with a total of 183,143 base pairs outside the rDNA arrays; Table S6) was used in the final issues track (see “Consolidated issues track”).

cat *_only.bed | sort -k1,1V -k2,2n | bedtools merge -d 5000 -i - > v1.1.sprq_elmt_hybrid.error.mrg5kb.bed

For haplotype evaluation, Hamming distance was collected from Merqury results using HG002 Illumina HiSeq2500 2×250 31-mers, along with 31-mers from HiSeq2500 2×250 reads for the parental genomes, HG003 (paternal) and HG004 (maternal).

out=${name}.ilmn

hapmers=$out.hapmers.count

cat $hapmers | awk ’NR>1 { if ($3 > $4) { err=$4; } else { err=$3; } total=$3+$4;\

if (total==0) { print $0 “ \t“err”\t“total”\t0.00”} \

else { print $0 “ \t“err”\t“total”\t”(100*err)/total } }’ > $hapmers.hamming

# Per-haplotype Hamming distance

grep $asm1 $hapmers.hamming | awk -v asm=$asm1 ’{err+=$(NF-2); total+=$(NF-1);} END {print asm“ \t”(100*err)/total}’

if! [[ -z $asm2 ]]; then

grep $asm2 $hapmers.hamming | \

awk -v asm=$asm2 ’{err+=$(NF-2); total+=$(NF-1);} END {print asm“ \t”(100*err)/total}’

cat $hapmers.hamming | \

awk -v asm=“Both” ’{err+=$(NF-2); total+=$(NF-1);} END {print asm“ \t”(100*err)/total}’

fi

After inspection, the majority of discovered switch errors were found to reside in immunoglobulin genes (IG) or TCRs, and therefore were removed from our list of ‘switch errors’. The IG and TCR genes were identified as described below using gene annotations on hg38, lifted to the HG002v1.1 assembly. Excluding these loci, there were 803 regions with a total of 32,014 base pairs, or 794 regions with a total of 31,603 base pairs outside the rDNA arrays.

wget https://s3-us-west-2.amazonaws.com/human-pangenomics/T2T/HG002/assemblies/annotation/VDJregions/HG002.VDJ.lifted_to_v1.1.bed

bedtools subtract -A -a v1.1.hap_switches.bed -b HG002.VDJ.lifted_to_v1.1.bed > v1.1.hap_switches.noVDJ.bed

##### Mapping based evaluation

The following read datasets were mapped to HG002v1.1 using the same parameters as for long and short reads in the previous rounds of polishing:

PacBio reads

- PacBio Revio HiFi DeepConsensus v1.2, 3 cells, one 30hr run and two 24 hr runs
- PacBio Revio with SPRQ, 2 cells, 30 hr

ONT reads

- ONT R10.4 ULK q28 (downloaded from epi2me, “An experimental extremely high-accuracy, ultra-long sequencing kit”, 2023) • ONT R10.4 ULK lc24 (downloaded from epi2me, “Nanopore-only T2T assembly of a human genome”, 2024)

Short-reads

- element AVITI UltraQ Standard 400bp insert, 2×150 (80x)
- Illumina HiSeq2500 2×250, GIAB (60x)

The PacBio reads were filtered to include only primary alignments with no supplementary alignments. The ONT data were filtered to include primary alignments, discarding reads shorter than 10 kbp. In addition, the q28 and lc24 reads were filtered for mapped identity >85% and >95%, respectively, to remove spurious alignments of lower quality reads using filt_bam_len_idy.py (https://github.com/arangrhie/T2T-Polish/blob/master/coverage/filt_bam_len_idy.py).

It should be noted that three of these read datasets (the PacBio Revio with SPRQ reads, the ONT R10.4 ULK “lc24” reads, and the Element AVITI UltraQ reads, were not available until after the release of T2T-HG002v1.1, and therefore provide an independent assessment of the accuracy of the HG002v1.1 assembly.

##### T2T-Polish issues

After filtering the bam files, paf files were re-generated, and issues.sh from the T2T-Polish pipeline (https://github.com/arangrhie/T2T-Polish/blob/master/coverage/issues.sh) was run twice, once with the PacBio alignments and once with the ONT alignments, using the SPRQ+Element hybrid 31-mers described above.

An intersection of the PacBio and ONT issues.bed files was considered as the final set of ‘T2T-Coverage issues’ to avoid coverage biases posed in GA enriched regions of the PacBio reads.

issues.sh hg002v1.1_pb_hifi_sprq.paf PB_HiFi_SPRQ v1.1 HiFi /path/to/pattern

issues.sh hg002v1.1_ont_q28_lc24.paf ONT_Q28_LC24 v1.1 ONT /path/to/pattern

# merge within 1kb

bedtools merge -d 1000 -i hg002v1.1_pb_hifi_sprq.issues.bed > hg002v1.1_pb_hifi_sprq.issues.mrg1k.bed

bedtools merge -d 1000 -i hg002v1.1_ont_q28_lc24.issues.bed > hg002v1.1_ont_q28_lc24.issues.mrg1k.bed

bedtools intersect -u -a hg002v1.1_pb_hifi_sprq.issues.mrg1k.bed -b hg002v1.1_ont_q28_lc24.issues.mrg1k.bed | sort -k1,1V

-k2,2n | grep -v “chrM” > pb_and_ont.issues.mrg.bed

Outside of the rDNA arrays, these T2T-Polish-discovered issues consist of 3 regions with a total of 44,652 base pairs.

##### NucFlag

NucFlag v0.3.3 was applied to the HiFi and ONT read data to generate the first and 2nd most frequent tracks and an output bed file using the following parameters:

nucflag

-i hg002v1.1_pb_hifi_sprq.bam

–output_cov_dir nucflag_freq

nucflag_PB_HiFi_SPRQ.bed

24

-t 24

First and second most frequent allele tracks were generated from the bed output in the nucflag_freq as below and uploaded to the browser:

for seq in $(cut -f1 $sizes)

do

echo $seq

inputs=‘ls $in_dir/${seq}* | sort -k1,1V‘

zcat $inputs | awk -v seq=$seq -v out=$out

’BEGIN { header=“fixedStep chrom=“seq” start=1 step=1”;

print header >> out“_1st.cov.wig”; print header >> out“_2nd.cov.wig” }

{if ($1!=“position”) {print $2 >> out“_1st.cov.wig”; print $3 >> out“_2nd.cov.wig”; } }’

done

wigToBigWig ${out}_1st.cov.wig $sizes ${out}_1st.cov.bw

wigToBigWig ${out}_2nd.cov.wig $sizes ${out}_2nd.cov.bw

For ONT reads, an experimental version of NucFlag branch feature/v1.0 was applied based on communication with the author. This version detects indel mismatches in addition to SNP based mismatches for more sensitive flagging of misassembly.

git clone https://github.com/logsdon-lab/NucFlag.git-brancheature/v1.0NucFlagv1_0-depth1

cd NucFlagv1_0

make venv && make build && make install

source venv/bin/activate

nucflag -i hg002v1.1_ont_q28_lc24.bam

- f /data/Phillippy/projects/HG002_diploid/assemblies/v1.1.fasta

- o nucflag_Q28_LC24.indel.bed

- c /data/Phillippy/tools/NucFlag/NucFlagv1_0/nucflag_ont.toml

- t $SLURM_CPUS_PER_TASK

An intersection of the two missassembly bed files (nucflag_PB_HiFi_SPRQ.bed and nucflag_Q28_LC24.indel.bed) was used for further evaluation.

bedtools intersect -u -a pb_hifi_sprq/nucflag_PB_HiFi_SPRQ.bed -b ont_q28_lc24/nucflag_Q28_LC24.indel.issue.bed | grep -v “chrM” | sort -k1,1V -k2,2n > NucFlag_PB_ONT.indel.issues.bed

bedtools merge -d 100 -i NucFlag_PB_ONT.indel.issues.bed > NucFlag_PB_ONT.indel.issues.mrg100bp.bed

Outside of the rDNA, this intersected set of NucFlag regions had 237 regions with 4,897,713 base pairs.

##### Visualization of short-read alignments

Short-reads were mapped using the T2T-Polish bwa pipeline (https://github.com/arangrhie/T2T-Polish/blob/master/bwa/bwa.sh).

bwa mem -t $cpu $ref $r1 $r2 > $tmp/$out.sam

samtools fixmate -m -@$cpu $out.sam $out.fix.bam

samtools sort -@$cpu -O bam -o $out.bam -T $out.tmp $out.fix.bam

samtools index -@$cpu $out.bam

# mark duplicates and remove them

samtools markdup -r -@$cpu $out.bam $out.dedup.bam

samtools index -@$cpu $out.dedup.bam

# collect primary alignments

samtools view -@$cpu -F0×100 -hb -o $tmp/$out.dedup.pri.bam $out.dedup.bam

These short-read bam files were used, in addition to the merged PacBio and ONT bam files used in the T2T-Polish coverage and NucFlag analysis, for visual inspection of randomly chosen regions using IGV. ^122^ The IGV session files used for this are available via the HG002-issues GitHub repository. ^141^

##### Prior known issues

Issues which had been flagged for patching while making corrections to HG002v1.0.1 but were not correctable (see “Polishing and patching to create v1.1 from v1.0.1”) were lifted from HG002v1.0.1 to HG002v1.1 (see “Chain files to and from the HG002v1.1 assembly”) and included in the track file “hg002v1.1_issues_and_excluded_regions.merged.bed”, which can be viewed and downloaded in the HG002v1.1 browser as the “v1.1 Suspicious Regions” track in the “Assembly and Validation” group. This set of prior known issues, carried over from v1.0.1, was included in the creation of the consolidated “v4” issues track for T2T-HG002v1.1 (see next section) that is displayed in the browser and was used to exclude regions from consideration whenever using T2T-HG002v1.1 as a genome benchmark for the analyses in this manuscript. Briefly, the prior known issue regions fall into the following categories: 1) NIST exclusion regions discovered using DeepTrio, 2) Regions called by DeepPolisher, 3) Uncorrected Sniffles SV calls, and 4) issues submitted through the HG002-issues GitHub repository. ^141^

##### Consolidated Issues Track

Five BED files were integrated to produce the final “v4” issues track for v1.1. The following files described in the above sections were included for merging:

- NucFlag_PB_ONT.indel.issues.mrg100bp.bed
- v1.1.sprq_elmt_hybrid.error.mrg5kb.bed
- v1.1.hap_switches.noVDJ.bed
- pb_and_ont.issues.mrg.bed
- hg002v1.1_issues_and_excluded_regions.merged.bed

Regions in these five files were merged using “bedtools merge -d 1000” into a grand “dirty” BED file of issues, after which each merged regions was assigned to a category by comparison back to the component BED files with bedtools intersect, removing regions from the “dirty” file and assigning them to the largest category with which they overlapped.

After merging and categorization, 3,172 v1.1 regions were flagged as issues, comprising 38.8 Mbp (0.65% of diploid HG002, excluding MT) (Table S6 and Table S7). Note that due to the inclusion of padded extensions and merging across error-free regions, as well as the inclusion of possible false-positive quality issues predicted by the various programs, the total bases flagged in hg002v1.1_issues.v4.bed (available on AWS at https://s3-us-west-2.amazonaws.com/human-pangenomics/T2T/HG002/assemblies/annotation/assemblyissues/hg002v1.1_issues.v4.bed) is likely to be an over-estimate of the error in the v1.1 assembly. In this manner, to provide a reliable benchmark, we erred on the side of caution and flagged any suspicious regions in the assembly.

#### Annotation and browser resources

We have made the two haplotypes of T2T-HG002v1.1 available in the “T2T Genomes” UCSC browser assembly hub as a centralized repository for annotations of the T2T-HG002 assembly (Figure S5).

##### Gene annotation

We generated annotations for the T2T-HG002v1.1 assembly (hereafter HG002v1.1) by mapping genes and transcripts from the T2T-CHM13 annotation onto HG002v1.1. ^127^ The reference annotation used here (JHU RefSeqv110 + Liftoff v5.2) was originally created by mapping the RefSeq (v110) reference annotation of the GRCh38.p14 assembly onto the T2T-CHM13v2.0 assembly using Liftoff ^62^ and Comparative Annotation Toolkit (CAT), ^128^ followed by manual curation. To annotate HG002v1.1, we adopted a two-pass approach to handle unusually challenging regions separately. These regions include the V(D)J gene segments and the ribosomal DNA (rDNA) arrays, which have features that tend to cause errors when trying to map them from one assembly to another. To avoid these errors, we created masked versions of the target genomes, in which the V(D)J regions and the rDNA arrays were replaced with Ns.

To annotate putative V(D)J regions, we used Liftoff to project V(D)J gene annotations from the RefSeq GFF of the T2T-CHM13 assembly onto each haplotype of the v1.0.1 assembly. These annotations were then lifted again to the corresponding haplotypes of HG002v1.1. The set of V(D)J genes included TRA, TRB, TRG, IGL, and IGH. For the IGK locus, where both constant regions were ablated due to the presence of IGKDEL, we defined the locus using the start coordinate of IGKDEL and the end coordinate of IGK. rDNA arrays were identified by aligning a reference 45S rDNA sequence to the assembly using Nucmer with parameters –maxmatch -l 31 -c 100. Only alignments longer than 1,000 bp and with greater than 96% sequence identity were retained, resulting in 51 putative 45S rDNA copies on the MAT chromosomes and 81 on PAT. Note that each 45S rDNA copy encompasses 18S, 5.8S, and 28S rDNA subunits, as it encodes a precursor rRNA that is subsequently processed into mature rRNA subunits.

We treated each haplotype of the HG002v1.1 assembly (MAT and PAT) as a separate haploid genome and processed each independently (including chrX in the MAT run and chrY in the PAT run), because Liftoff is designed to work with haploid assemblies. For the first pass in our process, we used Liftoff to map all features from the reference annotation, excluding additional gene copies, rDNA array rRNA genes, and V(D)J gene segments, onto a masked version of the MAT and PAT assemblies. We ran Liftoff with the parameters -chroms chroms.mat/pat.txt -copies -sc 0.95 -exclude_partial -polish. This initial pass produced annotations of 59,441 genes on MAT and 57,770 genes on PAT, encompassing protein-coding genes, lncRNAs, and other gene types.

The second pass focused solely on mapping the rDNA arrays. In total, 219 rDNA units, comprising 876 rRNA genes, were extracted from the T2T-CHM13 reference annotation. For this mapping step, we also included 7 rRNA gene annotations that occur outside the typical rDNA array structure but still within the acrocentric arms. To ensure that rRNA genes were mapped as complete units, consisting of 45S, 18S, 5.8S, and 28S genes, in that order, we added custom “unit” features to the rDNA annotations. These “unit” features served as parent elements to the four rRNA genes, ensuring that the four genes would be mapped together. To enforce this structural constraint, we used the -f features.txt parameter in Liftoff to restrict annotation to regions where all four rRNA genes co-occur in the correct order. All other Liftoff parameters remained identical to those used in the first pass. This second pass resulted in the annotation of 21 rDNA arrays (84 rRNA genes) on MAT and 28 arrays (112 rRNA genes) on PAT.

The annotations generated by the first and second passes of Liftoff were merged and sorted using gffread ^129^ with parameters -O -F –keep-exon-attrs. Before merging, we used bedtools ^124^ intersect with the -wa -wb flags to confirm that no genes from the first-pass annotation overlapped with those from the second pass. In total, this two-pass process yielded 59,525 genes on MAT and 57,882 genes on PAT.

After running Liftoff, we applied several post-processing steps to improve the quality of the annotated genes and transcripts. One key step involved correcting coding sequence (CDS) features to ensure that their lengths were divisible by three, thereby maintaining proper codon structure. Liftoff does not enforce this constraint, and factors such as minor mapping artifacts, assembly issues, or biological variants (e.g., small insertions or deletions of 1–2 bp) can result in the annotation of CDS features whose lengths are not divisible by three, necessitating trimming. While some of these cases may reflect true frameshifting variants, others arise from technical limitations or ambiguities. We manually inspected a subset of these cases but did not perform a systematic analysis of the underlying sequence contexts; therefore, not all trimmed CDSs should be interpreted as functionally compromised or frameshifted.

To restore the correct codon structure and ensure that the annotated CDS accurately reflects the translated region of a protein-coding gene (with the final three bases corresponding to the last codon), we identified CDS features with lengths not divisible by three and trimmed the excess bases, where bases were trimmed from the end if the first three bases form a valid start codon, and trimmed from the start otherwise. This correction was applied to 586 CDS features on MAT and 534 on PAT. Note that CDS is a transcript-level feature. The sequence responsible for a problematic CDS annotation often spans exon(s) shared by multiple isoforms of the same gene, necessitating trimming across all affected transcripts. Accordingly, the number of genes affected by this trimming procedure is much smaller (e.g., 219 in MAT).

We then extended CDS features that were missing a stop codon to the first in-frame stop codon downstream of the Liftoff-mapped translation termination site. While premature termination codons (PTCs) lead to truncated proteins and potential loss of function (LoF), downstream stop codons that produce elongated proteins are generally considered less deleterious. This procedure resulted in extending 299 CDS features on MAT and 311 on PAT.

To further improve gene annotation, we used Lifton’s extra-copy search submodule ^63^ to run miniprot64, aligning protein sequences from the GRCh38 MANE (v1.4) annotation set to the genome. Protein-to-genome alignments are particularly useful for identifying genes with low nucleotide-level conservation but preserved protein sequences. After generating alignments with miniprot, we compared the resulting annotations to the existing Liftoff-based HG002v1.1 annotations. We filtered out any features that overlapped ≥10% of an existing gene or spanned more than two adjacent gene loci, ensuring that no two genes were annotated in the same genomic location.

Because protein-to-genome alignments only capture CDS regions, we manually added exon, transcript, and gene features to preserve the correct hierarchical annotation structure. If miniprot identified an additional copy of a gene already annotated by Liftoff, we retained the same gene identifier and marked the copy using the “extra_copy_number” attribute. Otherwise, we assigned a new gene identifier. This procedure added 30 genes to MAT and 21 to PAT.

Lastly, to assign gene identifiers, we introduced a T2T-HG002v1.1-specific ID system for all genes and transcripts in the annotation. Unlike reference gene curation efforts such as RefSeq, ^146^ GENCODE, ^147^ or CHESS, ^148^ which sometimes use shared IDs across genomes, this assembly-specific system ensures that each ID refers to a unique object within the HG002v1.1 assembly. Gene IDs follow the format: hg002_[chromosome]_[haplotype]_[gene ordinal number] where the gene ordinal number reflects the gene’s position on a given chromosome counting from left to right; e.g., the 50th gene will have ordinal number 50. Transcript IDs follow the format: [gene_id].[transcript_ordinal_number] where the transcript ordinal number indicates the order of isoforms for a given gene. All features are also tagged with their source gene or transcript ID (i.e., identifiers from the T2T-CHM13 reference annotation), enabling users to track them back to their source in the reference. The current release of the gene annotation excludes the mitochondrial chromosome and V(D)J gene segments.

##### Identification of haplotype-specific genes

We compared haplotype-specific gene copy numbers using HUGO gene symbols to ensure that we were counting only duplicate genes. Genes were considered copies of each other if they shared the same HUGO symbol (Table S8). Using these copy numbers, we identified genes exclusive to only one of the two T2T-HG002v1.1 haplotypes, referred to as haplotype-specific genes. In total, we found 831 distinct genes exclusive to MAT, of which 23 were autosomal, and 78 exclusive to PAT, of which 31 were autosomal. As expected, the majority of haplotype-specific genes were on sex chromosomes.

Since Liftoff/LiftOn, when used to project gene annotations from T2T-CHM13 onto HG002v1.1, is primarily based on sequence similarity, it is possible that close paralogs were annotated in place of a specific gene, potentially resulting in false positive haplotype-specific gene calls. To address this, we aligned the protein sequences encoded by each putative haplotype-specific gene to all proteins on the opposite haplotype using BLASTp. Alignments were filtered using a stringent e-value threshold of 1.0×10^−10^ , ≥ 90% identity, and ≥ 90% query/target coverage. If a gene had more than one match on the other haplotype, it was excluded from the haplotype-specific set.

After filtering, the final list of haplotype-specific genes included 13 MAT-only autosomal genes, and 12 pat-only autosomal genes (Table S12).

##### FIRE analysis of Fiber-seq data for HG002

Using Fiber-seq data generated on HG002 (SRA: SRX24951052), Fiber-seq Inferred Regulatory Elements (FIRE) v0.04 was applied to identify regulatory elements from long-read fiber-seq data using the methods described in Vollger et al.^74^ In brief, FIRE utilizes semi-supervised machine learning to identify MTase-sensitive patches (MSPs) that represent regulatory elements on single chromatin fibers. The methodology utilizes the Mokapot framework and XGBoost algorithms to classify MSPs as likely regulatory elements, assigning each FIRE element an estimated precision value that indicates the probability of being a true regulatory element. This approach provides single-molecule resolution of chromatin accessibility, analogous to a long-read version of DNaseI/ATAC-seq.

Peak calling was performed by identifying FIRE score local maxima with FDR values below a 5% threshold, with peak boundaries determined by the median start and end positions of underlying FIRE elements. False discovery rates were calculated by shuffling fiber locations across the genome and recalculating FIRE scores, defining FDR as the ratio of bases with shuffled scores above a threshold to bases in unshuffled data. The analysis generated multiple track types, including standard FIRE peaks, wide peaks (merged regions within one nucleosome length), coverage tracks showing MSPs, FIREs, and nucleosomes, as well as haplotype-resolved percent accessibility measurements.

##### Repeats and transposable element sequences

Transposable element-derived sequences were annotated using RepeatMasker v4.1.7-p1 ^121^ and a custom library comprising of curated models in Dfam 3.7 ^149^ and those generated as part of the T2T-CHM13, ^26^ ape sex chromosomes, ^34^ and HG002 chromosome Y ^24^ analyses. The aforementioned repeat models were appended to the existing RepeatMasker library as follows:

#Make a directory for the new libraries

mkdir ∼/TEproject/RMplusY_XY_CHM13/

#Copy the RepeatMasker libraries to a new location

cp -r /usr/local/RepeatMasker-4.1.2-p1/Libraries/ ∼/TEproject/RMplusY_XY_CHM13/

#Append the embl file to the existing library

#famdb.py -i ∼/TEproject/RMplusY_XY_CHM13/Libraries/RepeatMaskerlib.h5 append RMplusY_XY_CHM13.embl –name ’RMplusY_XY_CHM13_library’

#Run RepeatMasker with the appended library

RepeatMasker -libdir ∼/TEproject/RMplusY_XY_CHM13/Libraries/RepeatMaskerlib.h5 -s -species v1.1_hg002.fasta -pa 14 -a

This process allows the most sensitive and comprehensive search stages for human repeat detection when using the species flag, but also includes the curated repeats from other primate projects not currently in the Dfam database.

##### Centromeric satellite sequences

Centromeric satellites were annotated using the CenSat workflow. The workflow is written in workflow description language (WDL), and full code plus additional documentation can be accessed via GitHub (https://github.com/kmiga/alphaAnnotation/tree/main).

Alpha satellites were annotated using a modified version of a HumAS-HMMER (https://github.com/enigene/HumAS-HMMER). This workflow annotates alpha satellite monomers from a database of hidden markov models (HMMs). Alpha monomer annotations were merged into summary bins (active array, inactive higher order repeat (HOR), diverged HOR, and monomeric).

Ribosomal arrays were annotated using HMMs based on the first and last 700 bp of the rDNA repeat unit (GenBank: U13369.1) ^150^ as well as two of the rDNA genes; 18S (NCBI Reference Sequence GenBank: XR_007084227.1) and 5.8S (NCBI Reference Sequence GenBank: XR_007084259.1). ^151^ These annotations were then merged to create a complete summary annotation. Scaffolding gaps (sequences of Ns) were annotated using Seqtk gap (https://github.com/lh3/seqtk) to provide complete annotation coverage of rDNA arrays where the rDNA gaps exist. This annotation of T2T-HG002v1.1’s rDNA sequences was used for the locations of the rDNAs for other rDNA analyses in this manuscript (e.g., “Evaluation of HG002v1.1”, and “Fraction of HG002v1.1 that is inaccessible to variant-based benchmarks”).

Classical satellites (HSATII and HSATIII) were annotated using a script previously described in Altemose et al., ^27^ which uses a database of human specific kmers. Annotations were then merged to create a summary annotation over regions where strand switching breaks the annotation.

The final subset of centromeric satellites (HSat1A, HSat1B, bSats and gSats, and smaller species such as SST1, SATR, and ACRO) were annotated using RepeatMasker (Smit, AFA, Hubley, R & Green, P. RepeatMasker Open-4.0. 2013–2015 http://www.repeatmasker.org). Relevant satellite monomers were extracted from the RepeatMasker output and merged to summarize larger arrays. Strand information for all cenSat satellites, where available, were recorded in a separate file hg002v1.1.SatelliteStrandv2.0.bed.

The final CenSat annotation set was created by compiling and automatically curating these satellite annotations described above, including resolving overlaps, filtering out small satellite arrays (<2kb) and merging incomplete annotations. The final step identified the centromere transition (CT) regions, which were determined by merging satellite annotations within 2MB and intersected with the location of the active alpha satellite array. This provided tiled annotation of regions within the centromere transition that don’t have satellite annotations.

##### Segmental duplications

Segmental duplications (SDs) in the HG002v1.1 assembly were annotated as follows: the maternal and paternal haplotype genomes were comprehensively masked in terms of repeats using TRF v.4.1.0, ^131^ RepeatMasker v.4.1.5, ^121^ and Windowmasker (v2.2.22)^132^ with the commands:

asm=hg002v1.1.fasta

trf $asm 2 7 7 80 10 50 2000 -l 30 -h -ngs

RepeatMasker -s -e ncbi -xsmall -species human $asm

windowmasker -mk_counts -mem 16384 -smem 2048 -infmt fasta -sformat obinary -in $asm -out asm.count &&; windowmasker-infmt fasta -ustat asm.count -dust T -outfmt interval -in $asm -out asm.interval

For the maternal haplotype, we concatenated all autosomes from this haplotype with chromosome X and Y to also consider interchromosomal SDs from the two sex chromosomes. Likewise, paternal autosomes and the two sex chromosomes were examined for SDs altogether. Using the repeat-softmasked genomes, SDs were analyzed using SEDEF ^133^ (v1.1). SDs were further filtered for the pairwise sequence identity >90%, length > 1 kbp, and satellite content <70%.

##### Subtelomeric regions

The annotation of human subtelomeres includes the identification and organization of subtelomere repeat elements (SREs). The SREs are subtelomeric DNA segmental duplications, defined as genomic DNA segments greater than 1 kb and greater than 90% similar in nucleotide sequence that are present in two or more subtelomeres. Subtelomeres are defined operationally as the most distal 500kb human DNA segments at each chromosome end. SRE regions contain mosaic patchworks of segmental duplications called paralogy blocks (pblocks), which bear high similarity to discrete segmental duplication segments occurring in multiple subtelomeres. ^152,153^ The identity and organization of paralogy blocks is highly polymorphic and haplotype-specific at many subtelomeres, ^154^ and may play a role in cis-regulation of single telomere and haplotype-specific telomere lengths ^155,156^ ). Representative pblock sequences from GRCh38 were used to identify most pblocks from HG002v1.1 SRE regions, but eleven new SRE pblocks were also identified in the HG002v1.1 assemblies.

Representative sequences for existing GRCh38-derived pblocks were masked (with Repeat Masker (Smit, AFA, Hubley, R & Green, P. RepeatMasker Open-4.0. 2013–2015 http://www.repeatmasker.org) and Tandem Repeats Finder ^131^ software run under default parameters) and then aligned to the reference sequence using BLASTn v.2.13.0+, ^134^ requiring a minimum of 90% identity and 100 bp alignment length. Groups of alignments (localized, ordered, co-directional, greater than 1kb chain-length) form a mapping location of a pblock to the reference. If a pblock aligned only partially with a given segment of the reference sequence, then if possible, it is extended by pairwise alignment of the unmasked p-block sequence to the flanking segment(s) of the reference. If neighboring pblocks are overlapping, then the one with the highest percent identity to the reference in the overlapping area is selected.

After all GRCh38-derived pblocks were mapped to HG002v1.1, remaining subtelomere regions of HG002v1.1 were investigated for potential new pblocks. These regions included gaps greater than 1kb between existing mapped pblocks within a subtelomere, and the subtelomere reference areas towards the centromeric side of the subtelomere region (<500kb), where no existing pblock mapped. The investigation was carried out by masking (with Repeat Masker and Tandem Repeats Finder) these candidate pblock regions and aligning them to the complete HG002v1.1 reference. If a segment of a subtelomere maps to multiple subtelomere areas (<500 kb from telomeres), it was annotated as a valid new subtelomeric pblock.

##### Representation of additional HG002 rDNA units

The rDNA arrays are the most complex regions to assemble in the human genome and, as a result, are the only gaps remaining in the HG002v1.1 reference. To provide a representation of additional copies of HG002’s rDNA to the benchmark, we’ve run ribotin, a tool developed specifically for rDNA array assembly, ^47^ develop branch, commit d8a73739d5f7a5de3d27904a18e53d5bbfdf14e9)

ribotin-ref -t 10 -r rDNA.KY962518.1.fasta -i m84005_220827_014912_s1.hifi_reads.fastq.gz -i

m84005_220919_232112_s2.hifi_reads.fastq.gz -i m84011_220902_175841_s1.hifi_reads.fastq.gz –nano all_pass.vhg002v1.-fastq.gz –approx-morphsize 45000 -o ribo_epime/

Ribotin outputs sequences which represent the most frequent rDNA units in the sample and a graph that represents all different rDNA units present in the sample with their relative order. The rDNA morph consensus sequences and the graph showing their relative order are available at https://s3-us-west-2.amazonaws.com/human-pangenomics/index.html?prefix=T2T/HG002/assemblies/annotation/rdna/hg002_rdnamorphs_v0.1/.

##### Creation of a T2T-HG002v1.1 ideogram track

Unless otherwise noted, all input files, along with the conversion tools bigBedToBed and liftOver, were obtained from the UCSC Genome Browser. ^120^ The T2T-chm13v2.0 (hs1) cytoBandMapped track file was obtained from https://hgdownload.soe.ucsc.edu/gbdb/hs1/cytoBandMapped/ and converted to bed format. Cytoband coordinates were first lifted over to hg002v1.1.mat and hg002v1.1.pat separately using chain files CHM13v2.0_to_hg002v1.1.mat.chain and CHM13v2.0_to_hg002v1.1.mat.chain (see the section “Chain files to and from the HG002v1.1 assembly”). The CenSat annotation track (see “Centromeric satellite sequences”) was obtained from https://github.com/hloucks/CenSatData/tree/main/HG002/v1.1. The Perl script Refine_CytoBand_Liftover.pl ^135^ was generated to extract centromere, telomere, rDNA, and heterochromatin boundary information from the CenSat track and fasta index files, then to use this information to adjust the boundaries of these regions in the lifted over cytoband tracks, as follows. Centromere band boundaries were placed at the most distal ends of the “active_hor” array annotation (or split HOR arrays, e.g. on chr3 and 4) on each chromosome. The p to q transition was placed at the midpoint between these centromere band boundaries. Centromere-adjacent band coordinates were adjusted to be contiguous. The last band on each chromosome was adjusted to have its end position match the chromosome end position. rDNA stalk band coordinates were adjusted to match the boundaries of the rDNA arrays. The 1q12, 9q12, and Yq12 boundaries were adjusted to the ends of the large HSat2/3 arrays in those regions, and adjacent band coordinates were adjusted accordingly. For any remaining bands with boundaries that failed to lift over, their coordinates were fixed to preserve the relative sizes of that band and its neighboring bands relative to their sizes in T2T-chm13v2.0. For validation, non-gap band sizes were confirmed to be preserved after liftover, and adjacent band coordinates were checked to be contiguous.

##### Heterozygous variants and heterozygosity

Comparison of the maternal and paternal haplotypes of autosomes and the pseudoautosomal (PAR) regions of the sex chromosomes is sensitive to the methods used to align homologous sequences to each other as well as to the decisions made when filtering alignments of repetitive regions. In the absence of prior work prescribing methods for this, heterozygous variants and estimates of heterozygosity were computed as follows:

A FASTA file of all maternal HG002v1.1 autosome sequences was aligned to the corresponding file of paternal sequences with minimap2 version 2.26 with the command:

minimap2 -a -t2 -x asm5 v1.1.pataut.fasta.gz v1.1.mataut.fasta.gz | samtools view -O BAM |

samtools sort –threads 2 -T v1.1.mataut_vs_v1.1.pataut.tmp -O bam -o v1.1.mataut_vs_v1.1.pataut.sort.bam

A second “reverse” BAM file was created using the opposite alignment (paternal chromosomes to maternal). The two BAM files were then passed to the GQC script “gethets” ^114^ to produce a BED file of heterozygous variant locations and a heatmap-colored track of windowed variant counts.

gethets –bam1 v1.1.mataut_vs_v1.1.pataut.sort.bam –bam2 v1.1.pataut_vs_v1.1.mataut.sort.bam –ref1 v1.1.pataut.fasta.gz –ref2 v1.1.mataut.fasta.gz –prefix v1.1.hets.non1to1.200k –non1to1 –windowsize 200000 –heatmap

In addition, the sex chromosomes were compared using the same minimap2 parameters, and the two directed BAM files were again passed to the gethets program with the same parameters to find heterozygous positions and calculate heterozygosity in the pseudoautosomal regions.

The gethets script filters the alignments passed to it as follows.

1. for each of the two directed BAM files (maternal to paternal, paternal to maternal):
  - include only alignments that are designated as “primary”
  - alignments must cover at least 10,000 bp along the reference (target)
2. Find pairs of corresponding alignments. For each included alignment in the first BAM:
  - if a reciprocal matching alignment in the second BAM exists (for which the target has the same start and end coordinates as the alignment’s query coordinates, and the query has the same start and end coordinates as the target start and end, pair the two alignments
  - If no reciprocal alignment exists in the second BAM file, examine all alignments in the second BAM file for which the covered query region intersects the first alignment’s target region. If the intersecting target region’s endpoints in the first file align to the same opposite haplotype positions in both the first and the second BAM file, use the sub-alignments in the intersecting portion as corresponding alignments
3. For all pairs of corresponding alignments, tally heterozygotes as any mismatch, insertion, or deletion within the alignment’s CIGAR string. In addition, calculate the number of these heterozygous positions in windows sized according to the parameter passed to the gethets program.

A BED track reporting all heterozygous positions between the maternal and paternal haplotypes in HG002v1.1, as well as a windowed BED file with heatmap-colored heterozygosity values is displayed and available for download in the UCSC browser assembly hub for HG002v1.1.

##### Chain files to and from the HG002v1.1 assembly

Chain files for lifting coordinates to and from the HG002v1.1 assembly were created using the “nf-LO” pipeline described in Rhie et al., Nature 2023. ^24^ Briefly, we used nextflow to run nf-LO^136^ between the HG002v1.1 genome and a haploid target genome (e.g., GRCh38 or CHM13v2.0), then split the chains at all locations where there were unaligned segments longer than 1kbp or gaps longer than 10kbp. Only alignments between homologous chromosomes were retained. The package rustybam^137^ was then used to trim overlapping portions of the chains, resulting in one-to-one alignments. The package chaintools ^138^ was used to invert chains to obtain chain files for the reverse direction.

Chain files used to lift coordinates to and from HG002v1.1 for this manuscript are displayed and available for download in the UCSC browser assembly hub for HG002v1.1.

nextflow run nf-LO/main.nf –source $SOURCEFASTA –target $TARGETFASTA –outdir . -profile local –aligner minimap2 –max_cpus 2 –max_memory 64Gb -resume python chaintools/split.py -c ./chainnet/liftover.chain -o $PREFIX-split.chain python chain-tools/to_paf.py -c $PREFIX-split.chain -t $SOURCEFASTA -q $TARGETFASTA -o $PREFIX-split.paf

awk ’{short1=$1; short2=$6; gsub(“_MATERNAL”, “”, short2); gsub(“_PATERNAL”, “”, short2); if(short1==short2) {print}}’

$PREFIX-split.paf > $PREFIX-samechr-split.paf

cat $PREFIX-samechr-split.paf | rb break-paf –max-size 10000 | rb trim-paf -r | rb invert | rb trim-paf -r | rb invert > $PREFIX.paf

paf2chain -i $PREFIX.paf > $PREFIX.chain

python chaintools/invert.py -c $PREFIX.chain -o $PREFIX.inverted.chain

##### Regions inaccessible to variant-based benchmarks

To determine the fraction of the non-rDNA HG002 genome that was not included in the GIAB v4.2.1 read-based small variant benchmark, we lifted the “high confidence regions” of v4.2.1 (available at https://ftp-trace.ncbi.nlm.nih.gov/giab/ftp/release/AshkenazimTrio/HG002_NA24385_son/NISTv4.2.1/GRCh38/HG002_GRCh38_1_22_v4.2.1_benchmark_noinconsistent.bed) onto each haplotype of the HG002v1.1 assembly separately using the GRCh38 to HG002v1.1 haplotype chain files described in “Creation of chain files to and from the HG002v1.1 assembly”.

The total bases in the lifted regions and in the v1.1 genome are presented in Table S17. In particular, of the 5,965,910,578 base pairs of non-rDNA sequence in the T2T-HG002v1.1 assembly, only 5,081,210,845 base pairs, or 85.2%, are covered by lifted “high confidence” regions of the GIAB v4.2.1 variant benchmark. In contrast, the “non-excluded” regions of the T2T-HG002v1.1 assembly cover 5,960,629,742, or 99.9%, of the 5,965,910,578 base pairs of the non-rDNA portions of T2T-HG002v1.1 (Table S6).

##### GIAB missed regions in different types of regions

We loaded chromosome sizes and the lifted-over GIAB confident regions into separate GRanges objects and used the setdiff function from the GenomicRanges package to identify regions of the HG002 autosomes missing from the GIAB confident set. To assess whether these missing bases overlapped biologically functional elements, we imported gene annotations (HG002v110.JHU.v0.5.- withheader.gff), coding sequence (CDS) features, and cenSat annotations (see “Gene annotation”, above) into GRanges objects and intersected them with the missing regions. Set intersections were visualized using the eulerr package in R, where values were converted into named vectors and fitted to Euler diagrams, and the final venn diagram is shown in Figure 1B.

#### Use of HG002v1.1 as a genome benchmark

##### Alignment as a precursor to evaluation

When comparing test assemblies and sequencing reads to a genome benchmark like T2T-HG002v1.1, a critical first step is alignment of the test sequence to the diploid benchmark. Since the test sequences are generated from the same sample as the benchmark, correct sequences should have at least one exact match in the benchmark, but alignments to large regions of homozygosity might still be assigned a zero mapping quality (MQ) due to the existence of two equally-scored alignments. Aligners like minimap2 ^113^ will assign to one of the two haplotypes randomly. In these cases, measures of coverage and discrepancy levels will be the same regardless of the haplotype assignment. For this reason, GQC does not filter alignments for mapping quality when evaluating assemblies or sequencing reads.

By default, GQC uses minimap2 alignments of assembly contigs to their locally phased haplotype, since comparison of minimap2, winnowmap2, and wfmash did not show a significant effect on GQC statistics and minimap2 has considerably lower CPU and memory requirements.

##### Evaluation of test assemblies using GQC

The availability of a near-perfect diploid benchmark for HG002 allows users to evaluate haploid and diploid genome assemblies by comparing them to the benchmark. To facilitate this and other comparisons, we developed a software tool called GQC, and archived the version used for this manuscript’s analyses in the manuscript’s software repository. ^114^ GQC is implemented in python, and includes libraries for aligning sequences and parsing the alignments to determine continuity and consensus accuracy at a local and genome-wide level.

###### Pre-phasing diploid assemblies with GQC.

To report quality statistics about assemblies, GQC first pre-phases the assembly scaffolds into maternal and paternal regions (“phase blocks”) based on the presence of haplotype-specific kmers present in only one haplotype of the v1.1 benchmark (“assembly-hapmers”). This is done to find phase switches, i.e., places in test assembly scaffolds where the consensus changes from maternal to paternal sequence, or vice-versa. Phase blocks are calculated first by calculating and then mapping the assembly-hapmers to the test assembly using FASTK v1.1 (Gene Myers, https://github.com/thegenemyers/FASTK, accessed February 14, 2025 and archived in the software archive for this manuscript. ^114^ The assembly-hapmer locations are then used as the observables of a two-state hidden Markov model (“HMM”) and the Viterbi algorithm is then used to predict the most probable underlying benchmark haplotype “state” of the assembly at each locus. The predicted states are then used to write a BED-formatted file of phase block regions within each scaffold.

Briefly, the hidden Markov model has a state *S*_*i*_ at each assembly-hapmer position *i*, where *S*_*i*_ *={‘mat’, ‘pat’}* is the haplotype of the position’s phase block and is maternal or paternal. In addition, a state *S*_*i*_ “emits” an observable *O*_*i*_ that is also one of “mat” or “pat” with probabilities 1 − *α* (if the state and the observable represent the same haplotype) or *α* (if the state emits an observable from the opposite haplotype). To allow for phase changes, the model allows the underlying state *S*_*i*_ to alter its value between adjacent assembly-hapmer positions with probability *β* (for a switch to the opposite haplotype state) and correspondingly, the model assigns a probability of 1 − *β* for *S*_*i +1*_ to remain in the same haplotype state at the following marker. By examining the size of resulting haplotype blocks and the rate of opposite haplotype markers within blocks, we chose *α* = 0:05 and *β* = 0:01 as default values for GQC to pre-phase the assembly scaffolds it evaluates. When adjacent assembly-hapmers have a change in state in the resulting state chain, the position exactly between the two hapmer positions is chosen as the boundary between the two phase blocks.

After determining the scaffold phase blocks, GQC uses minimap2 to align all assembly scaffolds separately to the maternal part of the v1.1 benchmark and to the paternal part, then considers phased regions of the assembly one by one, using only the alignments of that region to their same-haplotype region based on the pre-phasing. Since the alignments to the single-haplotype assembly frequently extend beyond the boundaries of the predicted assembly phase blocks, only the portion of each alignment that lies within the phase block is considered in the evaluation (see “Evaluation of assembly accuracy within alignments to the benchmark”).

###### Evaluation of assembly’s long-range continuity.

In addition to reporting on a test assembly’s standard continuity metrics (contig and scaffold N50/L50, NG50/LG50, and auNG), GQC calculates statistics with regard to the portions of the assembly’s scaffolds that are aligned to the genome benchmark. While N50, NG50, etc. are calculated based on assembly contig and/or scaffold lengths, the aligned statistics NGA50/LGA50, etc. are dependent on the alignments themselves, which are determined using minimap2 with the “asm5” preset by default. Because minimap2 can sometimes be unpredictable with regard to how large an indel it will incorporate into its alignments, GQC breaks alignments at the locations of indels which are 10,000 base pairs or larger in size, and joins consecutive same-strand alignments if the endpoint of the first is closer than 10,000 base pairs to the starting point of the next along both the test assembly sequence and the benchmark. Once alignments are split and combined in this way, NGA, LGA, and auNGA statistics are calculated from the lengths in a manner similar to their counterparts, and breaks in alignments are reported in BED file format. An example of an insertion error detected by GQC is illustrated with an SVbyEye plot^139^ and a ModDotPlot ^119^ alignment plot in Figure S6.

###### Evaluation of assembly accuracy within alignments to the benchmark.

Within each region of the assembly’s phased scaffolds, the alignment to the haplotype matching the region’s phase block is used to tally the assembly’s errors. Any substitution, insertion, or deletion within the alignment is listed in a BED-formatted output file of errors (and optionally, a VCF-formatted file), and classified as either a “phasing error” when the assembly allele matches the benchmark sequence on the opposite haplotype, or a “consensus error” if the assembly allele doesn’t match either benchmark haplotype. The number of errors of each type are used to calculate Phred-scaled quality values reported in the program’s general statistics output file.

###### Evaluation of mononucleotide run accuracy.

In addition to being listed as errors in the consensus analysis, mononucleotide errors are evaluated specifically for the program’s mononucleotide accuracy statistics. The HG002v1.1 assembly has 1,990,653 mononucleotide runs of length ten or greater, and for each of these runs that are included within a same-haplotype alignment of the phased assembly scaffolds to the benchmark, runs are classified as either correct (the complete run is in the scaffold and has the same number of bases as the benchmark), wrong haplotype (the complete run is in the scaffold but has a different number of bases than the matching haplotype, but the same number as the benchmark’s opposite haplotype), wrong length (the complete run is in the scaffold but has a different number of bases than either haplotype of the benchmark), or erroneous (the run contains other sequence beside the single base within the mononucleotide sequence).

###### Assemblies used for comparison and evaluation with HG002v1.1.

We used other high quality assemblies of HG002 for validation of HG002v1.1 as well as for demonstration of HG002v1.1’s value as a genome benchmark. Our motivation in selecting this particular group of assemblies was to evaluate using the benchmark the improvement of assemblies over several years’ time. Details of these assemblies’ availability for download and citations of manuscripts describing them are listed in Table S12. Briefly, the HG002 assemblies used in this work’s evaluation were:

1. The “Ash1v2.0” haploid assembly ^53^ contigs were filtered to include only the main chromosomes:
  for Chrom in ‘awk -F “ \t” ’{print $1}’ Ash1_v2.0.fa.fai | grep ’chr’ |
  grep -v ’random’ | sort‘; do samtools faidx Ash1_v2.0.fa $chrom >> Ash1_v2.0.mainchroms.fasta; done
  This assembly was generated using 71x coverage of Illumina 250 base pair reads, 23x coverage of Oxford Nanopore reads averaging over 33,000 base pairs in length, and 29x coverage of HiFi 10,000 base pair reads. These data were assembled using the MaSuRCA assembler ^157^ into a single haplotype that is a mosaic of the maternally and paternally-inherited chromosomes of HG002.
2. The hifiasm diploid assembly published in Cheng et al. (2021)^54^ was created using the hifiasm assembler v0.12 run on 40x of coverage in PacBio SequelII HiFi reads from 15kb and 20kb CCS libraries.
3. The “release 1” HPRC diploid assembly was downloaded from the HPRC AWS site (s3://human-pangenomics/working/HPRC_PLUS/HG002/assemblies/year1_f1_assembly_v2_genbank) ^10,41^ This assembly was created using the hifiasm assembler ^54^ using data similar to other samples that were part of the HPRC project’s first data release. Specifically, PacBio HiFi Sequel II reads were assembled using Trio-Hifiasm v.0.14.1, using parental Illumina reads to perform k-mer based phasing of contigs.
4. The verkko diploid assembly published in Rautiainen et al. (2023) ^28^ was the “full-coverage verkko+trio” assembly listed in Table 2, and used 105x coverage of PacBio HiFi Sequel II reads called with DeepConsensus and 85x coverage of Oxford Nanopore Ultra-long reads.
5. The diploid assembly “lc24_medaka_6b4” was announced at Oxford Nanopore’s 2024 London Calling meeting. This is a verkko2 assembly run entirely on ONT reads from multiple separate sequencing kits, including the “6b4” chemistry kit designed to improve the accuracy of homopolymer runs.

##### Evaluation of read accuracy using GQC

###### Datasets used to demonstrate read accuracy benchmarking.

Four read datasets were included in the read benchmarking results presented in this manuscript. Information on where they were obtained is in Table S12. GQC readbench results, as well as AWS links to the aligned BAM files used to generate them, are in Table S15. The first read set evaluated was the high accuracy, ultra-long Oxford nanopore sequence dataset presented by Epi2Me at the Nanopore Community meeting in December 2023). The announcement of this dataset describes the dataset as:

Ultra-long libraries of native DNA from GM24385 (HG002) were prepared using a modified Ultra-Long DNA Sequencing Kit V14 motor protein and experimental high-accuracy run conditions. Sequencing was performed on a PromethION instrument to obtain 125 Gbp of sequencing data passing quality filters (read Q-score > Q10, ), with a read length N50 of 91 kbp. This data was base-called using a bespoke dorado model to yield a median accuracy of Q26.4.

The second read set used in the read benchmarking analysis were HiFi reads sequenced by Pacific Biosystems in May 2024 with 24 hour movies on a Revio ICS 13 flowcell, then called with DeepConsensus v1.2. The third read set was sequenced by Element Biosciences using their UltraQ (Q50) chemistry with a 400 base pair insert size, and the fourth set was a dataset of PCR-free whole genome sequencing reads, sequenced in 2020 on the Illumina NovaSeq platform, and described in Baid et al. ^158^

The “readbench” command in the GQC package ^114^ allows users to evaluate aligned sequence reads for substitution and insertion/ deletion discordance rates.

###### Calculation of substitution and small insertion/deletion discrepancies.

From a user-supplied BAM file, GQC readbench reports all discrepancies (single nucleotide substitutions and indels) within alignments of reads to the benchmark genome. If the allele displayed on a read matches the opposite haplotype’s allele at a heterozygous site of HG002, the error is tagged as a “phasing” error.

###### Coverage assessment.

Based on a BAM file’s coverage and read lengths, GQC readbench’s “–arrivalratecoverage” option sets a bin size estimated to result in a mean 1,000 read starts per bin, and then reports the number of read starts in each non-overlapping bin of that size which is fully included within the non-excluded regions of the HG002v1.1 genome benchmark. These bin arrival rates can then be used to plot cumulative coverage curves like the ones in Figure 4E of this manuscript. In addition, GQC’s “–bincoverage” option reports the average coverage of primary read alignments across bins of a user-specified size as well as the GC content within each bin in BED format, using a cumulative sum operation on read start and stop “events” for efficiency, following the methods used by the program mosdepth. ^140^ These values, printed in BED format, can be used to make plots like the one in Figure 4F of this manuscript.

###### Assessment of short tandem repeat accuracy.

For regions of the diploid benchmark that are annotated as homopolymer, dinucleotide, trinucleotide, and tetranucleotide runs, GQC readbench assesses the accuracy of a sequence read for that run as follows:

1. First it determines the read positions aligned to the five bases immediately flanking the short tandem repeat (STR) on each side. If there aren’t aligning bases due either to deletion or alignment clipping, it extends the STR-aligned read sequence to include adjacent bases so there are five bases on each side in addition to the STR sequence.
2. If the sequence within the STR is an exact repeat of the motif, it counts the number of bases within the STR sequence and compares it to the benchmark run length. If it matches, and the five flanking bases on each side match the benchmark’s flanking bases, the read is counted as “CORRECT”. If the run length matches but there are differences in the flanking bases, the read is counted as “FLANKERROR”.
3. If the sequence within the STR is an exact repeat of the motif but its length is different from the benchmark that it is aligned to, and the opposite haplotype of the benchmark is a run of the same length as the read, it counts the read as a “HET” error. If the read STR length is different but doesn’t match the opposite benchmark haplotype, it is counted as a “LENGTHERROR”.

GQC readbench writes files with the read classification for each assessed STR for each assessed read. It then creates a plot of STR accuracy as a function of run length and a histogram plot showing the frequencies of different length errors.

###### Evaluation of base quality scores.

If the user-supplied BAM file contains base quality scores, GQC readbench will tally binned accurate bases and errors (reporting substitutions and indels separately) by quality score as it processes alignments. It reports these counts, along with an effective Phred-scaled quality score for each bin, in a separate output file. QV bin accuracy tallies for the various read data sets were used to generate the plots in Figure 4D.

To assign a base quality score to an error, GQC uses the caller-reported QV score at the exact base of a single nucleotide substitution error. For non-ambiguous deletion errors, the base quality score of the base immediately preceding the deleted sequence is assigned to the error, while in insertions or ambiguously placed deletions (e.g., deletion of one base from a homopolymer run), the set of all caller-reported QV scores at positions within the expanded event are sorted, and if there are an odd number, the median of the QV values is assigned to the error, while for an even number *n*, the “lower middle value”, or *n/2-*th lowest quality score, is selected. This procedure is designed to only assign (and thus count) QV values as errors if there is a corresponding read base being counted with that score in the total counts used as the denominator when calculating the observed accuracy of read bases with a given callerassigned QV score.

To explore the possibility that the poor calibration of observed versus assigned base qualities in Figure 4D for the HiFi Revio and ONT Q28 reads is due to the difficulty in assigning base quality scores to indel errors, we created the same plot calculating the observed QV scores using only the substitution errors (Figure S7). For the ONT Q28 reads, this “substitutions only” observed QV versus reported QV curve tracked the *x=y* line more closely, with observed QV slightly higher than reported QV for all binned values, while the HiFi Revio “substitutions only” observed QV scores were also higher than the caller-reported QV scores for all bins, but with a much larger difference. For example, the observed “substitutions only” QV for ONT Q28 bases with reported QV of 28 is 28.66, while for HiFi Revio bases with reported QV of 28, it is 36.54.

##### Evaluation of variant call sets

###### Converting gVCF files to VCF files with high confidence region BED files.

Variant callers like DeepVariant output gVCF files containing entries with an “END=” value in the INFO field and a “0/0” genotype with a GQ score for the sample for regions that DeepVariant is calling as a match to the reference. In addition, entries with a “PASS” in the filter field report a genotype and GQ score at variant positions similar to those in a traditional VCF file.

For the non-variant gVCF entries that include an “END=” value, we included the region beginning at the entry’s position and ending at its END value in the high confidence region BED file. For passing genotype calls without END values, we also included the region from the entry’s position with length equal to the length of the reported REF allele as a high confidence region. Regions with a genotype of “./.” were **not** included in the high confidence region BED file.

The gVCFs used in this analysis are publicly available from the NCBI trace archive (Table S13).

The commands used to create the high confidence BED files are:

# Homozygous reference sites with genotype quality at least $MINQ:

bcftools query -i ’GT=“RR” && INFO/END!=“.”’ -f ’[%CHROM\t%POS0\t%INFO/END\t%GQ\n]’ $GVCF | awk -F “ \t” ’$4>=ENVIRON[“MINQ”] {OFS=“\t”; print $1, $2, $3}’ | awk -F “ \t” ’$2>=0 {print} $2<0 {OFS=“\t”; print $1, 0, $3}’ > $HIGHCONFBED

# add passing variant/genotype call regions that have GQ at least $MINQ:

bcftools query -i ’INFO/END=“.”’ -f ’[%CHROM\t%POS0\t%END\t%GQ\n]’ $GVCF | awk -F“\t” ’$4>=ENVIRON[“MINQ”]

{OFS=“\t”; print $1, $2, $3}’ | awk -F“\t” ’$2>=0 {print} $2<0 {OFS=“\t”; print $1, 0, $3}’ >> $HIGHCONFBED

High confidence regions were sorted and merged to remove overlaps/redundancy:

sort -k1,1 -k2,2n -k3,3n $HIGHCONFBED | bedtools merge -i - > $HCMERGEBED

The gVCF file’s header and all entries with non-reference genotypes that are also not “./.” are included in the VCF variant file that is subsequently used to alter the genome reference file to create a “variant-constructed genome”.

The command used to create the VCF file of variants is:

gunzip -c $GVCF | awk -F “ \t” ’$1∼/ ^#/ || ($9∼/ ĜT/ && $10!∼/ ^0\/0/ && $10!∼/ ^\.\/\./) {print}’ | awk -F “ :” ’$1∼/#/ || $2==“GQ” && $8>=ENVIRON[“MINQ”] {print}’ |

bgzip -c > $VARIANTVCF

###### Phasing variant call sets.

Because the variant call sets were unphased, we used HiFi reads (Table S12) to phase the VCF-formatted variants created in the previous step with HapCUT2. ^91^ This was to ensure that the variant-constructed genome would represent two single haplotypes, at least on a local level.

HapCUT2 was run using the following commands:

extractHAIRS –pacbio 1 –realign_variants 1 –ref $REF –bam $BAM –VCF $VARIANTVCF –out $PREFIX HAPCUT2 –fragments $PREFIX –VCF $VARIANTVCF –output $PREFIX.haplotype

###### Creating and benchmarking a variant-constructed genome.

To create two separate haplotypes for the two VCF alleles at autosomal sites, the phased diploid VCF file was split into two haploid VCF files using the following commands:

gunzip -c $VCF | grep -v ’RefCall’ | awk -F “ \t” ’$1!∼/#/ && $1!∼/chrY/ {$NF=gensub(/([0–9]+)([|/]).*/,“\\1\\2\\1”,“g”, $NF)} {OFS=“\t”; print}’ | bgzip -c > $HAP1VCF

gunzip -c $VCF | grep -v ’RefCall’ | awk -F “ \t” ’$1!∼/#/ && $1!∼/chrX/ {$NF=gensub(/([0–9]+)([|/])([0–9]+).*/,“\\3\\2\\3”,“g”, $NF)} {OFS=“\t”; print}’ | bgzip -c > $HAP2VCF

These haploid VCF files were used with bcftools consensus to create two FASTA-formatted haploid genome files from the reference file against which the variants were called:

bcftools consensus -c $CHAIN1 -f $REF -s $SAMPLE $HAP1VCF > $NEWREFPREF.hap1.fasta

bcftools consensus -c $CHAIN2 -f $REF -s $SAMPLE $HAP2VCF > $NEWREFPREF.hap2.fasta

and the resulting chain files (produced because the “-c” option was used with bcftools consensus) were used to create BED-formatted files for the high confidence regions against each of the new haploid genomes:

liftOver $HQBED $CHAIN1 $NEWBED1 $UNMAPPEDBED1

liftOver $HQBED $CHAIN2 $NEWBED2 $UNMAPPEDBED2

Finally, the high-confidence BED files were used to mask their respective genome FASTA files using “bedtools maskfasta”:

bedtools subtract -a $NEWREFPREF.genome.hap1.bed -b $NEWBED1 > $LQBED1

bedtools subtract -a $NEWREFPREF.genome.hap2.bed -b $NEWBED2 > $LQBED2

bedtools maskfasta -fi $NEWREFPREF.hap1.fasta -bed $LQBED1 -fo $NEWREFPREF.hap1.highconf.fasta

bedtools maskfasta -fi $NEWREFPREF.hap2.fasta -bed $LQBED2 -fo $NEWREFPREF.hap2.highconf.fasta

These variant-constructed genome haplotypes ($NEWREFPREF.hap1.highconf.fasta and $NEWREFPREF.hap2.highconf.fasta) were then compared to the hg002v1.1 benchmark and evaluated using the methods described in the section “Evaluating genome assemblies with GQC”.

To obtain the values plotted in Figures S8A–S8D and for calculating the figures reported in the main paper, the following shell commands were run on the GQC output files:

for file in ‘ls GRCh38_GQC/GRCh38_Revio_DV_HiFiPhased.mingq[1234]0/GRCh38_*mingq[1234]0/*.benchcovered.v1.1.bed CHM13_GQC/CHM13_Revio_DV_HiFiPhased.mingq[1234]0/CHM13_*mingq[1234]0/*.benchcovered.v1.1.bed‘; do

export GENSTATFILE=‘echo $file | sed \ ’s/.benchcovered.v1.1.bed/.generalstats.txt/’‘

export QUAL=‘echo $file | sed ’s/.*mingq//’ | sed ’s:\ ..*::’‘

export REF=‘echo $file | sed ’s/ _.*//’‘

export COVERED=‘awk -F “ \t” ’{sum += $3–$2} END {print sum}’ $file‘

export TOTERRORS=‘grep ’Total errors in alignments’ $GENSTATFILE | awk ’{print $NF}’‘

export SNPERRORS=‘grep ’Total substitution errors in alignments’ $GENSTATFILE | awk ’{print $NF}’‘

export INDELERRORS=‘grep ’Total indel errors in alignments’ $GENSTATFILE | awk ’{print $NF}’‘

echo -e $REF“ \t”$QUAL“\t”$COVERED“\t”$TOTERRORS“\t”$SNPERRORS“\t”$INDELERRORS

done

To consider only the consensus quality in the regions of HG002 covered by both the GRCh38-constructed and the CHM13-constructed genomes, the following shell commands were run:

for grch38file in ‘ls./GRCh38_GQC/GRCh38_Revio_DV_HiFiPhased.mingq[1234]0/GRCh38_*mingq[1234]0/*.benchcovered

.v1.1.bed‘; do

export QUAL=‘echo $grch38file | sed ’S/.*mingq//’ | sed ’s:\ ..*::’‘

export CHM13FILE=‘echo $grch38file | sed ’s/GRCh38/CHM13/g’‘

export GRCH38ERRORS=‘echo $grch38file | sed ’s/.benchcovered.v1.1.bed/.errortype.v1.1.bed/’‘

export CHM13ERRORS=‘echo $GRCH38ERRORS | sed ’s/GRCh38/CHM13/g’‘

echo $QUAL $grch38file $CHM13FILE $GRCH38ERRORS $CHM13ERRORS

bedtools intersect -a $grch38file -b $CHM13FILE -u > intersected_covered_regions.gq$QUAL.bed

awk ’$(NF-1)==“SNV” {OFS=“\t”; print $1, $2, $3}’ $GRCH38ERRORS > grch38_snv_errors.gq$QUAL.bed

awk ’$(NF-1)==“INDEL” {OFS=“\t”; print $1, $2, $3}’ $GRCH38ERRORS > grch38_indel_errors.gq$QUAL.bed

awk ’$(NF-1)==“SNV” {OFS=“\t”; print $1, $2, $3}’ $CHM13ERRORS > chm13_snv_errors.gq$QUAL.bed

awk ’$(NF-1)==“INDEL” {OFS=“\t”; print $1, $2, $3}’ $CHM13ERRORS > chm13_indel_errors.gq$QUAL.bed

bedtools intersect -a grch38_snv_errors.gq$QUAL.bed -b intersected_covered_regions.gq$QUAL.bed -u > grch38_snv_err ors.gq$QUAL.intersected.bed

bedtools intersect -a grch38_indel_errors.gq$QUAL.bed -b intersected_covered_regions.gq$QUAL.bed -u > grch38_indel_erro rs.gq$QUAL.intersected.bed

bedtools intersect -a chm13_snv_errors.gq$QUAL.bed -b intersected_covered_regions.gq$QUAL.bed -u > chm13_snv_erro rs.gq$QUAL.intersected.bed

bedtools intersect -a chm13_indel_errors.gq$QUAL.bed -b intersected_covered_regions.gq$QUAL.bed -u > chm13_indel_err ors.gq$QUAL.intersected.bed done

for qual in ‘echo “10 20 30 40”‘; do

export INTERSECTEDCOV=‘awk -F “ \t” ’{sum += $3–$2} END {print sum}’ intersected_covered_regions.gq$qual.bed‘;

export GRCH38ERRORS=‘cat grch38_indel_errors.gq$qual.intersected.bed grch38_snv_errors.gq$qual.intersected.bed | wc -l′ ;

export CHM13ERRORS=‘cat chm13_indel_errors.gq$qual.intersected.bed chm13_snv_errors.gq$qual.intersected.bed | wc -l′ ;

echo -e $qual “ \t”$INTERSECTEDCOV“\t”$GRCH38ERRORS“\t”$CHM13ERRORS

done

##### Comparison of genome and variant benchmarking errors

###### Variant benchmark generation and comparison using hap.py.

Genome benchmarking and variant benchmarking are theoretically based on the same information, but it is not obvious how they can be compared due to their different representations of accuracy. To explore this, we generated variants using the curated HPRC assembly ^40^ (Table S12) and compared them to a variant benchmark using hap.py. We then compared these variant benchmarking results to accuracy reports for the same assembly evaluated against the T2T-HG002v1.1 genome benchmark.

We generated the genome benchmarking results against HG002v1.1 by running an early version of GQC called “q100bench” on alignments of the curated HPRC assembly to the v1.1 benchmark. The results of this analysis are similar to the results described in the section “Evaluation of test assemblies using GQC”.

To perform the variant benchmarking using hap.py, we aligned the HPRC assembly to GRCh38 with minimap2 (v2.28-r1209) and custom parameters -z200000,10000,200 and called variants using dipcall. Then we ran hap.py using benchmarking best practices ^12^ to compare the HPRC assembly-based variant calls to a variant benchmark we had generated as part of creating the “HG2-T2TQ100” assembly-based variant benchmark with https://github.com/usnistgov/defrabb available at https://ftp-trace.ncbi.nlm.nih.gov/ReferenceSamples/giab/data/AshkenazimTrio/analysis/NIST_HG002_DraftBenchmark_defrabbV0.019-20241113/).

This hap.py output and the genome benchmarking results described above were used as input for the next two analyses.

##### Overall comparisons of QV and precision/recall

We first asked how the overall performance metrics for genome (QV) and variant benchmarking (F1, precision, recall, variants per base, etc) compared to each other for the curated HPRC assembly. We filtered the hap.py output from comparing the HPRC haplotypes to the HG2-T2TQ100 variant benchmark to a subset of stratifications in complex repeats and hard-to-map regions. We then used the same stratifications to subset the genome benchmarking errors after having projected them onto GRCh38, including only those that landed in the small variant benchmarking regions. Errors of length 1 were deemed “SNVs” and the rest “INDELs” which we then compared likewise to the same categories in the variant benchmark across each stratification.

Within each stratification, metrics were computed as follows. The numbers of false negatives (FNs) and false positives (FPs) were taken from the columns “TRUTH.FN” and “QUERY.FP” in the hap.py output. FN+FP was merely the sum of these two. These counts per base were obtained by normalizing them to Subset.IS_CONF.Size in the hap.py output, which corresponds to the number of base pairs within the indicated stratification. QV (genome error per base) was obtained by also normalizing the number of genome errors that fell within the stratification on GRCh38 to Subset.IS_CONF.Size. Precision, Recall, and F1 were taken as-is from the columns “METRIC.Precision”, “METRIC.Recall”, and “METRIC.F1_Score” in the hap.py output. These three metrics were then inverted (1 – metric) to make them directionally similar to QV (i.e., after Phred-scaling, higher corresponds to fewer errors). Each metric was then Phred-scaled and plotted within each stratification and for SNVs and INDELs. These results were plotted in Figures 5 and S9.

###### Base-level comparison between genome errors and variants.

We next asked how variants and genome errors compared to each other at an individual level; for instance, we observed that a single genome benchmarking error often corresponded to multiple variant benchmarking errors, and it is not immediately obvious why this happens. For this analysis, we only considered small variants and not structural variants with respect to GRCh38 (>50bp). In addition, since hap.py does not phase its output, we added phasing back into the hap.py output by using ‘bcftools merge’ to merge it with the phased VCF file created by dipcall. After merging, we directly compared simple variants which had an unambiguous representation, or replayed clusters of variants within ∼10bp of each other in order to determine correct phasing. This recovered the phasing of 99.998% (4558745/4558833) of variants.

These variants (in vcf format) were then “unzipped” into two bed-like files corresponding to variants against GRCh38 for each haplotype; specifically we generated files which contained, for each variant position, the REF allele and the ALT allele for both the T2T-HG002v1.1 and the HPRC haplotype (either mat or pat). Hereafter these split variants are referred to as “variant alleles” to distinguish from variants in VCF format which often represent a variant relative to two haplotypes. This format enabled us to easily check if the two assemblies had a variant relative to each other (and thus if a genome error should be expected at that position) and if either had a variant relative to GRCh38. Each variant allele was then padded with 50bp on either side.

Next, we needed to project genome errors from the T2T-HG002v1.1 assembly coordinate system to the GRCh38 coordinate system. We first generated.paf files by aligning each of the v1.1 assembly haplotypes to GRCh38 using minimap2 (with ‘-c –paf-no-hit –cs -z200000,10000 -xasm5’). ^113^ We then projected the genome errors using the projection script “project_blocks_multi_thread.py” from the flagger tool. ^118^ Note that each genome error bed file from GQC (each file corresponded to one of the two HPRC assembly haplotypes) contained coordinates for its evaluated HG002v1.1 haplotype (paternal or maternal); therefore, each error bed file was split into either haplotype, resulting in four bed files that were projected (two for “like-haplotype” alignments, and two for “cross-haplotype” alignments where the HPRC assembly mapped to the opposite HG002v1.1 haplotype). These “cross-alignments” were relatively few and analysed separately. We then subset the remaining projected variants to the small variant regions, which excludes structural variants (those >50bp) (https://giab-data.s3.amazonaws.com/defrabb_runs/20241009_v0.018_HG002Q100v1.1/results/draft_benchmarksets/GRCh38_HG002-T2TQ100v1.1-dipz2k_smvar-excluded/GRCh38_HG2-T2TQ100-V1.1_smvar_dipcall-z2k.benchmark.bed ). We also excluded any genome errors which had a difference of >50bp between the HG002v1.1 and HPRC assemblies.The remaining genome errors were then compared to the variants.

Next, we intersected variant alleles and genome errors (now both in bed format) using ‘intersectBed -a <A> -b <B> -loj’. We performed this intersection both ways (i.e., with “A” and “B” being either variant/genome error or the reverse) for both haplotypes in order to obtain overlaps and non-overlaps in either category. Note that the 50 base pair padding added to the variants meant that this intersection actually found variant alleles/errors “near” each other. For genome errors and variant alleles which intersected, we next attempted to “match” either with the other by first applying the truth and query variants to GRCh38, and then substituting the projected sequence from the T2T-HG002v1.1 and HPRC assemblies to their mapped coordinates in GRCh38 (essentially treating each genome error as a “variant” relative to GRCh38). A “match” was obtained if the resulting sequence from both truth/T2T-HG002v1.1 and query/HPRC were equal. Note that a “match” may require multiple variants and/or genome errors to be considered at once. In practice, ∼43% of variant alleles/errors intersected one-to-one; matches were trivial to determine in these cases. For more complex cases, we used a combinatorial optimization algorithm to replay different combinations of errors and variant alleles. ∼11% of these variant alleles contained multiple error/variant allele combinations which matched one-to-one that happened to be near each other. The results of this matching process were summarized in Figures S10A, S10B, and S11A.

Lastly, we intersected these match results (per haplotype) with the original hap.py vcf file which contained the benchmarking results. This allowed us to determine the number/type of genome errors that matched with the number/type of variants, as well as whether or not a true error (i.e., mismatch between T2T-HG002v1.1 and HPRC) actually resulted in a FP or FN label from hap.py. In total, 41545 variants encoded for a genome error, 27393 had a perfect match, 6761 intersected with a genome error but failed the match algorithm (potential match) and the remainder did not have a hit (due to failed liftover, complex variant representation, potential bugs in hap.py, errors in variant benchmark, or missing genome errors in GQC). In 17628/27393 (64.4%) of cases, one variant matched with one match on one or both haplotypes, which was the basis for the “1 Line” categories in Figures S10D and S10E. In the remaining cases, a group of matched variants alleles/errors was spread across multiple lines in the hap.py vcf file. 7616/27393 (27.8%) of these were relatively simple variants that hap.py represented as two lines for some reason (for example, a “0|1” and “.” for HG002v1.1 and HPRC respectively on one line followed by a “1|0” and “1|1” on the next line was likely a collapse that could have been written as “1|2” and “1|1”). These formed the basis for the “2 Line” categories in Figures S10D and S10E. All other cases (2149/27393) were deemed “complex” (example in Figure S12; Table S19). Assigning each variant/variant group with its matches was performed according to the definitions in Table S16 on the basis of their GT fields.

###### Equivalencies between variant benchmarking errors and genome benchmarking errors.

In general, we expect that genome and variant benchmarking should be roughly equivalent for non-structural variants that lie within the confident regions of the GIAB benchmark, with the caveat that finding the correspondence between the two types of errors requires first projecting the genome errors onto GRCh38 coordinates which may be an imperfect operation. Within the variant benchmark regions, we found a few interesting relationships between genome and variant performance metrics (Figures S10D, S10E, S11C, and S11D): 1) what GQC calls “phasing” errors often appear as genotype errors (“collapse errors” in the assembly) where one such error corresponds to either one FN variant (collapse to reference) or one FN and one FP (collapse to non-reference), 2) a minority of phasing errors appear as phase switch errors, which are typically counted as true positive variants in hap.py but could be separately counted as errors by phasing benchmarking tools, 3) ∼8% of genome errors fall in complex variants, primarily in tandem repeats and homopolymers (Figure S10C), so that a single genome benchmarking error can cause at least two FP or FN variant calls, (example in Figure S13). 4) Consensus errors most often corresponded to at least one FP variant.

##### Categories of variants and their relation to the genome errors provided by GQC

###### Collapses.

One common type of variant-based error is known as a “collapse” where the true genotype and the test genotype are heterozygous and homozygous, respectively, and one allele is shared between the two. Usually this results when a given region has a difference between the two haplotypes but lacks enough reads to support this difference, resulting in the true sequences being “collapsed” into one sequence in the test genome. The collapsed sequence may match one of the true sequences or neither. In the latter case, we found these were almost always the result of the truth having two insertions/deletions (indels) of differing lengths and the collapsed query sequencing having an intermediate length (Figure S11B); we thus called these “average collapses” below.

14538/30505 genome errors (47.7%) matched with collapses, and 70.8% of these were phasing genome errors (Figure S11C). 75.0% of these were “exact collapses” where one allele matched, and therefore each of these variants matched with exactly one genome error (Figure S11D). Within this subcategory, 95.2% were spread on one line with either one FN or one FN and FP, and the remainder were on two lines with one FN and FP. For those with one line in the VCF file, variants with only FN are cases where the collapse is to the reference allele (i.e., truth is 1|0, query is 0|0) and variants with FN/FP are those that collapse to alternate (1|0 to 1|1). Curation showed that collapses spread across two lines were likely cases involving a single-nucleotide polymorphism (SNP) and an indel, which hap.py will split into two lines to account for the two different variant types. 25.0% of genome errors which matched with a collapse variant matched in pairs (one for each haplotype), and these corresponded to “average collapses” as described above.

This analysis shows how variants and genome errors can be equated differently depending on the reference, which may lead to different benchmark performance metrics. For collapses, one genome error will correspond to one FN if the variant collapses to reference, but will also include an FP if the variant collapses to alternate. An average collapse will generally equate to one FP and one FN (89.5% in this case); when this isn’t true the variant is likely part of a larger, more complex cluster of variants that cause the labels to be counted differently.

###### Sequencing errors.

This variant category encompassed a variety of genotypes (Table S16) all of which were likely caused by an error in the underlying sequencing platforms. 100% of these (11962 variants) occurred in either a homopolymer or a tandem repeat, and 97.2% of these were INDELs (Figure S10C). These likely arose due to differences in length between the truth and query sequences. For instance, reverse collapses (so named because they have the opposite genotype manifestation relative to truth and query) were likely in a homopolymer that had a homozygous length in the truth but was either too long or too short on one haplotype in the query.

###### Misphases.

A small number of variants (648) matched perfectly except their phasing was flipped which we called “Misphases”. hap.py marked these as true positives due to the fact that vcfeval (the comparison engine within hap.py) ignores phasing when comparing variants. Unsurprisingly, 87.2% of the corresponding genome errors were phasing errors in GQC, and all included two genome errors since both haplotypes mismatched the truth. This is the simplest instance where vcfeval will undercount true errors due to it not taking phasing into account. There are other instances where vcfeval scored a variant as TP even though the phasing did not match; in our data these were relatively complex cases and thus appeared under the “complex” heading (Figures S10D and S10E).

We also identified variants that were likely due to both a phasing flip and a sequencing error, which we called “Misphase/seq errors.” (ie 0|2 vs 2|1, so one allele mismatches, and the matching allele has the wrong phase). These were the only variant category for which the genome error type was “MIXED” (i.e., there were two genome errors on each haplotype, one of which was a consensus error and one of which was a phasing error) (Figures S10D and S10E).

### QUANTIFICATION AND STATISTICAL ANALYSIS

Figures 2A, 3, and 4 were created using R scripts available in the manuscript’s software archive. ^114^ For plotted sequence accuracy or error rates, Wilson binomial confidence intervals were calculated and displayed whenever they were larger than the size of the plotted points (e.g., in Figures 3D and 4F). Figure 5 was created by quantifying select stratifications in the hap.py output comparing the HPRC haplotypes to the HG2-T2TQ100 benchmark as described in more detail in STAR Methods. GQC’s implementation of a hidden Markov model for phasing scaffolds is described in this manuscript’s STAR Methods.

## Supplementary Material

1

2

3

4

5

6

7

8

9

10

11

12

13

14

15

16

17

18

19

20

SUPPLEMENTAL INFORMATION

Supplemental information can be found online at https://doi.org/10.1016/j.cell.2026.06.016.

## Figures and Tables

**Table 1.** Number of genes annotated in T2T-HG002v1.1 and T2T-CHM13v2.0

Gene type	HG002 MAT 1–22	HG002 PAT 1–22	HG002 MAT ChrX	HG002 PAT ChrY	CHM13 All Chrs
Protein coding	19,117	19,086	839	104	20,008
Non-coding (incl. lncRNA)	21,809	21,730	560	175	22,474
Pseudogene	16,072	16,031	926	415	17,281
Total	56,998	56,847	2,325	694	59,763

Gene biotypes were identified using the attribute “gene_biotype” from the T2T-CHM13 reference annotation. The T2T-CHM13 counts include all autosomes as well as both ChrX and ChrY.

**Table 2:** 

Platform	Het (%)	Kcov (het. peak)	Error rate (%)	Model fit	Genome size est.	Bps with coverage < 2x
Element Aviti UltraQ	0.25%	34.0	0.06%	0.82	2,908,208,748	2,180,013
Illumina HiSeq 2x250,	0.27%	30.7	0.26%	1.06	2,903,028,543	1,012,551
PacBio Onso	0.29%	22.5	0.03%	0.74	2,904,805,881	12,216,638
PacBio Revio with SPRQ	0.28%	50.6	0.08%	1.08	2,866,227,392	
PacBio Revio HiFi	0.23%	34.0	0.09%	0.42	2,923,638,455	

**Table:** KEY RESOURCES TABLE

REAGENT or RESOURCE	SOURCE	IDENTIFIER
Biological samples		
NIST Reference Material DNA, HG002	National Institute of Standards and Technology (NIST)	NIST RM 8391
Personal Genome Project DNA, Ashkenazi male huAA53E0	Coriell	NA24385
Deposited data		
T2T-HG002v1.1 consensus sequences	Genbank, this paper	GenBank: GCA_018852605.3, GCA_018852615.3
HiFi Sequel II data	Pacific Biosystems	SRA: SRR17284855, SRR17284856, SRR17284854, SRR17284853, SRR17284857, SRR17284858, SRR18244890, SRR18244889, SRR18246375, SRR18246374, SRR18360739, SRR13684284, SRR13684284, SRR18239007, SRR18239006, SRR18239005, SRR18239004, SRR18358816
HiFi Revio data	Pacific Biosystems	https://downloads.pacbcloud.com/public/revio/2022Q4/,https://s3-us-west-2.amazonaws.com/human-pangenomics/index.html?prefix=T2T/HG002/assemblies/polishing/HG002/v1.0/mapping/hifi_revio_pbmay24/
ONT data	HPRC Liao et al. (2023)^10^	SRA: SRR18363760, SRR18363759, SRR18363748, SRR18363744, SRR18363743, SRR18363742, SRR18363741, SRR18363740, SRR18363739, SRR18363738, SRR18363758, SRR18363757, SRR18363756, SRR18363755, SRR18363754, SRR18363753, SRR18363752, SRR18363751, SRR18363750, SRR18363749, SRR18363747, SRR18363746, SRR18363745, SRR24660797, SRR24660796
Illumina data	Zook et al.^42^	SRA: SRX847862-SRX848005
Element avidity PCR free data	Arslan et al.^107^	SRA: SRX17079410
Element cloud break data	Element Biosciences	https://s3-us-west-2.amazonaws.com/human-pangenomics/index.html?prefix=T2T/scratch/HG002/sequencing/element/trio/
Onso sequencing by binding data	Mark Fleharty, Broad Institute	https://s3-us-west-2.amazonaws.com/human-pangenomics/index.html?prefix=T2T/scratch/HG002/sequencing/onso/Broad-Onso-HG002/
Strand-seq data	Zook et al.^2^	https://s3-us-west-2.amazonaws.com/human-pangenomics/index.html?prefix=working/HPRC_PLUS/HG002/raw_data/Strand_seq/2019-12-16-HWVTJAFXY/
Ash1v2.0 haploid HG002 assembly	Shumate et al.^53^	https://github.com/AshkenaziGenome/Assembly
2021 hifiasm HG002 assembly	Cheng et al.^54^	https://zenodo.org/record/4393631
Release 1 HPRC HG002 assembly	Liao et al.^10^	s3://human-pangenomics/working/HPRC_PLUS/HG002/assemblies/year1_f1_assembly_v2_genbank
Verkko v1 trio-based HG002 assembly	Rautiainen et al.^28^	https://zenodo.org/records/7400747/files/hg002_verkko_hifi_ont_trio.fasta.gz
London Calling 2024 HG002 assembly	Epi2Me	https://epi2me.nanoporetech.com/lc2024_t2t/
Experimental models: Cell lines		
HG002 Ashkenazi trio son B-lymphocyte LCL	Coriell; NIST; PGP	GM24385; RM8391/RM8392; RRID: CVCS_1C78
HG003 Ashkenazi trio father B-lymphocyte LCL	Coriell; NIST; PGP	GM24149; RM8392; RRID: CVCL_1C54
HG004 Ashkenazi trio mother B-lymphocyte LCL	Coriell; NIST; PGP	GM24143; RM8392; RRID: CVCL_1C48
Software and algorithms		
Verkko v1.0-v1.4.1	Rautiainen et al.^28^	https://github.com/marbl/verkko
Verkko2 v2.0-v2.2.1	Antipov et al.^77^	https://github.com/marbl/verkko
Merqury v1.3	Rhie et al.^52^	https://github.com/marbl/merqury
Flye assembler v2.7-b1585	Kolmogorov et al.^108^	https://github.com/mikolmogorov/Flye
GraphAligner v1.0.16	Rautiainen and Marschall^109^	https://github.com/maickrau/GraphAligner
pstools	Garg^110^	https://github.com/shilpagarg/pstools
BWA-MEM v0.7.17-r1188	Li and Durbin^111^	https://github.com/lh3/BWA
Winnowmap2 v2.03	Jain et al.^112^	https://github.com/marbl/winnowmap
minimap2 v2.26, v2.28-r1209	Li^113^	https://github.com/lh3/minimap2
T2T-Polish v1.0	Mc Cartney et al.^59^	https://github.com/arangrhie/T2T-Polish
Archive of code used for this manuscript	Hansen^114,115^	https://doi.org/10.5281/ZENODO.17128497
Release v0.1 software for managing github issues	Hansen^114,115^	https://doi.org/10.5281/ZENODO.15491811
PGAS v14	Ebert et al.^87^	https://github.com/ptrebert/project-diploid-assembly
StrandPhaseR version #8b93668	Porubsky et al.^116^	https://github.com/daewoooo/StrandPhaseR
WhatsHap v1.0, v1.1	Martin et al.^117^	https://whatshap.readthedocs.io
Flagger v0.2	Asri et al.^118^	https://github.com/mobinasri/flagger
ModDotPlot v0.9.8	Sweeten et al.^119^	https://github.com/marbl/ModDotPlot
DeepVariant v1.5	Poplin et al.^57^	https://github.com/google/deepvariant
samtools v1.16.1-v1.21, bcftools 1.10.2–140-gc40d090	Danecek et al.^89^	https://www.htslib.org/
NucFreq v0.1	Vollger et al.^56^	https://github.com/mrvollger/NucFreq
hifiasm v0.18.9-r527, v0.19.5	Cheng et al.^54^	https://github.com/chhylp123/hifiasm
LiftOver	Hinrichs et al.^120^	https://genome.ucsc.edu/cgi-bin/hgLiftOver
RepeatMasker v4.1.0, v4.1.7-p1	Tarailo-Graovac and Chen^121^	https://www.repeatmasker.org/
Geneious Prime v2023.1	Dotmatics	https://www.geneious.com/
IGV v2.16.2	Robinson et al.^122^	https://igv.org/
FreeBayes v1.3.7	Garrison and Marth^123^	https://github.com/freebayes/freebayes
DeepPolisher	Mastoras et al.^48^	https://github.com/google/deeppolisher
Sniffles2 v2.0.7	Smolka et al.^58^	https://github.com/fritzsedlazeck/sniffles
Bedtools v2.31.1	Quinlan and Hall^124^	https://github.com/arq5x/bedtools2
sambamba v1.19	Tarasov et al.^125^	https://lomereiter.github.io/sambamba/
BreakpointR	Porubsky et al.^126^	https://github.com/daewoooo/breakpointR
hg002-q100-annotation v0.0.2	Ji^127^	https://doi.org/10.5281/ZENODO.16880403
Liftoff	Shumate and Salzberg^62^	https://github.com/agshumate/Liftoff
Comparative Annotation Toolkit (CAT)	Fiddes et al.^128^	https://github.com/ComparativeGenomicsToolkit/Comparative-Annotation-Toolkit
gffread	Pertea and Pertea^129^	https://github.com/gpertea/gffread
alphaAnnotation: HumAS-HMMER (modified)	Lukas^130^	https://doi.org/10.5281/zenodo.5715444
Tandem repeats finder v4.1.0	Benson^131^	https://tandem.bu.edu/trf/trf.html
WindowMasker v2.2.22	Morgulis et al.^132^	https://github.com/goeckslab/WindowMasker
SEDEF v1.1	Numanagic et al.^133^	https://github.com/vpc-ccg/sedef
BLASTn v.2.13.0+	Altschul et al.^134^	https://blast.ncbi.nlm.nih.gov/Blast.cgi
ribotin commit d8a73739d5f7a5de3d27904a18e53d5bbfdf14e9	Rautiainen^47^	https://github.com/maickrau/ribotin
Refine_CytoBand_Liftover	Altemose^135^	https://doi.org/10.5281/ZENODO.17089359
nf-LO downloaded from https://get.nextflow.io Feb 23, 2023	Talenti and Prendergast^136^	https://nf-lo.readthedocs.io
Rustybam v0.1.34	Vollger and Hulselmans^137^	https://doi.org/10.5281/ZENODO.15580393 https://doi.org/10.5281/ZENODO.15580393
chaintools v0.1	Chen and Hansen^138^	https://github.com/milkschen/chaintools_bio
SVbyEye	Porubsky et al.^139^	https://github.com/daewoooo/SVbyEye
mosdepth v0.3.3	Pedersen and Quinlan^140^	https://github.com/brentp/mosdepth
HapCUT2 v1.3.4	Edge et al.^91^	https://github.com/vibansal/HapCUT2
happy	Krusche et al.^12^	http://github.com/illumina/hap.py

## Highlights

- A telomere-to-telomere diploid assembly of HG002 achieves near-perfect accuracy
- Personalized diploid genome annotation reveals haplotype-specific gene variation
- Companion software evaluates sequence, assembly, and variant accuracy genome-wide
- New benchmark facilitates transition to genome inference and personalized genomics
