# Supplementary material for: A complete diploid human genome benchmark for personalized genomics
Source: Cell. Author manuscript; Available in PMC 2026 Aug 10. (PMC13456413; doi:10.1016/j.cell.2026.06.016)
Supplement: Supplementary file 1 [file NIHMS2189544-supplement-20.pdf]

Supplementary Figures

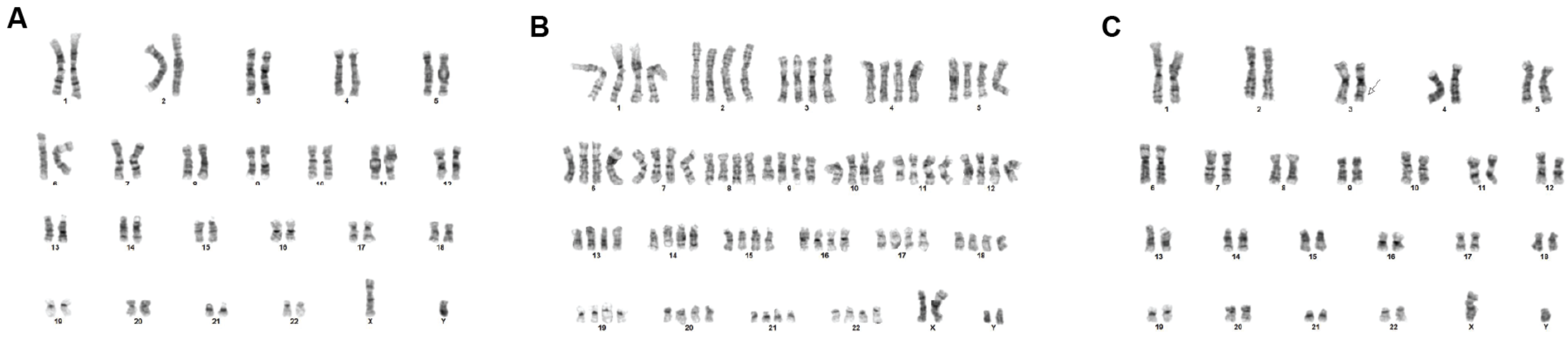

Figure S1: Cytogenetic analysis of the GM24385/HG002 cell line, related to Figure 2. (A) 53 of 73 metaphase cells (73%) showed a normal male karyotype (46,XY). **(B)** 13 cells (18%) showed a tetraploid karyotype (92,XXYY). **(C)** 3 karyotyped cells (4%) showed an inversion on chromosome 3 (46,XY,?inv(3)(q26.3q29), shown with an arrow).

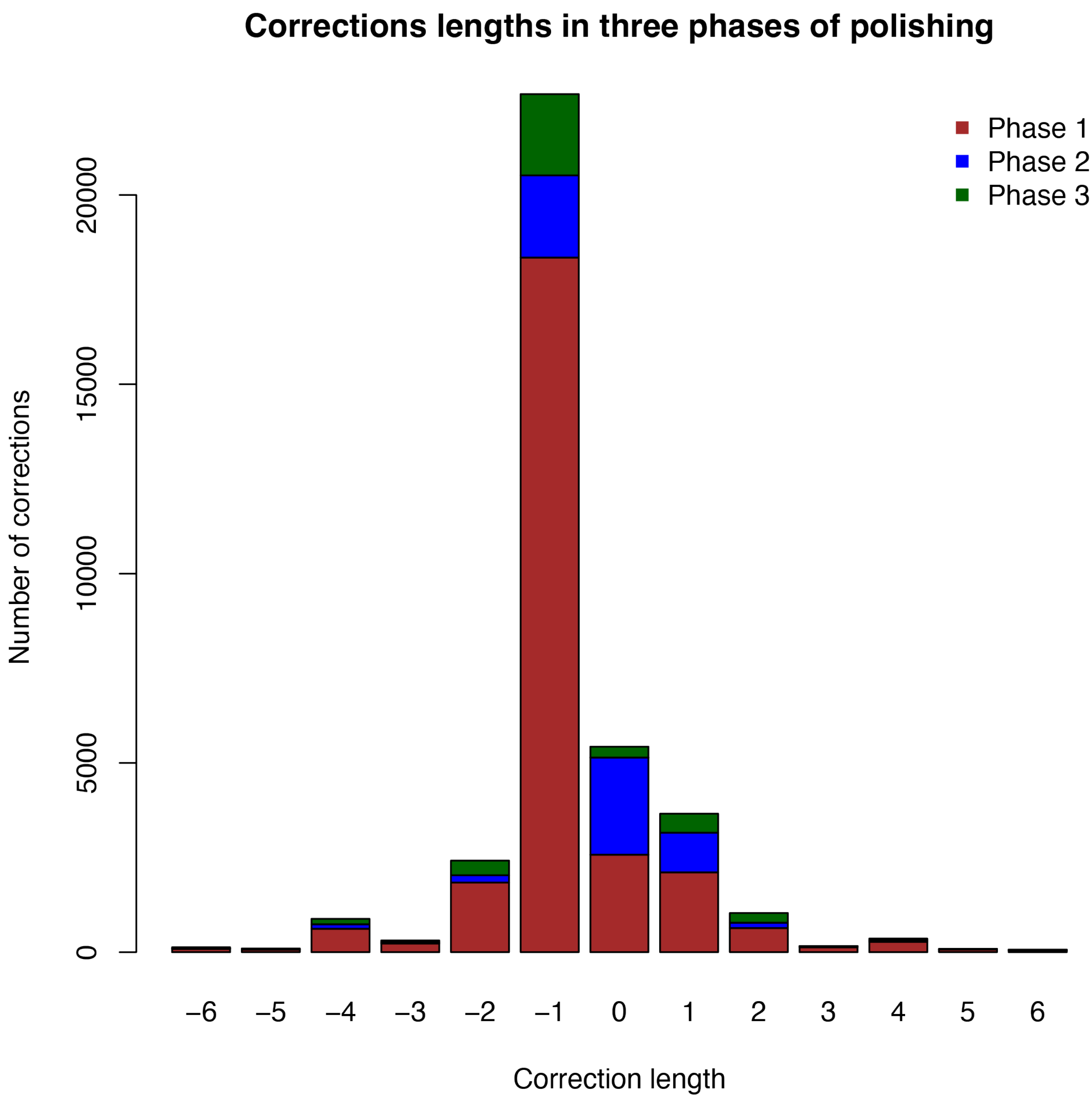

Figure S2: Lengths of small corrections made in three rounds of polishing, related to Figure 2. Histogram of correction lengths in polishing HG002v0.7 to create HG002v0.9 (Phase 1), in polishing HG002v0.9 to create HG002v1.0.1 (Phase 2), and in polishing HG002v1.0.1 to create HG002v1.1 (Phase 3). The length of a correction is defined as the difference between the new (corrected) allele length and the original (uncorrected) allele length. Roughly the same number of single nucleotide corrections (length 0) were made in Phases 1 and 2 (2574 and 2555, respectively), with a smaller number (291) in Phase 3. The plot shows that the large majority of corrections made overall were deletions of single bases, usually shortening homopolymer sequences that were found to be one base too long.

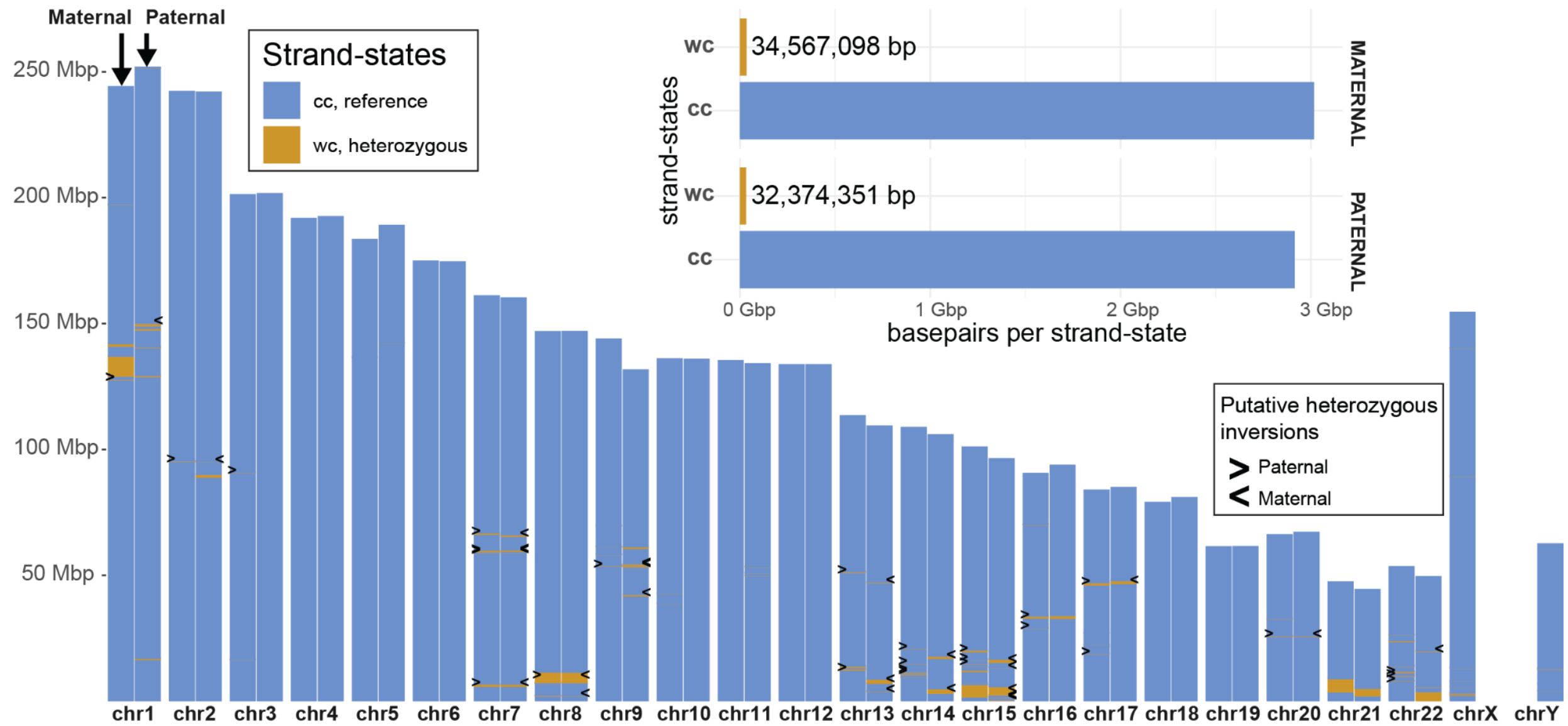

Figure S3: Strand-seq assembly evaluation of HG002v1.1, related to Figure 2. An ideogram shows Strand-seq-based genotyping marking regions genotyped as in complete agreement with the assembly (reference, ‘cc’) and regions genotyped as heterozygous (‘wc’). Heterozygous regions are generally caused by low mappability regions such as short acrocentric arms, centromeres, and repetitive regions. A subset of these mark positions of heterozygous inversions, highlighted by arrowheads. We did not observe any region genotyped as homozygous inverted, suggesting that no misorientation is present in these haplotype assemblies.

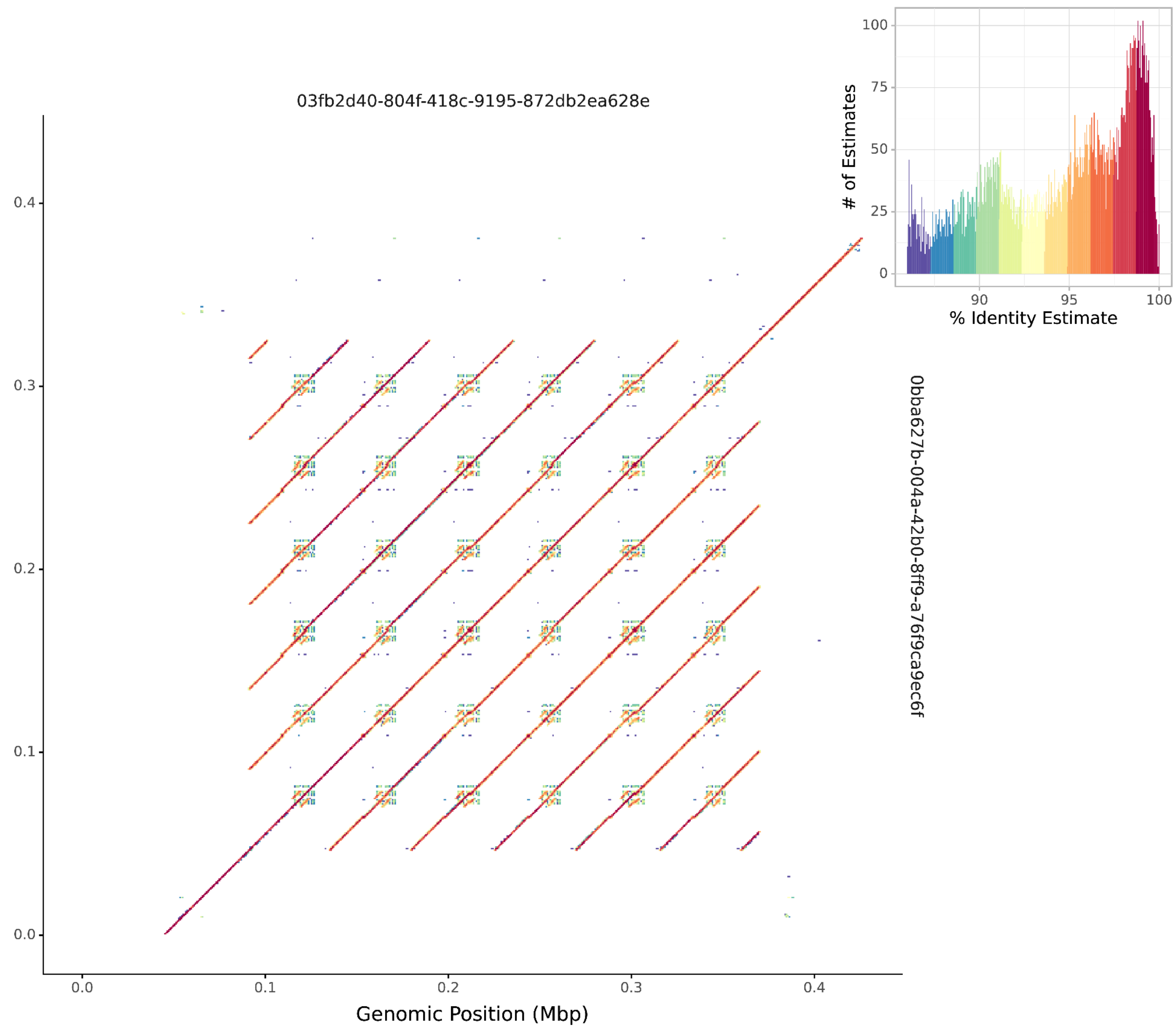

Figure S4: ModDotPlot plot of two ONT ultralong reads which span the paternal chr13 rDNA array, related to Figure 2. Ultralong reads indicate six copies of the rDNA repeat unit, longer than the v0.9 consensus of chr13_PATERNAL's acrocentric p-arm.

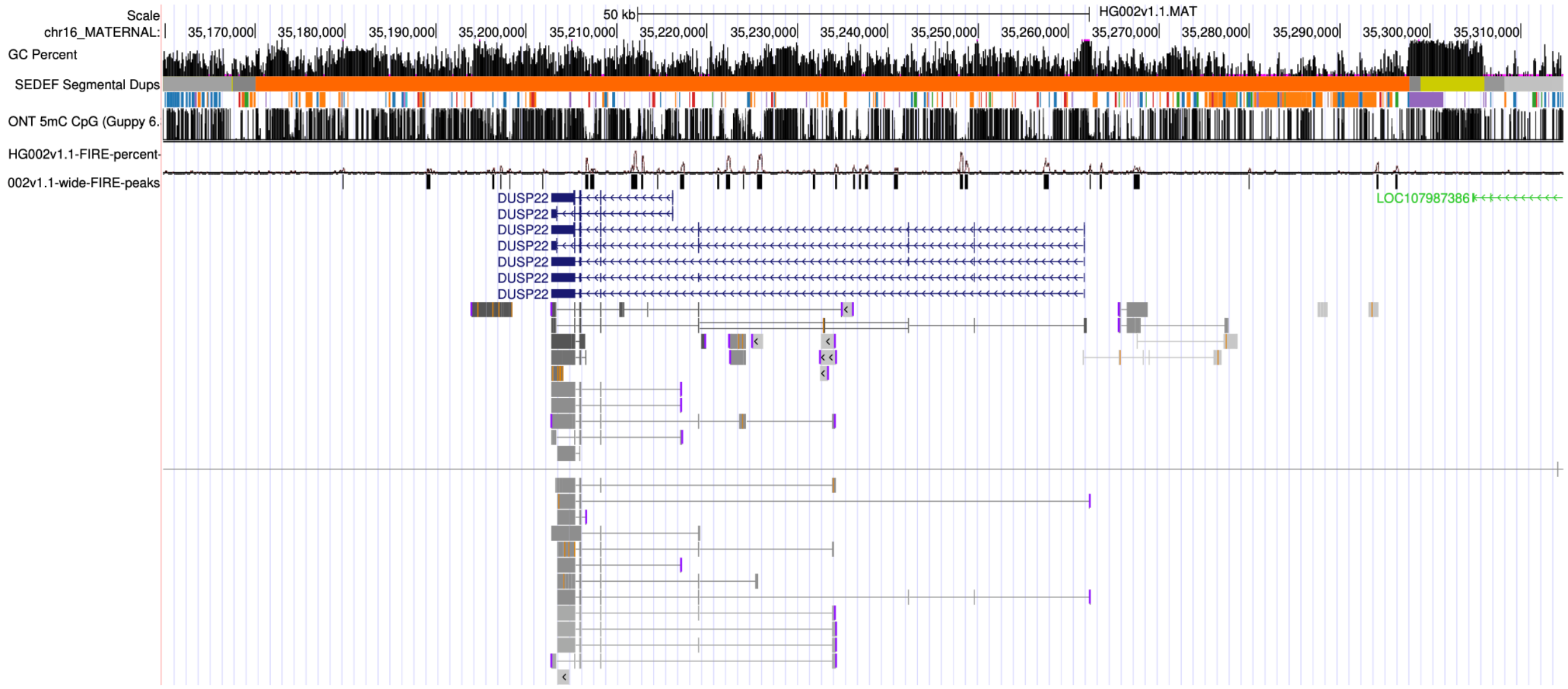

Figure S5: UCSC Browser hub for T2T-HG002v1.1, related to Figure 2. The browser includes diploid gene annotation and functional genomics tracks, as well as sequence-based annotations (GC percent, CpG islands, repetitive elements, segmental duplications, subtelomere repeats, and centromeric satellites) and validation tracks (known issues, depth of coverage, NucFreq output, etc.). Chain file tracks are also included, showing the position of all heterozygous sites and enabling quick locus switching between the two HG002 haplotypes, T2T-CHM13, and GRCh38. Shown here is the maternal haplotype of HG002 Chr16 focused on the *DUSP22B* gene from **Figure 2C**. Browser tracks shown from top to bottom: GC percent, segmentally duplicated regions (orange representing a highly similar duplication), RepeatMasker annotations, ONT-based methylation percentage, Fiber-seq accessibility, Fiber-seq peak calls, gene models, and PacBio Kinnex transcripts. The methylation, Fiber-seq, and transcriptome data jointly support the expression of this additional *DUSP22* copy in the HG002 LCL.

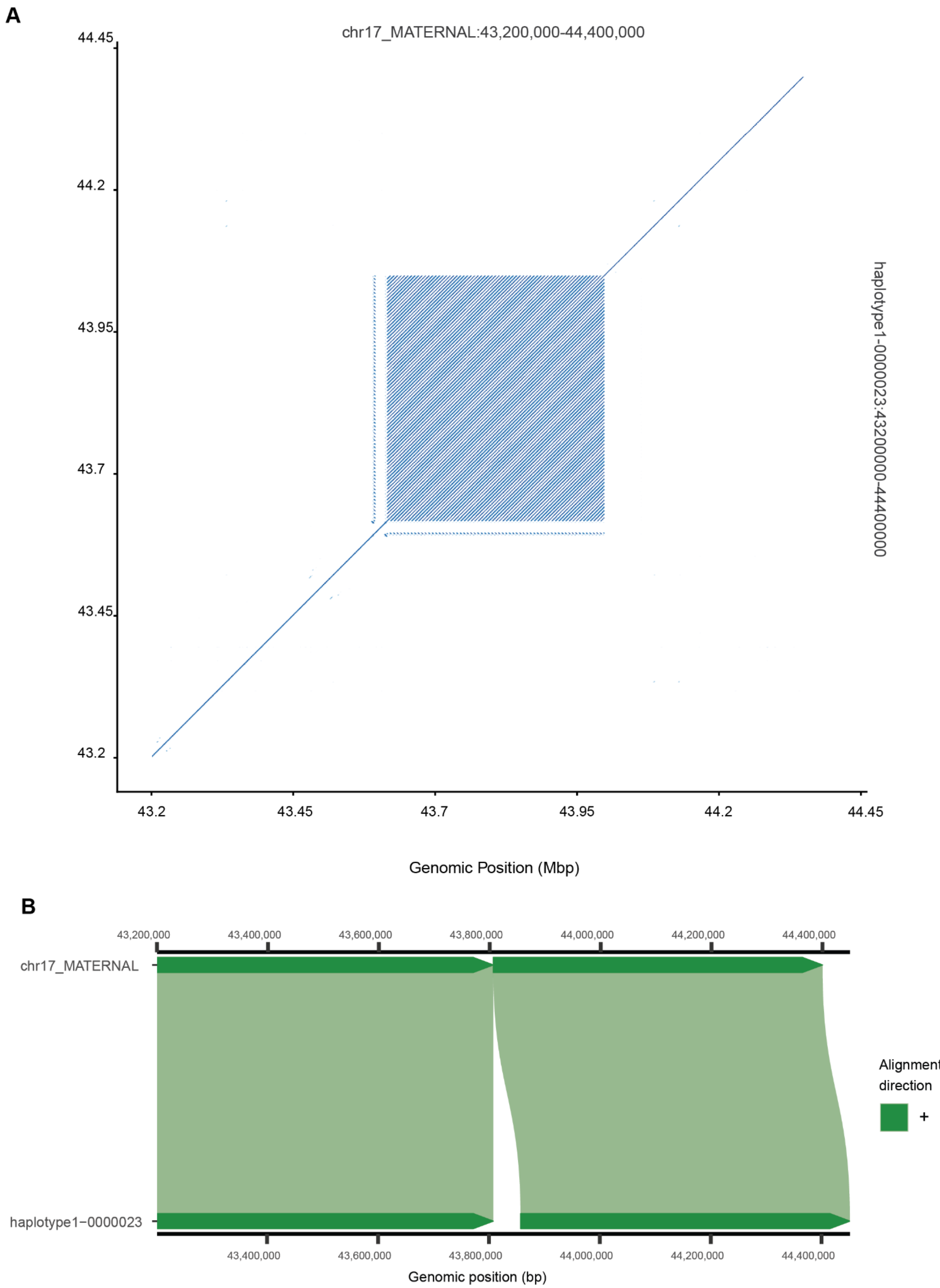

Figure S6: Example of a structural error detected by GQC, related to Figure 3. A 49,414 base pair insertion error detected by GQC in scaffold haplotype1-0000023 of the "ONT LC2024" assembly, as

displayed in (A) a ModDotPlot plot of the region and (B) an SVbyEye plot with T2T-HG002v1.1 in the top row and the LC2024 scaffold in the bottom row.

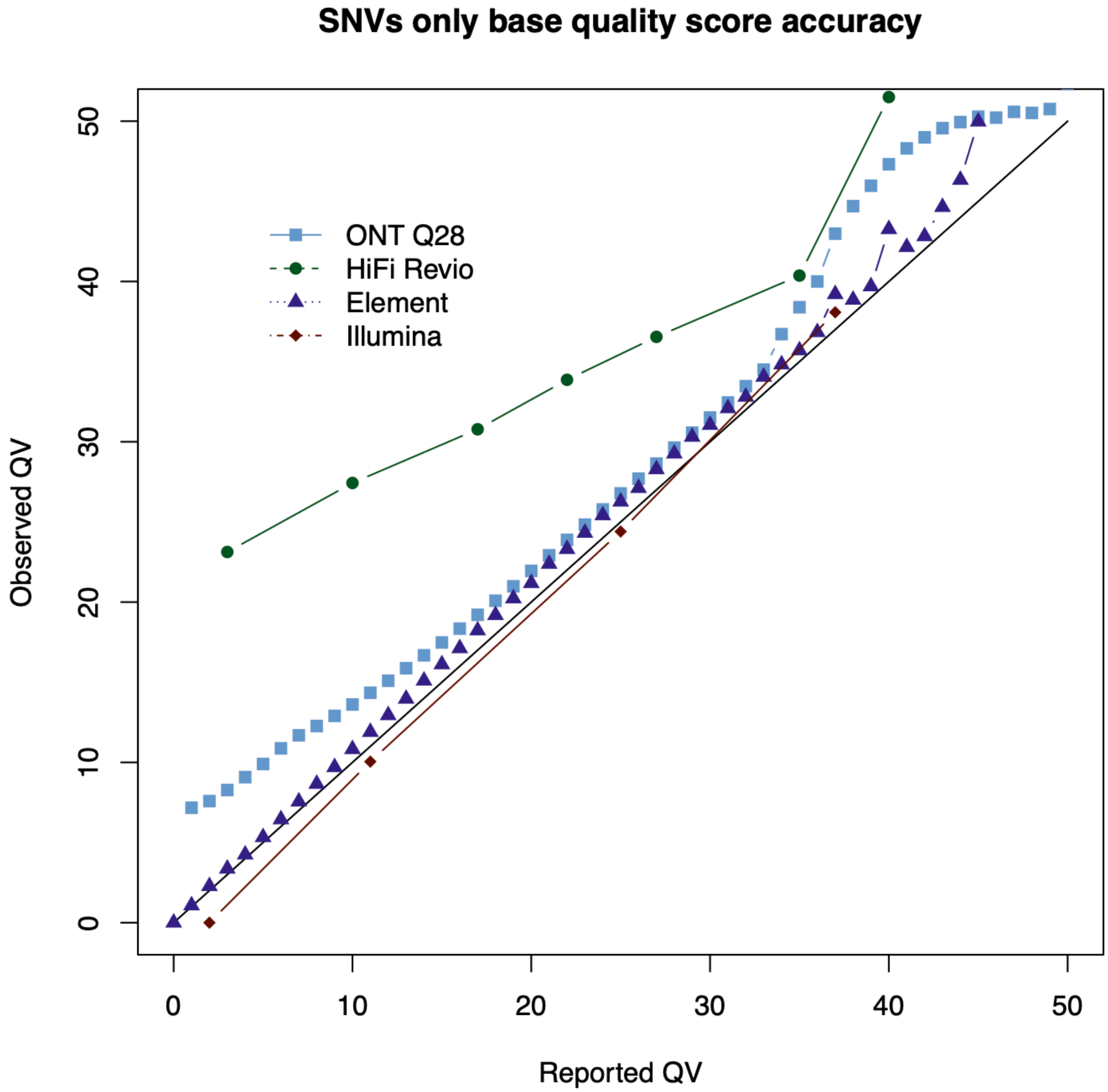

Figure S7: Substitution accuracy for bases with different read base quality scores, related to Figure 4. Substitution accuracy of base quality scores calculated from actual alignment match rates (Observed) versus binned base quality scores reported with the reads (Reported). Unlike the curves in **Figure 4D** of the main manuscript, only substitution errors are used to calculate observed quality in this plot.

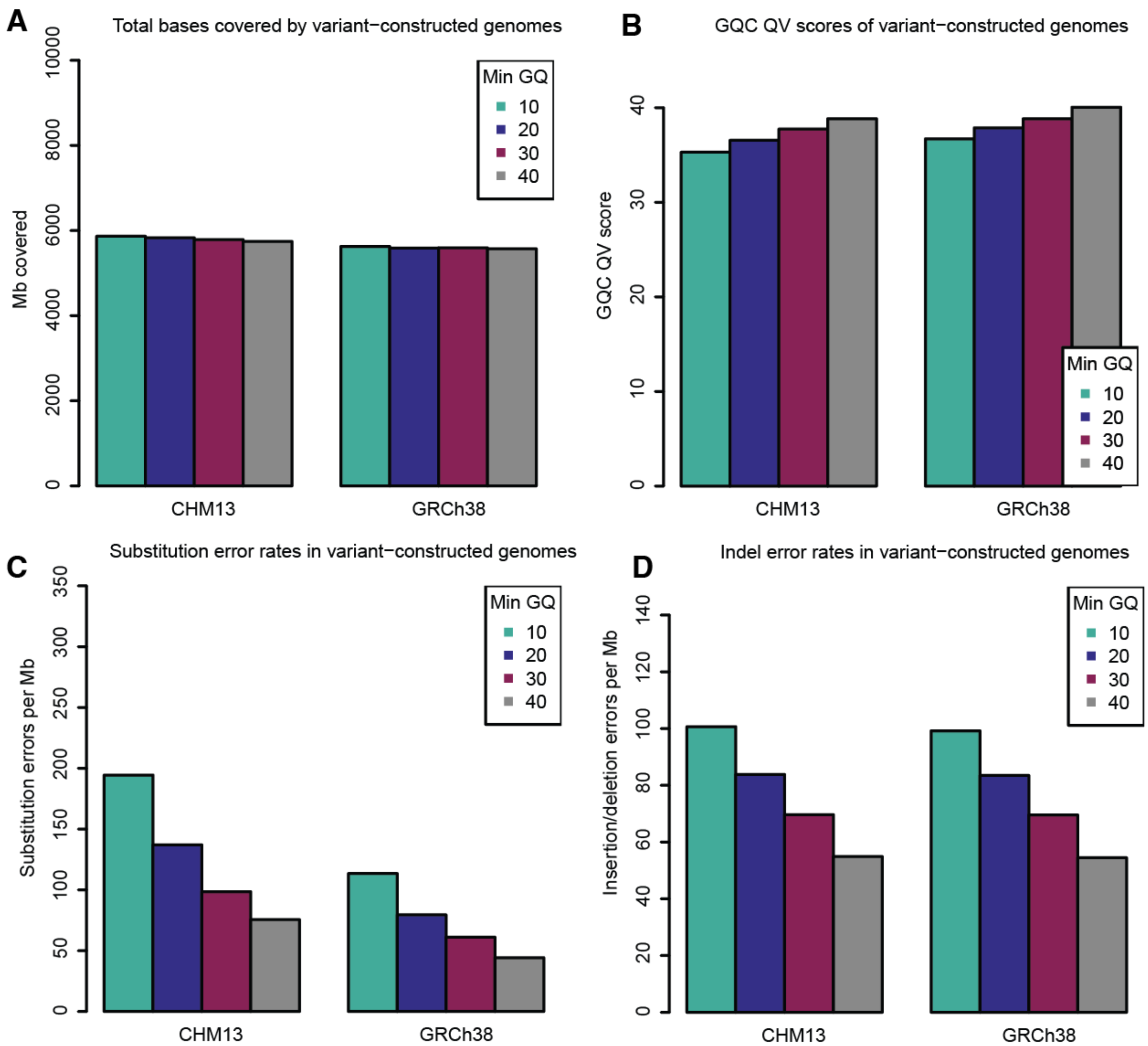

Figure S8: GQC quality statistics for variant-constructed genomes using different minimum genotype quality cutoffs, related to Figure 5. (A) Aligned coverage of v1.1, or total genome benchmark bases covered by aligned sequence in the variant-constructed genome. **(B)** GQC quality scores (phred-scaled) for variant-constructed genomes calculated as -10 times the log, base 10 of the total number of discrepancies divided by the total alignment length. **(C)** Substitution error rate in variant-constructed genomes per megabase of aligned sequence. **(D)** Indel error rate in variant-constructed genomes per megabase of aligned sequence.

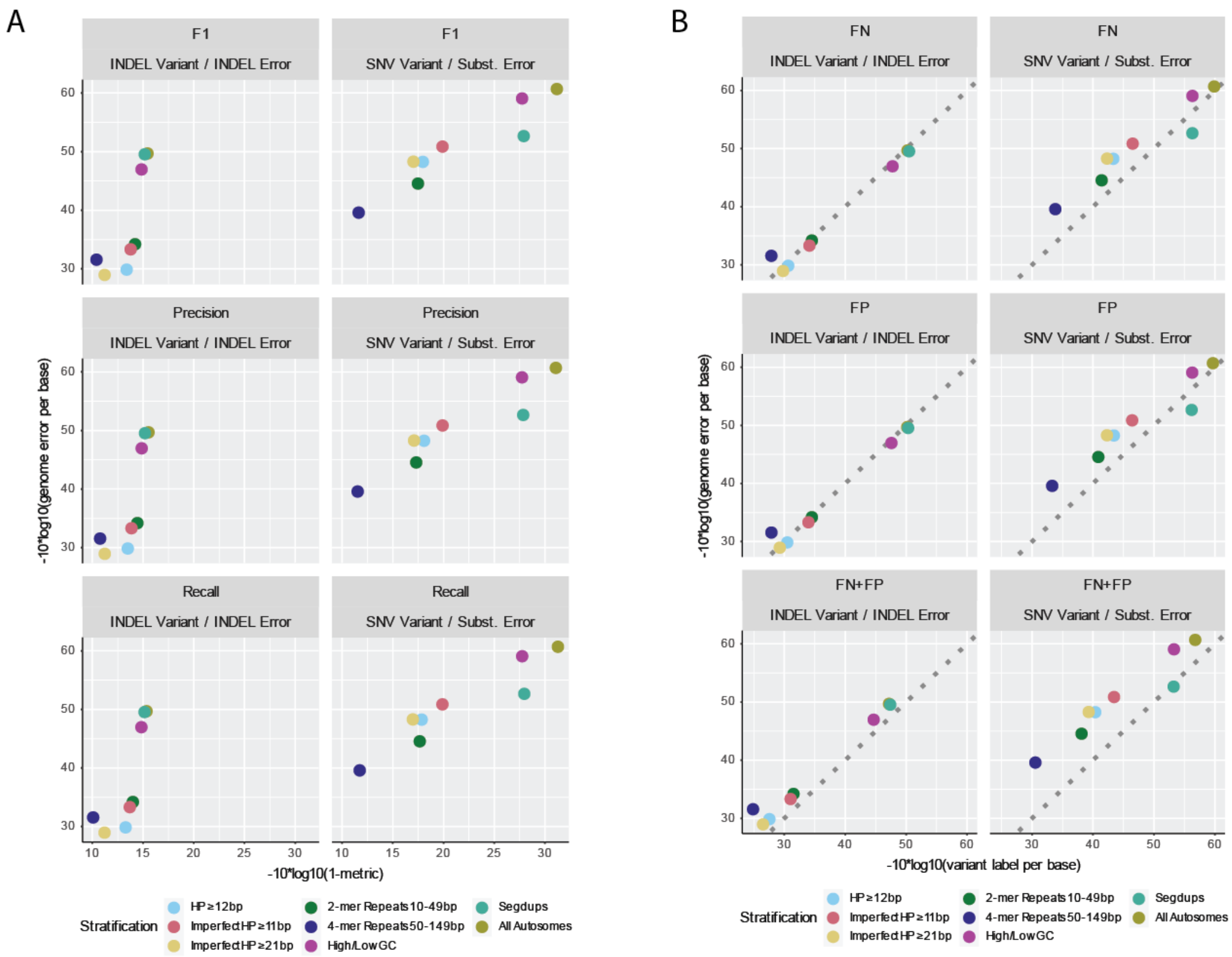

Figure S9: Global performance metric comparison between variant and genome benchmarking across various stratifications, related to Figure 5. (A) Genome errors per base (as reported by GQC) vs F1, precision, or recall reported as a variant benchmarking performance metric (phred-scaled). Genome errors were restricted to those which can be successfully projected onto GRCh38 GIAB small variant benchmark regions. Furthermore, SNV and INDEL designations for genome errors were based on comparison of an HG002 assembly to the benchmark while variant benchmarking SNV and INDEL designations were based on comparison of HG002 to GRCh38. HP = homopolymer. **(B)** Phred-scaled genome benchmarking errors per base vs. phred-scaled false negative and false positive variants per base for SNVs/Substitution Errors or INDELs/INDEL Errors. Points above the dotted line indicate variant benchmark had more errors relative to genome benchmark.

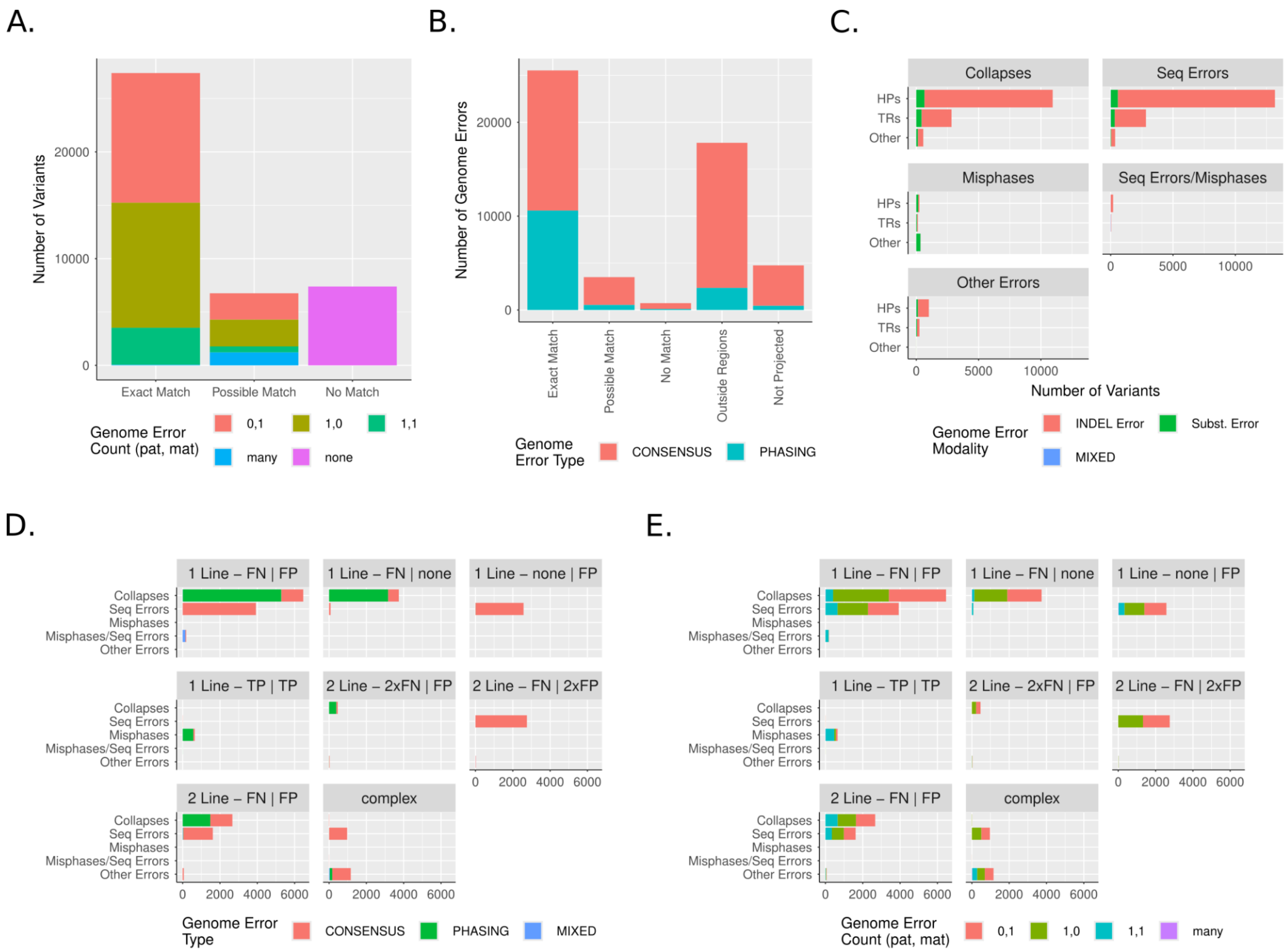

Figure S10: Variants and their corresponding genome benchmarking errors, related to Figure 5. **(A)** Variant-based errors grouped by those that matched with a genome error ("Exact Match"), intersected but failed to match with a genome error ("Possible Match") or intersected with no genome errors ("No Match") colored by how many genome errors were part of the intersection. **(B)** Genome errors grouped by those that matched, intersected, or did not intersect with a variant analogous to **(A)**; in addition, genome errors that projected from T2T-HG002v1.1 to GRCh38 coordinates but landed outside the benchmarking regions ("Outside Regions") or failed to lift over entirely ("Not Projected"). **(C)** Variants grouped by presence in homopolymers (HP), tandem repeats (TRs), or other stratifications. "Substitution Errors" are differences between the T2T-HG002v1.1 and HPRC assemblies for which both alleles are only a single base, and "INDEL Errors" are everything else. (**D-E)** Variants with at least one matching genome error grouped by variant type (y axis) and label/number of lines (header) and either colored by the type of matching genome error(s) **(D)** or number of matching genome errors **(E)**.

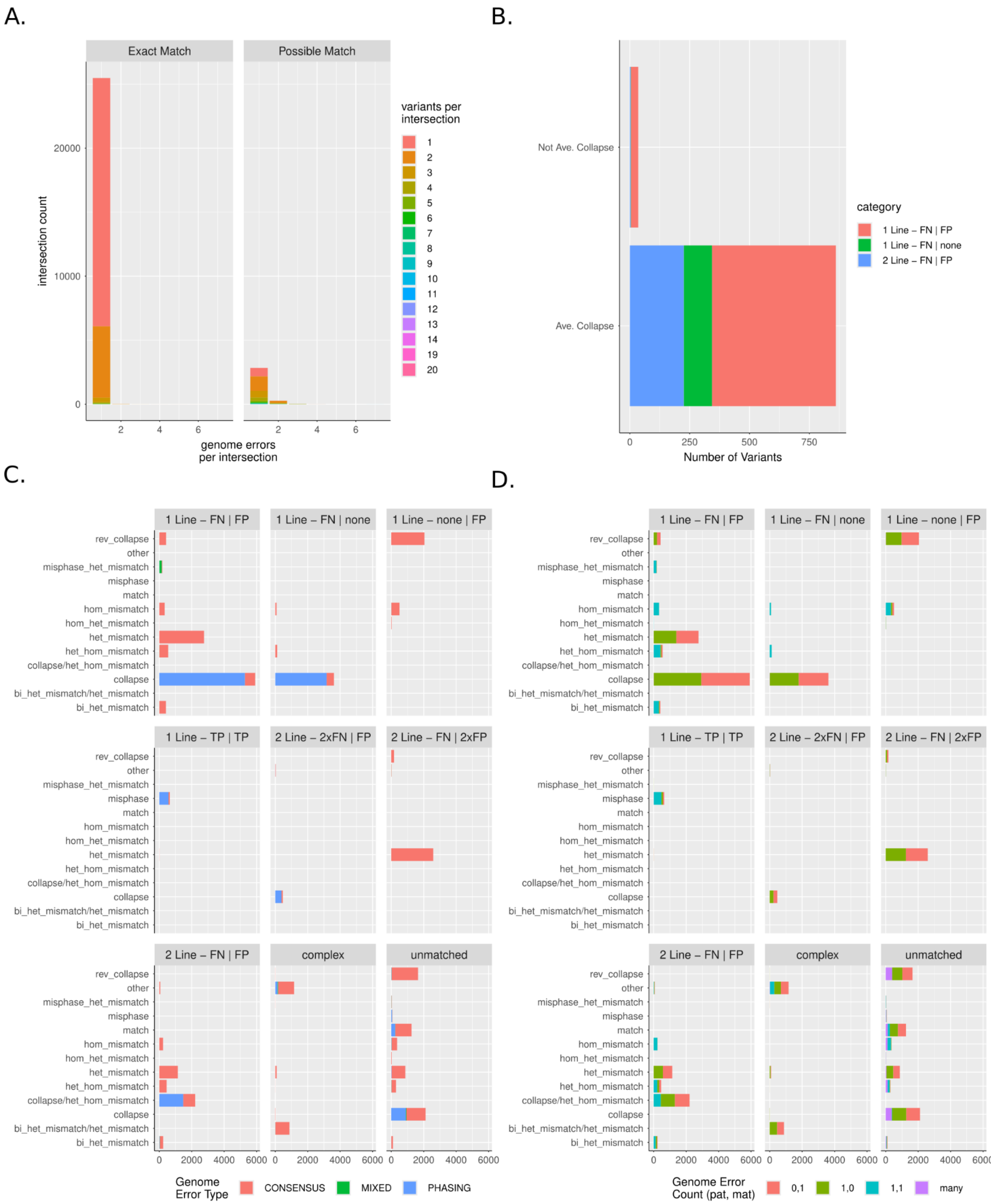

Figure S11: Variants match results and corresponding genome errors with granular categorization, related to Figure 5. (A) Counts of variants and genome errors per intersection that either match (left) or do not match (right); in this case variants are split across both haplotypes so homozygous variants are included twice. **(B)** Counts of variants that are het_hom_mismatches that are

“average collapses” or not **(C-D)** Corresponding categories analogous to **Figure S10C,D** broken down into finer subcategories that correspond directly to variant genotype. See **Table S16** for how these categories are defined.

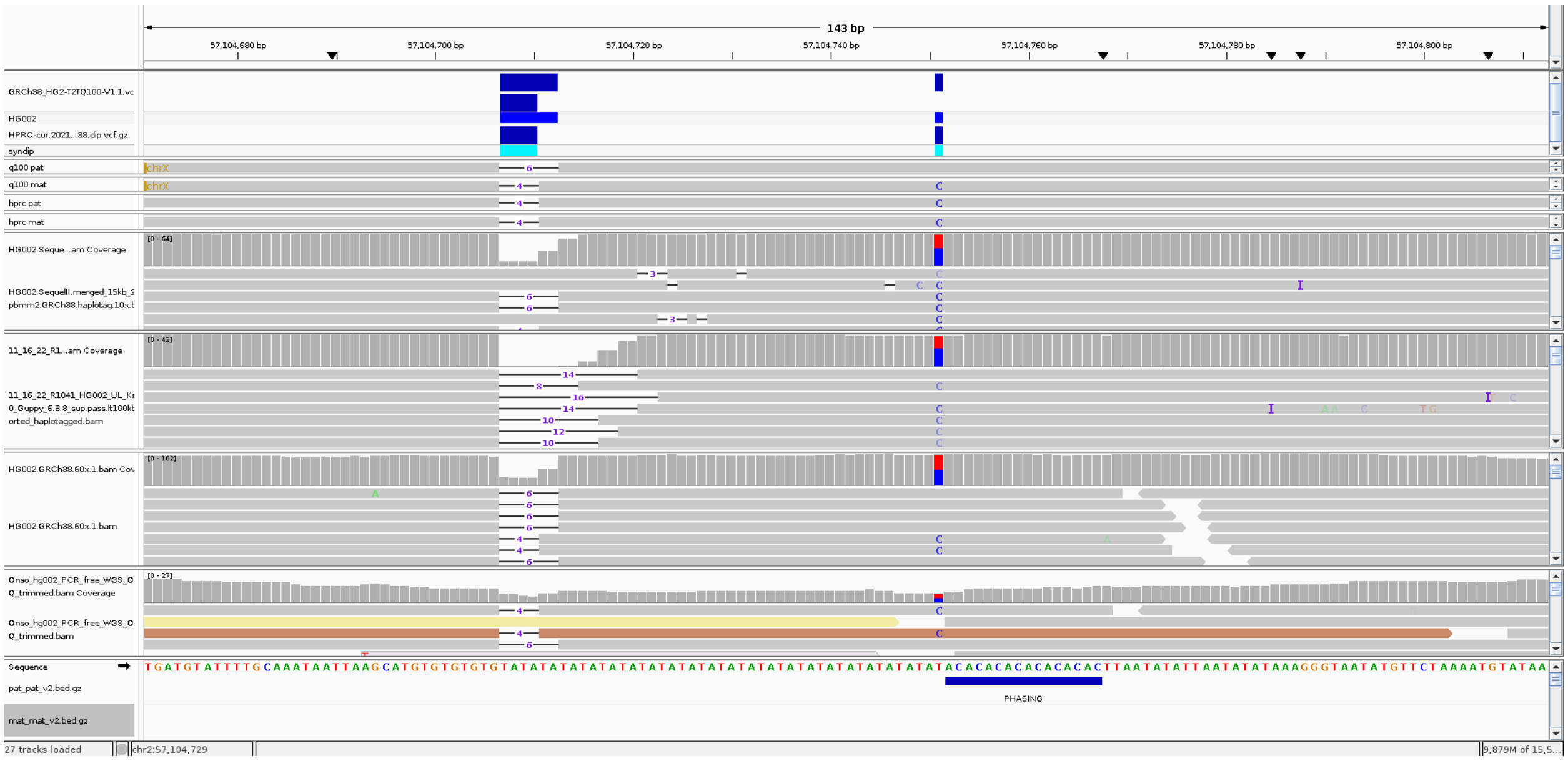

Figure S12: Example on chr2:57,104,564-57,104,848 (GRCh38), related to Figure 5. Shows a collapse genome error which is represented as 3 variants. The net effect is that AC is inserted on the HPRC paternal assembly relative to the paternal T2T-HG002v1.1 assembly. The three variants are described in **Table S19: Example of a complex variant.** The query deletes TATA relative to reference. The truth deletes TATA and converts the last T to a C on the maternal haplotype and deletes TATATA in the paternal haplotype. This complexity arises because the maternal T2T-HG002v1.1 haplotype and both HPRC haplotypes insert AC but remove TATA relative to GRCh38 reference.

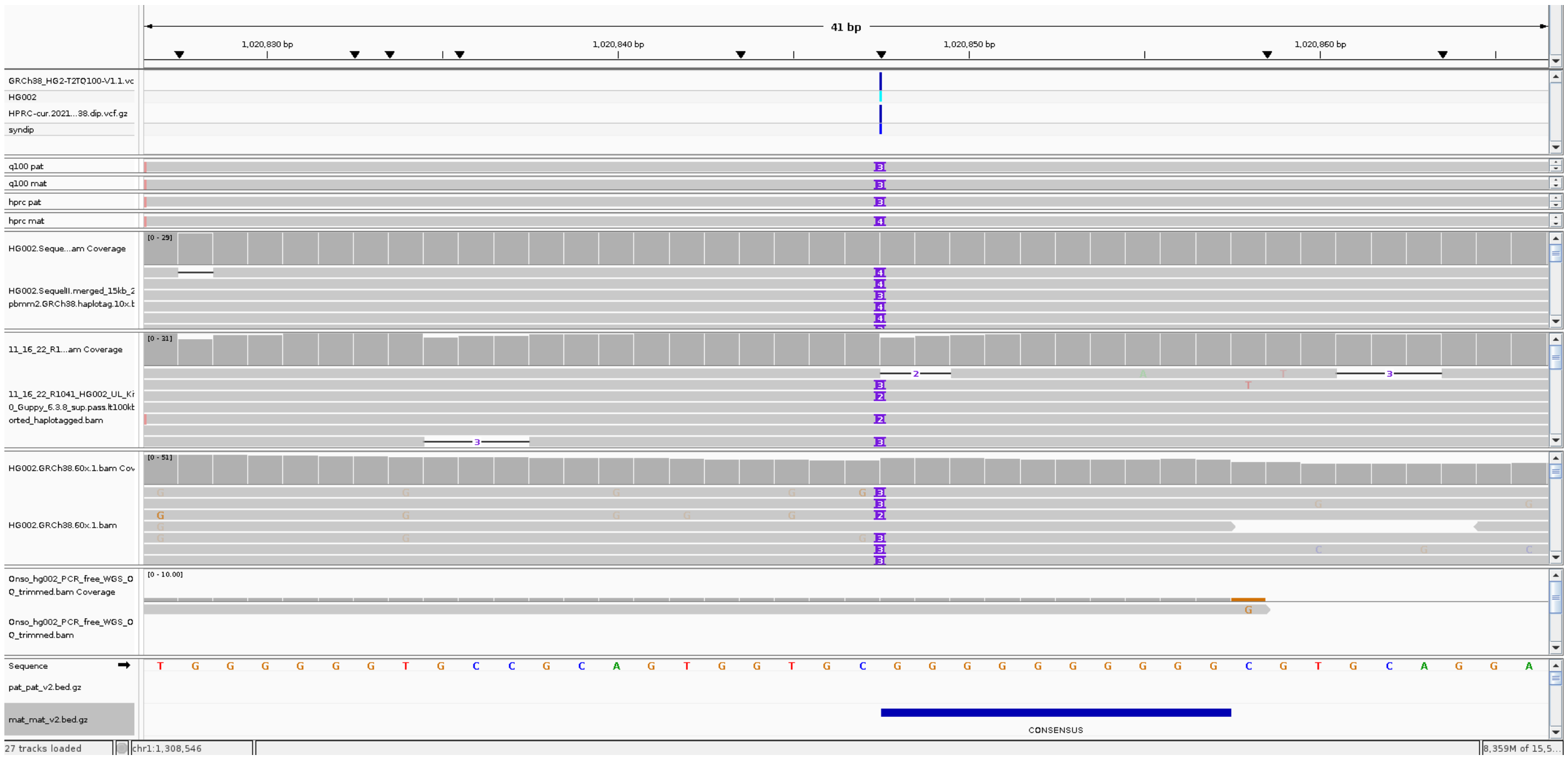

Figure S13: Example on chr1:1,020,827-1,020,866 (GRCh38), related to Figure 5. Shows collapse genome error which is equivalent to a FP and FN variant on the maternal haplotype. The net effect is that one G is inserted on the HPRC maternal assembly relative to the maternal haplotype of HG002v1.1.

Table S3: Corrections made in three rounds of polishing, related to STAR Methods. Patch counts include only patches that changed more than 50 base pairs in the corrected assembly. For this reaason, patch counts described in the manuscript are sometimes larger than in this table.

Single nucleotide corrections

	Count of Single Nuc Corrections	Base before correction	Base after correction
	240	A	C
	749	A	G
	489	A	T
	211	C	A
	196	C	G
	715	C	T
	763	G	A
	209	G	C
	314	G	T
	455	T	A
	795	T	C
	284	T	G
Total	5420		

Single nucleotide corrections made in each round

Round	Assembly being corrected	Number of single nucleotide corrections
Round 1	v0.7	2574
Round 2	v0.9	2555
Round 3	v1.0.1	291
	Total	5420

Small insertion corrections made in each round

Round	Assembly being corrected	Number of indel corrections
Round 1	v0.7	3441
Round 2	v0.9	1300
Round 3	v1.0.1	887
	Total	5628

Small deletion corrections made in each round

Round	Assembly being corrected	Number of indel corrections
Round 1	v0.7	21375

Round 2	v0.9	2583
Round 3	v1.0.1	2821
	Total	26779

Size and number of patches made in each round*

Round	Assembly being corrected	Number of regions corrected	Number of bases corrected
Round 1	v0.7	66	1112172
Round 2	v0.9	130	13045719
Round 3	v1.0.1	14	24913
	Total	210	14182804

Table S18. GenomeScope2.0 Statistics reported by 31-mers for each platform, related to STAR Methods. Statistics reported by 31-mers of each platform using GenomeScope2.0 and num. bps mapped with less than 2 reads with bwa or winnowmap2 on T2T-HG002v1.1.

Platform	Het (%)	Kcov (het. peak)	Error rate (%)	Model fit	Genome size est.	Bps with coverage < 2x
Element Aviti UltraQ	0.25%	34.0	0.06%	0.82	2,908,208,748	2,180,013
Illumina HiSeq 2x250	0.27%	30.7	0.26%	1.06	2,903,028,543	1,012,551
PacBio Onso	0.29%	22.5	0.03%	0.74	2,904,805,881	12,216,638
PacBio SPRQ on Revio	0.28%	50.6	0.08%	1.08	2,866,227,392	
PacBio HiFi on Revio	0.23%	34.0	0.09%	0.42	2,923,638,455	

Table S19. Example of a complex variant represented by only one genome error (in VCF format), related to STAR Methods.

Q100 benchmark

chr2	57104706	.	GTATA	G	30	.	.	GT:AD	0|1:0,1
chr2	57104706	.	GTATATA	G	30	.	.	GT:AD	1|0:0,1
chr2	57104751	.	T	C	30	.	.	GT:AD	0|1:1,1

HPRC Dipcall

chr2	57104706	.	GTATA	G	30	.	.	GT:AD	1|1:0
